# Synthesis of Structurally Diverse 2,3-Fused Indoles via Microwave-Assisted AgSbF_6_-Catalysed Intramolecular Difunctionalization of *o*-Alkynylanilines

**DOI:** 10.1038/srep13516

**Published:** 2015-08-27

**Authors:** Yuanqiong Huang, Yan Yang, Hongjian Song, Yuxiu Liu, Qingmin Wang

**Affiliations:** 1 State Key Laboratory of Elemento-Organic Chemistry, Research Institute of Elemento-Organic Chemistry, Collaborative Innovation Center of Chemical Science and Engineering (Tianjin), Nankai University, Tianjin 300071 China

## Abstract

2,3-Fused indoles are found in numerous natural products and drug molecules. Although several elegant methods for the synthesis of this structural motif have been reported, long reaction times and harsh conditions are sometimes required, and the yields tend to be low. Herein, we report a microwave method for straightforward access to various types of 2,3-fused indoles via AgSbF_6_-catalysed intramolecular difunctionalization of *o*-alkynylanilines. AgSbF_6_ played a role in both the hydroamination step and the imine-formation step. This method, which exhibited excellent chemoselectivity (no ring-fused 1,2-dihydroquinolines were formed), was used for formal syntheses of the natural products conolidine and ervaticine and the antihistamine drug latrepirdine.

Ring-fused indoles in general, and 2,3-fused indoles in particular, are found in numerous natural products and drug molecules (Fig. 1)^1^,^2^,^3^,^4^,^5^,^6^,^7^,^8^. Hexahydro-1*H*-azocino[4,3-*b*]indole is the core motif of numerous natural products, such as (+)-condylocarpine^9^, (+)-uleine^10^, conolidine and ervaticine^11^, (−)-actinophyllic acid^12^, and (−)-strychnine^13^. In addition, the clinical antihistamine drug latrepirdine contains a tetrahydro-1*H*-pyrido[4,3-*b*]indole skeleton (also known as *γ*-carboline)^14^. 2,3-Fused indoles have synthetically challenging structures and interesting biological activities, and much attention has been paid to the development of new approaches to the synthesis of this structural motif^15^,^16^,^17^,^18^,^19^. However, a practical method for the efficient synthesis of 2,3-fused indoles in a single operation is lacking.

Intramolecular difunctionalization of *o*-alkynylanilines is widely used to construct ring-fused indoles^20^,^21^,^22^, but this method, and other conventional synthetic methods, suffer from long reaction times, harsh conditions, and low yields. Microwave-assisted organic synthesis, which was first reported by Gedye *et al.* and Giguere *et al.* in 1986^23^,^24^, can be used to increase product yields and dramatically reduce reaction times compared to those of conventional synthetic methods^25^,^26^. Therefore, we wondered whether microwave-assisted intramolecular difunctionalization could be used to construct 2,3-fused indoles efficiently.

## Results

To evaluate this possibility, we initially carried out the reaction of *o*-alkynylaniline **1a** and benzaldehyde (2 equiv) with 10 mol% Sc(OTf)_3_ as a catalyst in 1,2-dichloroethane for 1.5 h at 80 °C at a microwave power of 100 W. These reaction conditions yielded indole **3aa′** as the sole product in 10% yield (Table 1, entry 1), and bicyclization product **3aa** was still not obtained when In(OTf)_3_ or Cu(OAc)_2_ was used instead of Sc(OTf)_3_ (entries 2 and 3). However, **3aa** was obtained in 94% yield when AgSbF_6_ was used as the catalyst (entry 4). The nature of the solvent greatly influenced the outcome of the reaction. No **3aa** formed when the solvent was 1,4-dioxane or acetonitrile, and the yield was only 54% in toluene (entries 5–7, respectively). Neither lowering the reaction temperature (entry 8) nor shortening the reaction time (entry 9) provided any benefit. Other silver(I) catalysts gave no better results than AgSbF_6_ (entries 10–12), and in the absence of microwaves, more than 30 h was required to give **3aa** in 86% yield (entry 13). These preliminary results indicated that the optimal reaction conditions were as follows: 10 mol% AgSbF_6_ in 1,2-dichloroethane at 80 °C (entry 4). This reaction exhibited excellent chemoselectivity: no ring-fused 1,2-dihydroquinoline **3aa′′** was obtained^27^.

We then used the optimal conditions to investigate the substrate scope of the reaction (Fig. 2). First, we carried out reactions of **1a** with various substituted benzaldehydes (**2a–2i**). The corresponding difunctionalization products (**3aa–3ai**) were obtained in more than 80% yield, indicating that neither the position nor the electronic properties of the substituents had a marked effect on the reaction outcome. We evaluated the electronic effects of substituents (R) on the benzene ring of **1** by carrying out reactions of **1a–1k** with **2j**. Substrates with no substituents, a weakly electron-withdrawing substituent (**1a–1e**), or an electron-donating substituent (**1f–1g**) afforded desired compounds **3aj–3gj** in more than 80% yield. Substrates with a strongly electron-withdrawing group, such as a cyano, trifluoromethyl, nitro, or methoxycarbonyl group, could also be converted to the desired products (**3hj–3kj**), albeit in slightly lower yields. Difunctionalization products **3ak–3ao** were obtained in moderate to good yields when **1a** was allowed to react with aliphatic aldehydes **2k–2o**, and the structures of **3aa** and **3ak** were confirmed by X-ray diffraction analysis^28^. Substrate **1a** reacted with aromatic aldehydes 2-naphthaldehyde (**3ap**, 81%), thiophene-2-carbaldehyde (**3aq**, 64%), furan-2-carbaldehyde (**3ar**, 36%), and piperonyl aldehyde (**3as**, 87%).

We also evaluated various nucleophilic moieties. The reaction worked well when the methylphenylsulfonyl group (XH = NHTs) was changed to a methanesulfonyl group (**3lj**, 72%), and tetrahydropyrano[4,3-*b*]indole (**3mj**) was obtained in 64% when XH was OH rather than NHTs. However, when YH was OH, intramolecular difunctionalization product **3nj** was not obtained, owing to the poor nucleophilicity of the phenolic hydroxyl group; instead, 5-*endo*-*trig* product **3nj′** resulting from a reaction in which water acted as a nucleophile was the sole product.

**Figure 1:**
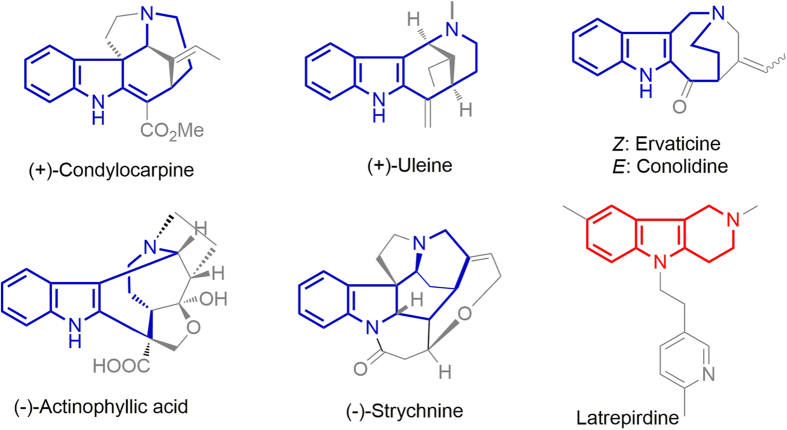
Representative compounds with 2,3-fused indole motifs.

We also investigated ring-closure reactions to form ring systems of various sizes (Fig. 3). Substrate **1o** (*n* = 1) reacted both with aromatic aldehydes (**2a**, **2d**, **2e**, and **2q**) and with aliphatic aldehydes (**2j**, **2l**, and **2n**) to afford the corresponding hexahydro-1*H*-azocino[4,3-*b*]indoles. We investigated both a one-pot method (Method A) and a stepwise method (Method B) and found that the latter gave higher yields. When CF_3_COOH was added to the reaction mixture (Method C), indoles fused to seven- to nine-membered-rings (**3ok**, **3pk**, and **3qk**) were obtained; **3pk** (hexahydro-1*H*-azocino[4,3-*b*]indole) is the core skeleton of many natural products (Fig. 1). The structures of **3pk** and **3oq** were confirmed by X-ray diffraction analysis^28^.

Plausible pathways for the difunctionalization reactions between *n*-butanal and **1a** (YH = NHTs) and **1n** (YH = OH) are depicted in Fig. 4a. Pathway **1** involves initial closure of the B ring to form **3aa′** via an AgSbF_6_-catalysed hydroamination reaction. Subsequently, AgSbF_6_ promotes the formation of imine **A**^29^, which is further transformed to target compound **3aj** via a 6-*endo*-*trig* cyclization. In this pathway, AgSbF_6_ catalyses both the hydroamination step and the formation of imine **A**. In pathway 2, an initial AgSbF_6_-catalysed imination reaction results in the formation of imine **B**, which can undergo two possible cyclizations: (1) 6-*endo*-*trig* cyclization leading to intermediate **C**, which can then be transformed to **3aj** or (2) 5-*endo*-*trig* cyclization (when YH = OH) leading to intermediate **D**, which can afford pyrrolidine **3nj′** by means of the addition of water. A third reaction pathway, leading to ring-fused 1,2-dihydroquinoline **3aa′′** via *5-endo-dig* cyclization, is also possible; however, no **3aa′′** was obtained from the reaction.

**Figure 2:**
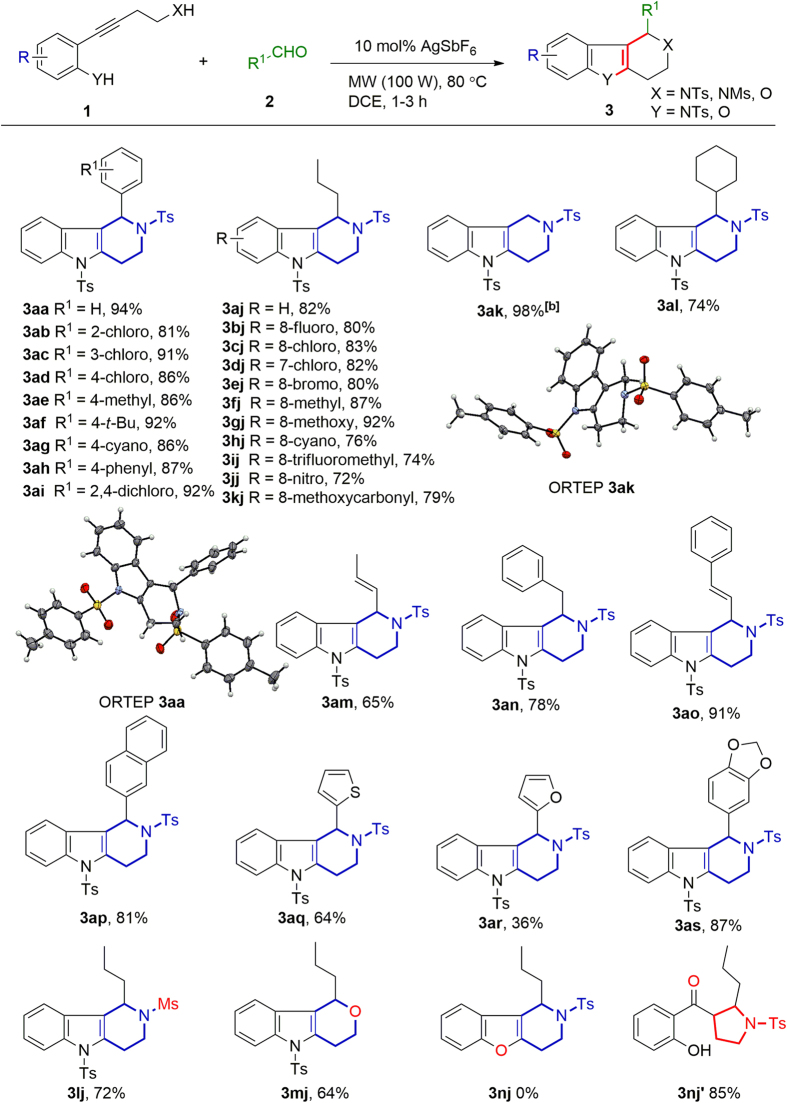
Synthesis of tetrahydro-1*H*-pyrido[4,3-*b*]indole and tetrahydropyrano[4,3-*b*]indole by intramolecular difunctionalization^[a]^. ^[a]^The yields given are isolated yields. DCE = 1,2-dichloroethane, Ts = 4-methylphenylsulfonyl, Ms = methanesulfonyl, MW = microwave. ^[b]^The reaction was conducted with **1a** (1 equiv) in the presence of AgSbF_6_ (10 mol%) in DCE at a MW power of 100 W for 1 h at 80 °C. Then **2k** (2 equiv) and CF_3_COOH (1.5 equiv) were added, and the mixture was reacted under the same conditions for 3 h.

**Figure 3:**
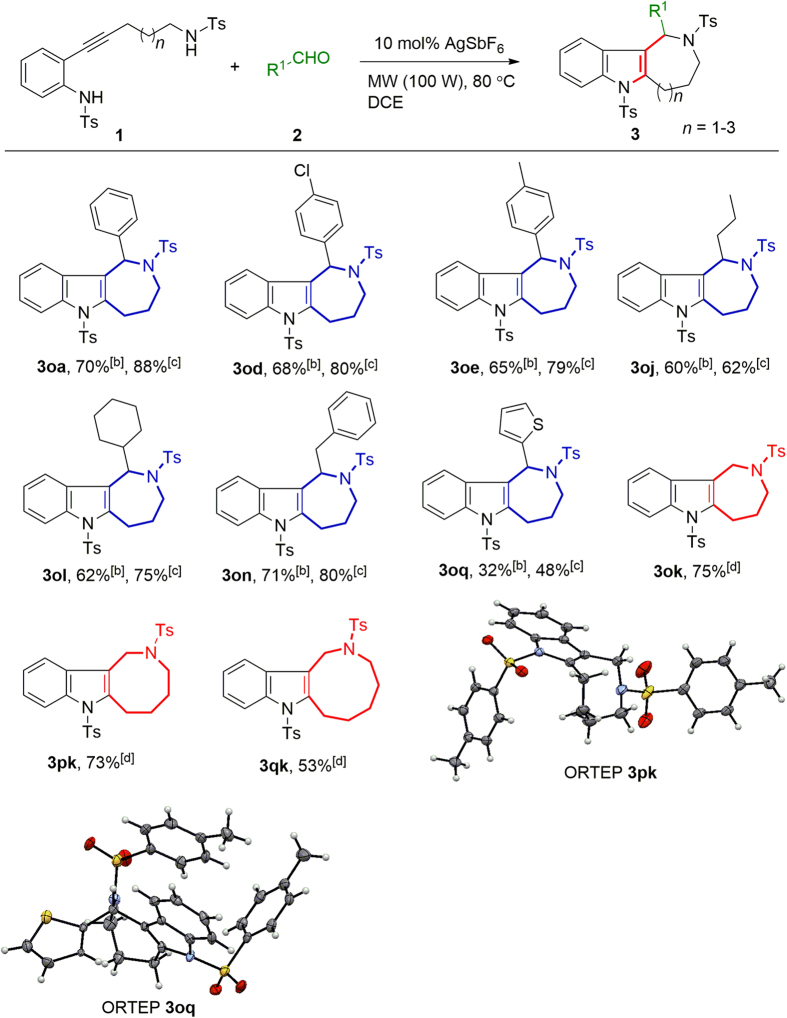
Synthesis of indoles fused to saturated medium-sized *N*-containing rings by means of intramolecular difunctionalization^[a]^. ^[a]^The yields given are isolated yields. DCE = 1,2-dichloroethane, MW = microwave. ^[b]^Method A: The reaction was conducted with **1** (1 equiv) and **2** (2 equiv) in the presence of AgSbF_6_ (10 mol%) at a MW power of 100 W in DCE for 6 h at 80 °C. ^[c]^Method B: The reaction was conducted with **1** (1 equiv) in the presence of AgSbF_6_ (10 mol%) at a MW power of 100 W in DCE for 3 h at 80 °C. Then **2** (2 equiv) was added, and the mixture was allowed to react under the same conditions for another 3 h. ^[d]^Method C: Similar to method B, except that CF_3_COOH (1.5 equiv) was added along with **2** (2 equiv) in the second step.

**Figure 4:**
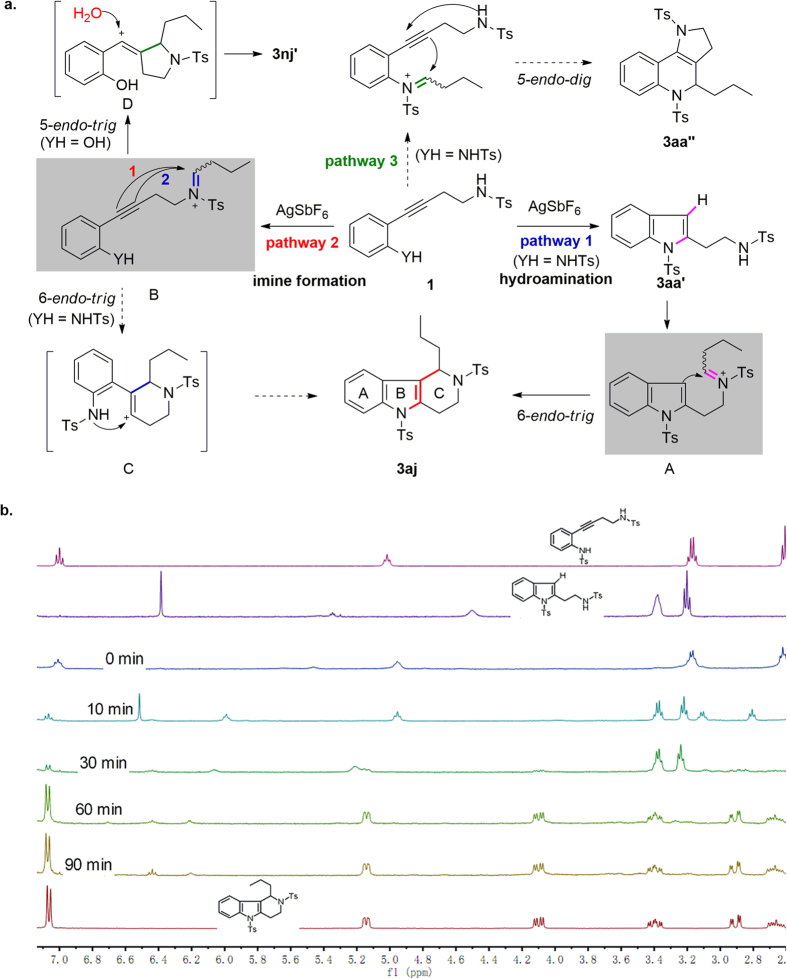
(**a**) Plausible reaction pathways and (**b**) time course of ^1^H NMR spectra measured during synthesis of 3aj.

To determine which of these pathways occurred under our reaction conditions, we monitored the formation of **3aj** over time by means of ^1^H NMR spectroscopy (Fig. 4b). The ^1^H NMR spectrum of **1a** exhibited a triplet at *δ* = 2.61 ppm and a quartet at *δ* = 3.17 ppm. After 10 min of reaction under the optimal conditions (Table 1, entry 4), a new singlet (*δ* = 6.38 ppm), attributable to the hydrogen at the 3-position of indole **3aa′** (Fig. 2a), was observed, along with a new triplet (*δ* = 3.22 ppm) and a new quartet (*δ* = 3.38 ppm), which are attributable to the two CH_2_ protons of **3aa′**. Note also that at this stage, the triplet and quartet attributable to **1a** shifted from 2.61 and 3.17 ppm to 2.80 and 3.10 ppm, respectively, owing to the formation of a complex between **1a** and AgSbF_6_. At 30 min, all of the complex had been transformed to **3aa′**. As the reaction progressed, the signals for **3aa′** disappeared gradually, and signals due to **3aj** appeared and increased in intensity. The reaction was almost complete after 60 min. These ^1^H NMR spectroscopy results suggest that the intramolecular difunctionalization reaction of **1a** occurred via pathway **1** in Fig. 4a.

**Figure 5:**
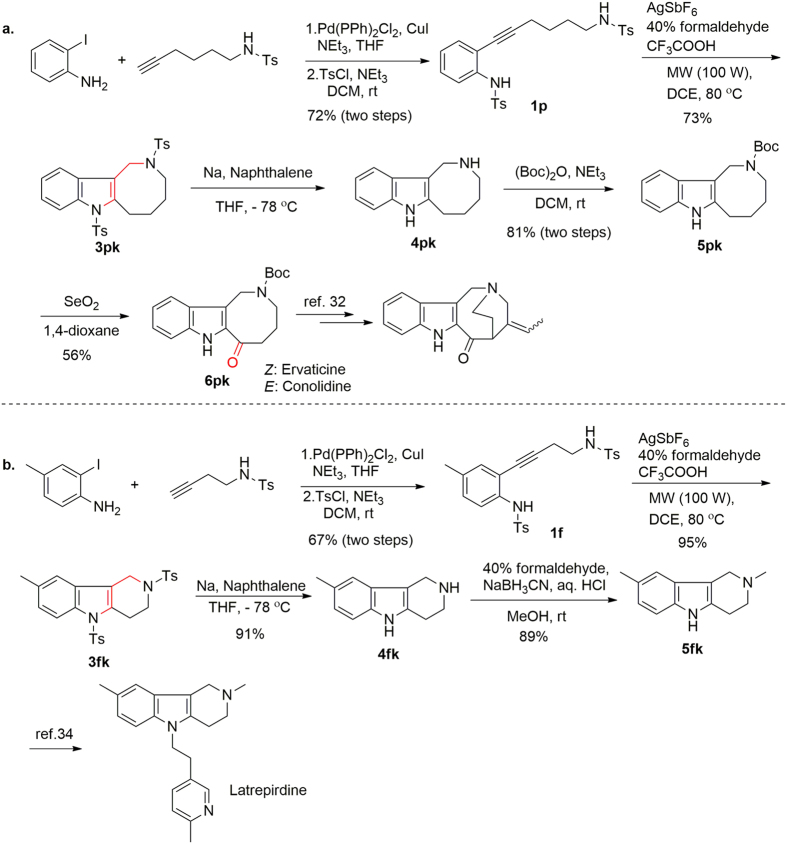
Formal syntheses of (a) ervaticine and conolidine and (b) latrepirdine.

Because our intramolecular difunctionalization method efficiently gave hexahydro-1*H*-azocino[4,3-*b*]indole **3pk**, we used the method for the rapid formal synthesis of the natural products ervaticine and conolidine as follows (Fig. 5a). Reaction of 2-iodoaniline with Ts-protected hex-5-yn-1-amine gave the corresponding coupled product, which reacted with 4-methylbenzene-1-sulfonyl chloride to give **1p** in 72% yield for the two steps^27^. Exposure of **1p** to the optimal difunctionalization reaction conditions afforded **3pk** in 73% yield. Removal of the *N*-tosyl group with sodium and naphthalene^30^ followed by protection with Boc_2_O provided **5pk** in 81% over two steps, and subsequent oxidation with SeO_2_ afforded **6pk** (56% yield)^31^, which has been converted to ervaticine and conolidine as described previously^32^.

We also used our method for a formal synthesis of latrepirdine as follows (Fig. 5b). Compound **1f** was obtained by Sonogashira coupling and subsequent protection of the primary amine with 4-methylbenzene-1-sulfonyl chloride in 67% yield for the two steps. Reaction between **1f** and formaldehyde by means of the method described for the synthesis of **3ak** (Fig. 2) gave **3fk** in 95% yield. Removal of the *N*-tosyl group^30^ and subsequent reductive amination^33^ afforded **5fk** (81% yield over two steps), which could be converted to latrepirdine as reported in the literature^34^.

In summary, we developed a straightforward method for accessing various 2,3-fused indoles via microwave-assisted AgSbF_6_-catalysed intramolecular difunctionalization of *o*-alkynylanilines. The reaction exhibited excellent chemoselectivity: no ring-fused 1,2-dihydroquinolines were formed. In addition to indoles fused to six-membered rings, indoles fused to saturated medium-sized *N*-containing rings could also be constructed. We used the method for efficient formal syntheses of the indole alkaloids ervaticine and conolidine and the antihistamine drug latrepirdine.

## Methods

### One-pot synthesis of 3aa

A microwave vessel was charged with *o*-alkynylaniline **1a** (98.5 mg, 0.21 mmol, 1 equiv), AgSbF_6_ (7.1 mg, 0.021 mmol, 0.1 equiv), aldehyde **2a** (26.7 mg, 0.25 mmol, 1.2 equiv), and DCE (5.0 mL); and the mixture was heated at a microwave power of 100 W at 80 °C for 1.5 h. After the reaction mixture cooled to room temperature, CH_2_Cl_2_ (10 mL) and H_2_O (10 mL) were added. The organic layer was separated, dried over anhydrous Na_2_SO_4_, and filtered. The filtrate was evaporated, and the residue was purified by silica gel chromatography with 10:1 (v/v) petroleum ether/ethyl acetate as the eluent to afford **3oa** (110.0 mg, 94%) as a white solid (see Supplementary Information).

### Stepwise synthesis of 3pk

A microwave vessel was charged with **1p** (100.0 mg, 0.20 mmol, 1 equiv), AgSbF_6_ (6.8 mg, 0.02 mmol, 0.1 equiv), and DCE (5.0 mL) in that order; and the mixture was heated at a microwave power of 100 W at 80 °C for 3 h. Then **2k** (40% formaldehyde, 30.0 mg, 0.40 mmol, 2.0 equiv) and CF_3_COOH (34.2 mg, 0.30 mmol, 1.5 equiv) were added, and the mixture was allowed to react under the same conditions for another 1 h. CH_2_Cl_2_ (10 mL) and H_2_O (10 mL) were added to the resulting mixture. The organic layer was separated, dried over anhydrous Na_2_SO_4_, and filtered. The filtrate was evaporated, and the residue was purified by silica gel chromatography with 10:1 (v/v) petroleum ether/ethyl acetate as the eluent to afford **3pk** (74.2 mg, 73%) as a white solid.

## Additional Information

**How to cite this article**: Huang, Y. *et al.* Synthesis of Structurally Diverse 2,3-Fused Indoles via Microwave-Assisted AgSbF_6_-Catalysed Intramolecular Difunctionalization of *o*-Alkynylanilines. *Sci. Rep.* **5**, 13516; doi: 10.1038/srep13516 (2015).

## Supplementary Material

Supplementary Information

Supplementary File 1

Supplementary File 2

Supplementary File 3

Supplementary File 4

## Figures and Tables

**Table 1:**
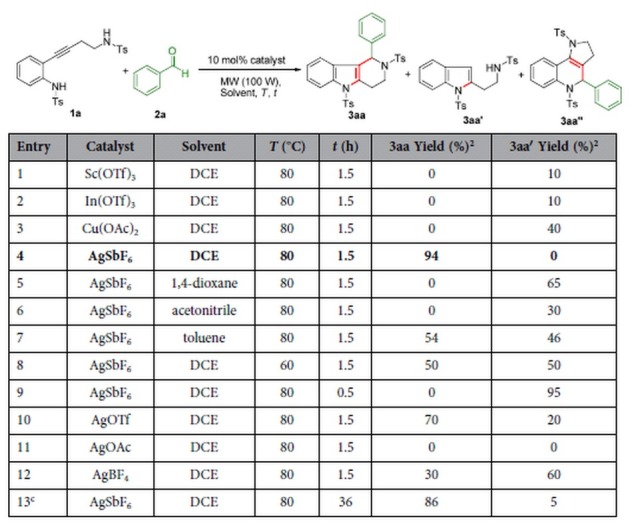
Optimization of reaction conditions^a^.

^a^The reaction was conducted with **1a** (1 equiv) and **2a** (1.2 equiv) in the presence of catalyst (10 mol%) under microwave conditions in 1,2-dichloroethane (DCE) for 1–3 h at 80 °C, unless otherwise stated. Ts = 4-methylphenylsulfonyl.

^b^The yields given are isolated yields.

^c^This reaction was conducted without microwave assistance.
