# Supplementary material for: Synthesis of Structurally Diverse 2,3-Fused Indoles via Microwave-Assisted AgSbF6-Catalysed Intramolecular Difunctionalization of o-Alkynylanilines
Source: Sci Rep. 2015 Aug 27;5:13516. doi: 10.1038/srep13516 (PMC4550932; doi:10.1038/srep13516)

Synthesis of Structurally Diverse 2,3-Fused Indoles via Microwave-Assisted AgSbF_6_-Catalyzed Intramolecular Difunctionalization of *o*-Alkynylanilines

Yuanqiong Huang,^†^ Yan Yang,^†^ Hongjian Song,* Yuxiu Liu and Qingmin Wang*

State Key Laboratory of Elemento-Organic Chemistry, Research Institute of Elemento-Organic Chemistry, Collaborative Innovation Center of Chemical Science and Engineering (Tianjin), Nankai University, Tianjin 300071 (China).

Table of contents…………………………………………………………..……...….S1

General Information…………………………………………………………....…….S2

The general synthetic route of substrates **1**……………………………………....S2–S3

Data of **1a–1p**………..……...…………………………………………….….….S3–S9

General procedure of AgSbF6-catalyzed chemoselective intramolecular difunctionalization of alkynes to afford 2,3-fused indoles……………..……...…......S9

Data of **3aa′**, **3aa–3qk**.........................................................................................S9–S25

Formal synthesis of eraticine and conolidine.....................................................S25–S27

Formal synthesis of latrepirdine........................................................................S27–S28

References………………………………………............................…………….…S28

The ORTEP, crystal data and structure refinement of 3aa, 3ak, 3pk, and 3oq…………………………………………………………………….....……S29–S32

Copies of ^1^H, ^13^C spectra……………...……………………………….…..….S32–S98

General Information: All reagents were used as received. PdCl_2_(PPh_3_)_2_, PPh_3_ and CuI was purchased from Boka Chem. Co. Ltd; Sc(OTf)_3_, In(OTf)_3_, Cu(OAc)_2_, AgSbF_6_, AgOTf, AgOAc, AgBF_4_ and AgSbF_6_ were purchased from Sigma-Aldrich Co. LLC. 1,2-Dichloroethane (DCE) and acetonitrile were distilled on phosphorus pentoxide; toluene was dried by metallic sodium. The intramolecular difunctionalization reaction was carried out in a microwave synthesis system (80 ºC, 100 W, Discover S-Class, CEM). ^1^H and ^13^C Nuclear Magnetic Resonance (NMR) spectra were recorded on a Bruker Avance 400 Ultrashield NMR spectrometers. High-resolution mass spectrometry (HRMS) data were obtained on an FTICR-MS instrument (Ionspec 7.0 T). Single crystal X-ray structure data were collected on Rigaku 007 Saturn 70. The melting points were determined on an X-4 binocular microscope melting point apparatus (Beijing Tech Instruments Co., Beijing, China) and are uncorrected.

**The general synthetic route of substrates 1**

To a three-necked flask were added Pd(PPh3)2Cl2 (3.0 mol%), CuI (3.0 mol%), **A** (1equiv), Et3N (2 equiv) and THF. After degassing with argon and four evacuation/backfill-cycles with argon, **B**^1^ (1.2 equiv) in tetrahydrofuran was added dropwise. The reaction mixture was stirred at room temperature. When the reaction was complete as monitored by TLC, H_2_O was added to the resulting mixture. After separation of the organic layer, the water layer was extracted with DCM. The combined organic layer was dried over anhydrous Na_2_SO_4_, filtered, concentrated in vacuo to give **C**^2^ which was used without further purification.

To a flask were added **C** (1equiv) in DCM, then pyridine (3 equiv) and TsCl (2 equiv) were added sequentially at room temperature, and the reaction mixture was stirred at room temperature. When the reaction was complete as monitored by TLC, H_2_O was added to the resulting mixture. After separation of the organic layer, the water layer was extracted with DCM. The combined organic layer was dried over anhydrous Na_2_SO_4_, filtered, evaporated and purified via column chromatography on silica gel (eluent: petroleum ether/ethyl acetate) to afford the desired product **1**.

**4-Methyl-*N*-(2-(4-(4-methylphenylsulfonamido)but-1-yn-1-yl)phenyl)benzenesulfonamide (1a)**

White powder, yield 82%, Mp 48–49 ºC. ^1^H NMR (400 MHz, CDCl_3_) *δ* 7.78 (d, *J* = 8.0 Hz, 2H), 7.69 (d, *J* = 8.0 Hz, 2H), 7.49 (d, *J* = 8.4 Hz, 1H), 7.30 (d, *J* = 8.0 Hz, 2H), 7.25 – 7.23(m, 1H), 7.22 (d, *J* = 7.6 Hz, 4H), 7.00 (t, *J* = 7.6 Hz, 1H), 5.02 (t, *J* = 6.4 Hz, 1H), 3.17 (q, *J* = 6.4 Hz, 2H), 2.61 (t, *J* = 6.4 Hz, 2H), 2.42 (s, 3H), 2.37 (s, 3H); ^13^C NMR (100 MHz, CDCl_3_) *δ* 144.1, 143.7, 137.7, 137.0, 136.2, 132.2, 129.9, 129.7, 129.3, 127.3, 127.1, 124.4, 120.0, 114.5, 93.2,77.6, 41.8, 29.7,21.6, 21.2; HRMS (ESI) calcd for C_24_H_24_N_2_O_4_S_2_ [M+H]^+^ 469.1250, found 469.1257.

***N*-(4-fluoro-2-(4-(4-methylphenylsulfonamido)but-1-yn-1-yl)phenyl)-4-methylbenzenesulfonamide (1b)**

White powder, Yield 76%, Mp 120–122 ºC. ^1^H NMR (400 MHz, CDCl_3_) *δ* 7.78 (d, *J* = 8.4 Hz, 2H), 7.64 (d, *J* = 8.0 Hz, 2H), 7.46 (dd, *J* = 9.2, 5.2 Hz,1H), 7.31 (d, *J* = 8.0 Hz, 2H), 7.22 (d, *J* = 8.0 Hz, 2H), 7.11 (s, 1H), 7.00 – 6.93 (m, 1H), 6.90 (dd, *J* = 8.8, 3.2 Hz, 1H), 5.08 (t, *J* = 6.4 Hz, 1H), 3.15 (q, *J* = 6.4 Hz, 2H), 2.59 (t, *J* = 6.4 Hz, 2H), 2.42 (s, 3H), 2.38 (s, 3H); ^13^C NMR (100 MHz, CDCl_3_) *δ* 160.6, 158.1, (144.2, 143.7), 136.9, 136.0, (133.9, 133.8), 129.9, 129.7, 127.3, 127.1, (123.4, 123.3), (118.7, 118.4), (117.4, 117.3), (116.6, 116.4), 94.0, 76.7, 41.6, 21.6, 21.5, 21.2; HRMS (ESI) calcd for C_24_H_23_FN_2_O_4_S_2_ [M+H]^+^ 487.1156, found 487.1162.

***N*-(4-chloro-2-(4-(4-methylphenylsulfonamido)but-1-yn-1-yl)phenyl)-4-methylbenzenesulfonamide (1c)**

White powder, Yield 78%, Mp 118–120 ºC. ^1^H NMR (400 MHz, CDCl_3_) *δ* 7.78 (d, *J* = 8.0 Hz, 2H), 7.68 (d, *J* = 8.4 Hz, 2H), 7.43 (d, *J* = 8.8 Hz, 1H), 7.31 (d, *J* = 8.4 Hz, 2H), 7.25 (s, 1H), 7.24 – 7.16 (m, 4H), 5.02 (t, *J* = 6.4 Hz, 1H), 3.17 (q, *J* = 6.4 Hz, 2H), 2.61 (t, *J* = 6.4 Hz, 2H), 2.42 (s, 3H), 2.38 (s, 3H); ^13^C NMR (100 MHz, CDCl_3_) *δ* 144.4, 143.8, 136.9, 136.4, 136.0, 131.7, 129.9, 129.8, 129.7, 129.5, 127.3, 127.1, 121.4, 116.2, 94.4, 76.6, 41.7, 21.6, 21.6, 21.3; HRMS (ESI) calcd for C_24_H_23_ClN_2_O_4_S_2_ [M+H]^+^ 503.0861, found 503.0859.

***N*-(5-chloro-2-(4-(4-methylphenylsulfonamido)but-1-yn-1-yl)phenyl)-4-methylbenzenesulfonamide (1d)**

White powder, Yield 76%, Mp 72–73 ºC. ^1^H NMR (400 MHz, CDCl_3_) *δ* 7.78 (d, *J* = 8.4 Hz, 2H), 7.73 (d, *J* = 8.0 Hz, 2H), 7.51 (d, *J* = 2.0 Hz, 1H), 7.30 (d, *J* = 8.0 Hz, 3H), 7.27– 7.23 (m, 2H), 7.14 (d, *J* = 8.4 Hz, 1H), 6.96 (dd, *J* = 8.4, 2.0 Hz, 1H), 5.04 (t, *J* = 6.4 Hz, 1H), 3.17 (q, *J* = 6.4 Hz, 2H), 2.63 (t, *J* = 6.4 Hz, 2H), 2.42 (s, 3H), 2.38 (s, 3H); ^13^C NMR (100 MHz, CDCl_3_) *δ* 144.4, 143.7, 138.8, 136.9, 135.9, 135.0, 133.0, 129.9, 129.8, 127.3, 127.1, 124.5, 119.8, 112.7, 94.4, 76.6, 41.7, 21.6, 21.5, 21.3; HRMS (ESI) calcd for C_24_H_23_ClN_2_O_4_S_2_ [M+H]^+^ 503.0861, found 503.0862.

***N*-(4-bromo-2-(4-(4-methylphenylsulfonamido)but-1-yn-1-yl)phenyl)-4-methylbenzenesulfonamide (1e)**

White powder, Yield 79%, Mp 155–157 ºC. ^1^H NMR (400 MHz, CDCl_3_) *δ* 7.78 (d, *J* = 8.0 Hz, 2H), 7.68 (d, *J* = 8.0 Hz, 2H), 7.44 – 7.29 (m, 5H), 7.28 – 7.18 (m, 3H), 4.98 (t, *J* = 6.0 Hz, 1H), 3.17 (q, *J* = 6.4 Hz, 2H), 2.62 (t, *J* = 6.4 Hz, 2H), 2.42 (s, 3H), 2.38 (s, 3H); ^13^C NMR (100 MHz, CDCl_3_) *δ* 144.4, 143.8, 136.9, 135.9, 134.6, 132.3, 129.9, 129.84, 129.80, 127.3, 127.1, 121.5, 117.1, 116.5, 94.7, 76.3, 41.7, 21.59, 21.57, 21.3; HRMS (ESI) calcd for C_24_H_23_BrN_2_O_4_S_2_ [M+H]^+^ 547.0355, found 547.0353.

**4-Methyl-*N*-(4-methyl-2-(4-(4-methylphenylsulfonamido)but-1-yn-1-yl)phenyl)benzenesulfonamide (1f)**

White powder, Yield 75%, Mp 135–136 ºC. ^1^H NMR (400 MHz, CDCl_3_) *δ* 7.78 (d, *J* = 8.4 Hz, 2H), 7.65 (d, *J* = 8.0 Hz, 2H), 7.37 (d, *J* = 8.8 Hz, 1H), 7.31 (d, *J* = 8.0 Hz, 2H), 7.21 (d, *J* = 8.0 Hz, 2H), 7.07– 7.02 (m, 3H), 4.95 (t, *J* = 6.4 Hz, 1H), 3.15 (q, *J* = 6.4 Hz, 2H), 2.57 (t, *J* = 6.4 Hz, 2H), 2.42 (s, 3H), 2.37 (s, 3H), 2.23 (s, 3H) ; ^13^C NMR (100 MHz, CDCl_3_) *δ* 144.0, 143.7, 137.0, 136.2, 135.1, 134.4, 132.5, 130.1, 129.9, 129.6, 127.3, 127.1, 120.8, 114.9, 92.5, 77.8, 41.8, 21.6, 21.2, 20.6; HRMS (ESI) calcd for C_25_H_26_N_2_O_4_S_2_ [M+H]^+^ 483.1407, found 483.1413.

***N*-(4-methoxy-2-(4-(4-methylphenylsulfonamido)but-1-yn-1-yl)phenyl)-4-methylbenzenesulfonamide (1g)**

White powder, Yield 80%, Mp 112–113 ºC. ^1^H NMR (400 MHz, CDCl_3_) *δ* 7.78 (d, *J* = 8.0 Hz, 2H), 7.59 (d, *J* = 8.0 Hz, 2H), 7.39 (d, *J* = 9.2 Hz, 1H), 7.30 (d, *J* = 8.0 Hz, 2H), 7.19 (d, *J* = 8.0 Hz, 2H), 6.94 (s, 1H), 6.81 (dd, *J* = 9.2, 2.8 Hz, 1H), 6.72 (d, *J* = 2.8 Hz, 1H), 5.17 (t, *J* = 6.4 Hz, 1H), 3.73 (s, 3H), 3.13 (q, *J* = 6.4 Hz, 2H), 2.54 (t, *J* = 6.4 Hz, 2H), 2.41 (s, 3H), 2.36 (s, 3H); ^13^C NMR (100 MHz, CDCl_3_) *δ* 156.9, 143.9, 143.6, 137.0, 136.2, 130.6, 129.8, 129.5, 127.3, 127.1, 124.3, 117.7, 116.5, 115.6, 92.5, 77.6, 55.5, 41.7, 21.54, 21.53, 21.2; HRMS (ESI) calcd for C_25_H_26_N_2_O_5_S_2_ [M+H]^+^ 499.1356, found 499.1367.

***N*-(4-cyano-2-(4-(4-methylphenylsulfonamido)but-1-yn-1-yl)phenyl)-4-methylbenzenesulfonamide (1h)**

White powder, Yield 82%, Mp 141–142 ºC. ^1^H NMR (400 MHz, CDCl_3_) *δ* 7.87 – 7.71 (m, 5H), 7.60 – 7.41 (m, 3H), 7.34– 7.29 (m, 2H), 7.27 (d, *J* = 4.4 Hz, 2H), 5.19 (t, *J* = 6.4 Hz, 1H), 3.21 (q, *J* = 6.4 Hz, 2H), 2.70 (t, *J* = 6.4 Hz, 2H), 2.42 (s, 3H), 2.39 (s, 3H); ^13^C NMR (100 MHz, CDCl_3_) *δ* 144.9, 143.9, 141.7, 136.8, 136.0, 135.7, 132.7, 130.1, 129.9, 127.4, 127.1, 118.0, 117.8, 114.1, 107.2, 96.3, 75.5, 41.7, 21.63, 21.57, 21.5; HRMS (ESI) calcd for C_25_H_23_N_3_O_4_S_2_ [M+H]^+^ 494.1203, found 494.1207.

**4-Methyl-*N*-(4-(2-(4-methylphenylsulfonamido)-5-(trifluoromethyl)phenyl)but-3-yn-1-yl)benzenesulfonamide (1i)**

White powder, Yield 72%, Mp 155–156 ºC. ^1^H NMR (400 MHz, CDCl_3_) *δ* 7.78 (t, *J* = 6.8 Hz, 4H), 7.61 – 7.53 (m, 2H), 7.49 (s, 1H), 7.44 (d, *J* = 8.4 Hz, 1H), 7.36 – 7.26 (m, 4H), 5.05 (t, *J* = 6.4 Hz,1H), 3.21 (q, *J* = 6.4 Hz, 2H), 2.68 (t, *J* = 6.4 Hz, 2H), 2.41 (s, 3H), 2.39 (s, 3H) ; ^13^C NMR (100 MHz, CDCl_3_) *δ* 139.9, 139.0, 136.0, 132.1, 131.2, 125.2, 125.1, 124.6 (q, *J_C-F_* = 3.6 Hz), 122.6, 122.4, 121.2 (q, *J_C-F_* = 65.8 Hz), 121.3 (q, *J_C-F_* = 3.6 Hz), 118.7 (q, *J_C-F_* = 270.5 Hz), 113.6, 109.2, 90.5, 71.5, 37.0, 16.8, 16.7, 16.6; HRMS (ESI) calcd for C_25_H_23_F_3_N_2_O_4_S_2_ [M+H]^+^ 537.1124, found 537.1126.

**4-Methyl-*N*-(4-(2-(4-methylphenylsulfonamido)-5-nitrophenyl)but-3-yn-1-yl)benzenesulfonamide (1j)**

White solid, Yield 47%, Mp 64–65 ºC. ^1^H NMR (400 MHz, CDCl_3_) *δ* 8.11 (s, 1H), 8.06 (d, *J* = 9.6 Hz, 1H), 7.89 (s, 1H), 7.83 (d, *J* = 8.0 Hz, 2H), 7.80 (d, *J* = 8.0 Hz, 2H), 7.57 (d, *J* = 9.2 Hz, 1H), 7.31 (t, *J* = 8.4 Hz, 4H), 5.15 (t, *J* = 6.0 Hz, 1H), 3.22 (q, *J* = 6.4 Hz, 2H), 2.73 (t, *J* = 6.4 Hz, 2H), 2.42 (s, 3H), 2.39 (s, 3H); ^13^C NMR (100 MHz, CDCl_3_) *δ* 145.2, 144.0, 143.5, 143.1, 136.9, 135.7, 130.2, 130.1, 127.9, 127.6, 127.2, 124.8, 117.1, 113.5, 96.5, 75.8, 41.8, 21.8, 21.7, 21.6; HRMS (ESI) calcd for C_24_H_24_N_3_O_6_S_2_ [M+H]^+^ 514.1101, found 514.1105.

**Methyl 4-(4-methylphenylsulfonamido)-3-(4-(4-methylphenylsulfonamido)but-1-yn-1-yl)benzoate (1k)**

White solid, Yield 69%, Mp 46–48 ºC. ^1^H NMR (400 MHz, CDCl_3_) *δ* 7.91 (s, 1H), 7.87 (d, *J* = 8.8 Hz, 1H), 7.80 (d, *J* = 8.4 Hz, 2H), 7.77 (d, *J* = 8.0 Hz, 2H), 7.71 (s, 1H), 7.54 (d, *J* = 8.8 Hz, 1H), 7.30 (d, *J* = 6.8 Hz, 2H), 7.25 (d, *J* = 7.6 Hz, 2H), 5.46 (t, *J* = 6.0 Hz, 1H), 3.87 (s, 3H), 3.20 (q, *J* = 6.0 Hz, 2H), 2.67 (t, *J* = 6.4 Hz, 2H), 2.41 (s, 3H), 2.37 (s, 3H); ^13^C NMR (100 MHz, CDCl_3_) *δ* 165.9, 144.6, 143.8, 141.7, 136.9, 135.8, 133.9, 130.6, 130.0, 129.9, 127.4, 127.2, 127.1,125.6, 117.8, 113.5, 94.7, 76.5, 52.3, 41.8, 21.7, 21.6, 21.3; HRMS (ESI) calcd for C_26_H_27_N_2_O_6_S_2_ [M+H]^+^ 527.1305, found 527.1309.

**4-Methyl-*N*-(2-(4-(methylsulfonamido)but-1-yn-1-yl)phenyl)benzenesulfonamide (1l)**

White solid, Yield 78%, Mp 91–93 ºC. ^1^H NMR (400 MHz, CDCl_3_) *δ* 7.71 (d, *J* = 8.0 Hz, 2H), 7.45 (d, *J* = 8.0 Hz, 1H), 7.33 (s, 1H), 7.30 – 7.20 (m, 4H), 7.02 (t, *J* = 7.6 Hz, 1H), 4.94 (t, *J* = 6.0 Hz, 1H), 3.38 (q, *J* = 6.4 Hz, 1H), 3.03 (s, 3H), 2.74 (t, *J* = 6.4 Hz, 1H), 2.39 (s, 3H); ^13^C NMR (100 MHz, CDCl_3_) *δ* 144.3, 137.8, 136.2, 132.5, 129.8, 129.4, 127.5, 124.8, 120.8, 115.3, 93.5, 77.7, 42.0, 40.8, 21.9, 21.7; HRMS (ESI) calcd for C_18_H_21_N_2_O_4_S_2_ [M+H]^+^ 393.0937, found 393.0940.

***N*-(2-(4-hydroxybut-1-yn-1-yl)phenyl)-4-methylbenzenesulfonamide (1m)**

White solid, Yield 32%, Mp 64–65 ºC. ^1^H NMR (400 MHz, CDCl_3_) δ 7.70 (d, *J* = 8.0 Hz, 3H), 7.55 (d, *J* = 8.4 Hz, 1H), 7.28 – 7.18 (m, 4H), 6.98 (t, *J* = 7.6 Hz, 1H), 3.86 (t, *J* = 6.0 Hz, 2H), 2.70 (t, *J* = 6.0 Hz, 2H), 2.36 (s, 3H); ^13^C NMR (100 MHz, CDCl_3_) *δ* 144.1, 138.4, 136.4, 131.5, 129.7, 129.3, 127.4, 124.2, 119.4, 114.4, 94.8, 77.7, 60.9, 23.6, 21.7; HRMS (ESI) calcd for C_17_H_18_NO_3_S [M+H]^+^ 316.1002, found 316.0998.

***N*-(4-(2-hydroxyphenyl)but-3-yn-1-yl)-4-methylbenzenesulfonamide (1n)**

Oil, Yield 75%. ^1^H NMR (400 MHz, CDCl_3_) *δ* 7.78 (d, *J* = 8.4 Hz, 2H), 7.30 (d, *J* = 8.0 Hz, 2H), 7.24 (d, *J* = 1.6 Hz, 1H), 7.23 – 7.19 (m, 1H), 6.92 (d, *J* = 8.0 Hz, 1H), 6.84 (td, *J* = 7.6, 0.8 Hz, 1H), 6.01 (br, 1H), 5.05 (t, *J* = 6.0 Hz, 1H), 3.22 (q, *J* = 6.4 Hz, 1H), 2.65 (t, *J* = 6.4 Hz, 1H), 2.41 (s, 3H); ^13^C NMR (100 MHz, CDCl_3_) *δ* 157.0, 143.7, 136.9, 131.7, 130.1, 129.8, 127.1, 120.2, 114.9, 109.4, 92.7, 77.5, 41.9, 21.5, 21.2; HRMS (ESI) calcd for C_17_H_18_NO_3_S [M+H]^+^ 316.1002, found 316.1002.

**4-Methyl-*N*-(2-(5-(4-methylphenylsulfonamido)pent-1-yn-1-yl)phenyl)benzenesulfonamide (1o)**

White powder, yield 80%, Mp 101–103 ºC,^1^H NMR (400 MHz, CDCl_3_) *δ* 7.78 (d, *J* = 8.0 Hz, 2H), 7.67 (d, *J* = 8.4 Hz, 2H), 7.50 (d, *J* = 8.4 Hz, 1H), 7.32 – 7.26 (m, 3H), 7.20 (d, *J* = 8.0 Hz, 4H), 6.98 (t, *J* = 7.6 Hz, 1H), 5.01 (t, *J* = 6.4 Hz, 1H), 3.08 (q, *J* = 6.4 Hz, 2H), 2.48 (t, *J* = 6.8 Hz, 2H), 2.40 (s, 3H), 2.35 (s, 3H), 1.82 – 1.72(m, 2H); ^13^C NMR (100 MHz, CDCl_3_) *δ* 144.2, 143.6, 137.7, 136.9, 136.3, 132.2, 129.9, 129.7, 129.1, 127.4, 127.2, 124.4, 119.8, 114.9, 96.0, 76.4, 42.2, 28.5, 21.7, 21.6, 16.9; HRMS (ESI) calcd for C_25_H_26_N_2_O_4_S_2_ [M+H]^+^ 483.1407, found 483.1416.

**4-Methyl-*N*-(2-(6-(4-methylphenylsulfonamido)hex-1-yn-1-yl)phenyl)benzenesulfonamide (1p)**

White powder, yield 81%, Mp 102–103 ºC. ^1^H NMR (400 MHz, CDCl_3_) *δ* 7.76 (d, *J* = 8.4 Hz, 2H), 7.68 (d, *J* = 8.4 Hz, 2H), 7.49 (d, *J* = 8.0 Hz, 1H), 7.28 (d, *J* = 8.0 Hz, 2H), 7.25 – 7.18 (m, 5H), 6.97 (t, *J* = 7.6 Hz, 1H), 4.94 (t, *J* = 6.0 Hz, 1H), 2.99 (q, *J* = 6.4 Hz, 2H), 2.42 (t, *J* = 6.0 Hz, 2H), 2.40 (s, 3H), 2.36 (s, 3H), 1.75 – 1.56(m, 4H); ^13^C NMR (100 MHz, CDCl3) *δ* 144.3, 143.5, 137.7, 137.0, 136.2, 132.0, 129.9, 129.8, 129.1, 127.4, 127.2, 124.3, 119.1, 114.6, 97.1, 76.1, 42.9, 29.0, 25.6, 21.7, 21.6, 19.2; HRMS (ESI) calcd for C_26_H_28_N_2_O_4_S_2_ [M+H]^+^ 497.1563, found 497.1568.

**4-Methyl-*N*-(2-(7-(4-methylphenylsulfonamido)hept-1-yn-1-yl)phenyl)benzenesulfonamide (1q)**

Oil, yield 80%. ^1^H NMR (400 MHz, CDCl_3_) *δ* 7.78 (d, *J* = 8.4 Hz, 2H), 7.69 (d, *J* = 8.0 Hz, 2H), 7.51 (d, *J* = 8.0 Hz, 1H), 7.33 – 7.27 (m, 4H), 7.25– 7.18 (m, 3H), 6.99 (t, *J* = 7.2 Hz, 1H), 4.99 (t, *J* = 6.0 Hz, 1H), 2.98 (q, *J* = 6.4 Hz, 2H), 2.42 (s, 3H), 2.45 – 2.38 (m, 2H), 2.37 (s, 3H), 1.65 – 1.40 (m, 6H); ^13^C NMR (100 MHz, CDCl_3_) *δ* 144.1, 143.4, 137.6, 137.1, 136.1, 132.0, 129.8, 129.7, 128.9, 127.3, 127.2, 124.3, 119.3, 114.9, 97.5, 75.7, 43.1, 29.3, 28.0, 26.0, 21.6, 21.6, 19.4; HRMS (ESI) calcd for C_27_H_30_N_2_O_4_S_2_ [M+H]^+^ 511.1720, found 511.1723.

**AgSbF_6_-Catalyzed Chemoselective Intramolecular Difunctionalization of Alkynes to Afford 2,3-Fused Indoles**

**General Procedure:**

**Method A**: To a microwave vessel were added DCE, **1** (1 equiv), AgSbF_6_ (10 mol %), and **2** (1.2 equiv) sequentially, and this mixture was irradiated in a microwave oven (80 ºC, 100 W). When the reaction was complete as monitored by TLC, CH_2_Cl_2_ and H_2_O were added to the resulting mixture. After separation of the organic layer, the water layer was extracted with DCM. The combined organic layer was dried over anhydrous Na_2_SO_4_, filtered, evaporated and purified via column chromatography on silica gel (eluent: petroleum ether/ethyl acetate) to afford the desired product.

**Method** **B**: To a microwave vessel were added DCE, **1** (1 equiv), AgSbF_6_ (10 mol %) sequentially, and this mixture was irradiated in a microwave oven (80 ºC, 100 W) for 1–3 h. Then, **2** (2 equiv) was added, and the mixture was reacted under the same condition until the reaction was complete as monitored by TLC, CH_2_Cl_2_ and H_2_O were added to the resulting mixture. After separation of the organic layer, the water layer was extracted with DCM. The combined organic layer was dried over anhydrous Na_2_SO_4_, filtered, evaporated and purified via column chromatography on silica gel (eluent: petroleum ether/ethyl acetate) to afford the desired product.

**4-methyl-*N*-(2-(1-tosyl-1*H*-indol-2-yl)ethyl)benzenesulfonamide (3aa′)**

White solid, Yield 95%, Mp 174–175 ºC. ^1^H NMR (400 MHz, CDCl_3_) *δ* 8.10 (d, *J* = 8.4 Hz, 1H), 7.68 (d, *J* = 8.0 Hz, 2H), 7.54 (d, *J* = 8.0 Hz, 2H), 7.39 (d, *J* = 7.6 Hz, 1H), 7.29 (d, *J* = 8.4 Hz, 1H), 7.23 (d, *J* = 7.6 Hz, 3H), 7.16 (d, *J* = 8.0 Hz, 2H), 6.39 (s, 1H), 4.63 (t, *J* = 6.4 Hz, 1H), 3.38 (dd, *J* = 12.8, 6.4 Hz, 2H), 3.20 (t, *J* = 6.4 Hz, 2H), 2.39 (s, 3H), 2.32 (s, 3H); ^13^C NMR (100 MHz, CDCl_3_) *δ* 145.2, 143.6, 137.4, 137.3, 136.9, 135.6, 130.0, 129.8, 129.6, 127.1, 126.3, 124.6, 124.0, 120.6, 115.0, 111.5, 42.8, 30.0, 21.7, 21.7, HRMS (ESI) calcd for C_13_H_16_N_2_ [M+NH_4_]^+^ 486.1516, found 486.1513.

**1-Phenyl-2,5-ditosyl-2,3,4,5-tetrahydro-1*H*-pyrido[4,3-*b*]indole (3aa)**

White solid, Yield 94% (**Method A**), Mp 85–87 ºC. ^1^H NMR (400 MHz, CDCl_3_) *δ* 8.07 (d, *J* = 8.4 Hz, 1H), 7.59 (d, *J* = 8.4 Hz, 2H), 7.55 (d, *J* = 8.0 Hz, 2H), 7.33 – 7.16 (m, 8H), 7.11 (t, *J* = 7.6 Hz, 1H), 7.05 (d, *J* = 8.0 Hz, 2H), 6.98 (d, *J* = 8.0 Hz, 1H), 6.29 (s, 1H), 3.97 (dd, *J* = 14.8, 6.0 Hz, 1H), 3.28 – 3.14 (m, 1H), 3.07 (dd, *J* = 18.0, 4.0 Hz, 1H), 2.96 – 2.81 (m, 1H), 2.38 (s, 3H), 2.33 (s, 3H); ^13^C NMR (100 MHz, CDCl_3_) *δ* 145.1, 143.5, 138.7, 137.7, 136.2, 135.8, 134.0, 130.1, 129.6, 128.6, 128.3, 128.2, 127.1, 126.5, 124.8, 123.8, 119.1, 116.9, 114.5, 55.1, 38.7, 23.9, 21.8, 21.6; HRMS (ESI) calcd for C_31_H_29_N_2_O_4_S_2_ [M+H]^+^ 557.1563, found 557.1565.

**1-(2-Chlorophenyl)-2,5-ditosyl-2,3,4,5-tetrahydro-1*H*-pyrido[4,3-*b*]indole (3ab)**

White solid, Yield 81% (**Method A**), Mp 195–196 ºC. ^1^H NMR (400 MHz, CDCl_3_) *δ* 8.08 (d, *J* = 8.4 Hz, 1H), 7.64 (t, *J* = 8.8 Hz, 4H), 7.40 (d, *J* = 8.0 Hz, 1H), 7.26 – 7.21 (m, 3H), 7.17 (t, *J* = 7.2 Hz, 1H), 7.11 (d, *J* = 8.0 Hz, 2H), 7.06 (d, *J* = 7.6 Hz, 1H), 7.00 – 6.92 (m, 2H), 6.87 (d, *J* = 7.6 Hz, 1H), 6.72 (s, 1H), 3.88 (dt, *J* = 14.0, 3.6 Hz,1H), 3.49 – 3.37 (m, 1H), 3.30 – 3.22 (m, 2H), 2.36 (s, 3H), 2.35 (s, 3H); ^13^C NMR (100 MHz, CDCl_3_) *δ* 145.2, 143.5, 137.0, 136.5, 136.4, 135.7, 134.4, 133.9, 130.5, 130.4, 130.1, 129.7, 129.5, 127.6, 127.5, 126.8, 126.6, 124.8, 123.9, 118.8, 117.5, 114.6, 52.1, 39.5, 24.4, 21.7, 21.6; HRMS (ESI) calcd for C_31_H_28_ClN_2_O_4_S_2_ [M+H]^+^ 591.1174, found 591.1173.

**1-(3-Chlorophenyl)-2,5-ditosyl-2,3,4,5-tetrahydro-1*H*-pyrido[4,3-*b*]indole (3ac)**

White solid, Yield 91% (**Method A**), Mp 100–102 ºC. ^1^H NMR (400 MHz, CDCl_3_) *δ* 8.08 (d, *J* = 8.4 Hz, 1H), 7.58 (d, *J* = 8.4 Hz, 2H), 7.55 (d, *J* = 8.0 Hz, 2H), 7.29 (d, *J* = 8.4 Hz, 1H), 7.24 – 7.20 (m, 3H), 7.20 – 7.10 (m, 3H), 7.07 (d, *J* = 8.4 Hz, 3H), 6.97 (d, *J* = 8.0 Hz, 1H), 6.23 (s, 1H), 4.01 (dd, *J* = 14.8, 6.4 Hz, 1H), 3.23 – 3.13 (m, 1H), 3.07 (dd, *J* = 18.0, 4.4 Hz, 1H), 2.94 – 2.82 (m, 1H), 2.37 (s, 3H), 2.34 (s, 3H); ^13^C NMR (100 MHz, CDCl_3_) *δ* 145.2, 143.7, 140.8, 137.5, 136.2, 135.7, 134.6, 134.2, 130.2, 129.9, 129.6, 128.6, 128.6, 127.9, 127.0, 126.8, 126.4, 124.9, 124.0, 118.8, 116.2, 114.6, 54.6, 38.8, 23.8, 21.7, 21.6; HRMS (ESI) calcd for C_31_H_28_ClN_2_O_4_S_2_ [M+H]^+^ 591.1174, found 591.1182.

**1-(4-Chlorophenyl)-2,5-ditosyl-2,3,4,5-tetrahydro-1*H*-pyrido[4,3-*b*]indole (3ad)**

White solid, Yield 86% (**Method A**), Mp 103–105 ºC. ^1^H NMR (400 MHz, CDCl_3_) *δ* 8.07 (d, *J* = 8.4 Hz, 1H), 7.58 (d, *J* = 8.4 Hz, 2H), 7.55 (d, *J* = 8.4 Hz, 2H), 7.26 (d, *J* = 6.8 Hz, 1H), 7.22 (d, *J* = 7.2 Hz, 2H), 7.20 (d, *J* = 8.4 Hz, 2H), 7.16 – 7.10 (m, 3H), 7.07 (d, *J* = 8.4 Hz, 2H), 6.95 (d, *J* = 8.0 Hz, 1H), 6.25 (s, 1H), 3.98 (dd, *J* = 14.8, 6.4 Hz, 1H), 3.20 – 3.11 (m, 1H), 3.07 (dd, *J* = 18.0, 4.4 Hz, 1H), 2.96 – 2.80 (m, 1H), 2.38 (s, 3H), 2.34 (s, 3H); ^13^C NMR (100 MHz, CDCl_3_) *δ* 145.2, 143.7, 137.6, 137.3, 136.1, 135.7, 134.3, 134.1, 130.1, 130.0, 129.7, 128.8, 127.9, 127.0, 126.5, 124.9, 123.9, 118.9, 116.3, 114.5, 54.5, 38.7, 23.8, 21.7, 21.6; HRMS (ESI) calcd for C_31_H_28_ClN_2_O_4_S_2_ [M+H]^+^ 591.1174, found 591.1177.

**1-(*p*-tolyl)-2,5-ditosyl-2,3,4,5-tetrahydro-1*H*-pyrido[4,3-*b*]indole (3ae)**

White solid, Yield 86% (**Method A**), Mp 138–140 ºC. ^1^H NMR (400 MHz, CDCl_3_) *δ* 8.10 (d, *J* = 8.4 Hz, 1H), 7.61 (d, *J* = 8.4 Hz, 2H), 7.58 (d, *J* = 8.4 Hz, 2H), 7.32 – 7.26 (m, 1H), 7.24 (d, *J* = 8.4 Hz, 2H), 7.14 (d, *J* = 7.2 Hz, 1H), 7.12 – 7.04 (m, 6H), 7.01 (d, *J* = 7.6 Hz, 1H), 6.29 (s, 1H), 3.99 (dd, *J* = 14.4, 6.0 Hz, 1H), 3.32 – 3.16 (m, 1H), 3.10 (dd, *J* = 18.0, 4.4 Hz, 1H), 2.99 – 2.86 (m, 1H), 2.40 (s, 3H), 2.36 (s, 3H), 2.33 (s, 3H); ^13^C NMR (100 MHz, CDCl_3_) *δ* 145.1, 143.4, 138.1, 137.8, 136.1, 135.8, 135.7, 133.9, 130.1, 129.6, 129.3, 128.5, 128.2, 127.1, 126.5, 124.7, 123.8, 119.1, 117.1, 114.5, 54.8, 38.6, 23.9, 21.7, 21.6, 21.2; HRMS (ESI) calcd for C_32_H_31_N_2_O_4_S_2_ [M+H]^+^ 571.1720, found 571.1727.

**1-(4-(*tert*-butyl)phenyl)-2,5-ditosyl-2,3,4,5-tetrahydro-1*H*-pyrido[4,3-*b*]indole (3af)**

White solid, Yield 92% (**Method A**), Mp 97–99 ºC. ^1^H NMR (400 MHz, CDCl_3_) *δ* 8.11 (d, *J* = 8.4 Hz, 1H), 7.62 (d, *J* = 8.4 Hz, 2H), 7.56 (d, *J* = 8.0 Hz, 2H), 7.29 (d, *J* = 8.4 Hz, 1H), 7.29 – 7.22 (m, 4H), 7.14 (t, *J* = 8.4 Hz, 3H), 7.06 (t, *J* = 8.4 Hz, 3H), 6.29 (s, 1H), 4.02 (dd, *J* = 14.4, 6.0 Hz, 1H), 3.33 – 3.20 (m, 1H), 3.11 (dd, *J* = 17.6, 4.0 Hz, 1H), 3.00 – 2.88 (m, 1H), 2.41 (s, 3H), 2.35 (s, 3H), 1.30 (s, 9H); ^13^C NMR (100 MHz, CDCl_3_) *δ* 151.2, 145.1, 136.2, 135.6, 133.8, 130.1, 129.5, 128.2, 127.0, 126.5, 125.4, 124.7, 123.8, 119.1, 117.2, 114.4, 54.9, 38.7, 34.6, 31.4, 24.0, 21.7, 21.6; HRMS (ESI) calcd for C_35_H_37_N_2_O_4_S_2_ [M+H]^+^ 613.2189, found 613.2188.

**4-(2,5-Ditosyl-2,3,4,5-tetrahydro-1*H*-pyrido[4,3-*b*]indol-1-yl)benzonitrile (3ag)**

White solid, Yield 86% (**Method A**), Mp 92–94 ºC. ^1^H NMR (400 MHz, CDCl_3_) *δ* 8.08 (d, *J* = 8.4 Hz, 1H), 7.59 (d, *J* = 8.0 Hz, 2H), 7.55 (t, *J* = 7.6 Hz, 4H), 7.36 (d, *J* = 7.6 Hz, 2H), 7.29 (t, *J* = 7.6 Hz, 1H), 7.23 (d, *J* = 8.0 Hz, 2H), 7.14 (t, *J* = 7.6 Hz, 1H), 7.08 (d, *J* = 8.0 Hz, 2H), 6.94 (d, *J* = 7.6 Hz, 1H), 6.30 (s, 1H), 4.02 (dd, *J* = 14.8, 5.6 Hz, 1H), 3.19 – 3.02 (m, 2H), 2.93 – 2.76 (m, 1H), 2.39 (s, 3H), 2.35 (s, 3H); ^13^C NMR (100 MHz, CDCl_3_) *δ* 145.3, 144.1, 143.9, 137.3, 136.0, 135.7, 134.3, 132.5, 130.2, 129.8, 129.3, 127.6, 127.0, 126.5, 125.1, 124.0, 118.7, 118.5, 115.2, 114.6, 112.3, 54.6, 38.9, 23.6, 21.8, 21.6; HRMS (ESI) calcd for C_32_H_28_N_3_O_4_S_2_ [M+H]^+^ 582.1516, found 582.1518.

**1-([1,1'-Biphenyl]-4-yl)-2,5-ditosyl-2,3,4,5-tetrahydro-1*H*-pyrido[4,3-*b*]indole (3ah)**

White solid, Yield 87% (**Method A**), Mp 104–106 ºC. ^1^H NMR (400 MHz, CDCl_3_) *δ* 8.09 (d, *J* = 8.4 Hz, 1H), 7.60 (d, *J* = 8.1 Hz, 2H), 7.57 (d, *J* = 8.0 Hz, 2H), 7.52 (d, *J* = 7.6 Hz, 2H), 7.44 (d, *J* = 8.0 Hz, 2H), 7.41 (t, *J* = 7.6 Hz, 2H), 7.34 (d, *J* = 7.2 Hz, 1H), 7.32 – 7.25 (m, 3H), 7.22 (d, *J* = 8.0 Hz, 2H), 7.13 (t, *J* = 7.6 Hz, 1H), 7.06 (d, *J* = 8.4 Hz, 2H), 7.03 (d, *J* = 8.8 Hz, 1H), 6.33 (s, 1H), 4.01 (dd, *J* = 14.4, 6.0 Hz, 1H), 3.38 – 3.16 (m, 1H), 3.10 (dd, *J* = 18.0, 4.0 Hz, 1H), 3.00 – 2.82 (m, 1H), 2.38 (s, 3H), 2.33 (s, 3H); ^13^C NMR (100 MHz, CDCl_3_) *δ* 145.1, 143.5, 141.2, 140.6, 137.7, 136.2, 135.9, 134.0, 130.1, 129.6, 129.0, 128.9, 128.2, 127.6, 127.3, 127.2, 127.1, 126.5, 124.8, 123.8, 119.1, 116.8, 114.5, 54.9, 38.8, 24.0, 21.8, 21.6; HRMS (ESI) calcd for C_37_H_33_N_2_O_4_S_2_ [M+H]^+^ 633.1876, found 633.1886.

**1-(2,4-Dichlorophenyl)-2,5-ditosyl-2,3,4,5-tetrahydro-1H-pyrido[4,3-b]indole (3ai)**

White solid, Yield 92% (**Method A**), Mp 98–100 ºC. ^1^H NMR (400 MHz, CDCl_3_) *δ* 8.08 (d, *J* = 8.4 Hz, 1H), 7.65 (d, *J* = 8.0 Hz, 2H), 7.61 (d, *J* = 8.0 Hz, 2H), 7.41 (d, *J* = 1.2 Hz, 1H), 7.28 – 7.20 (m, 3H), 7.14 (d, *J* = 8.0 Hz, 2H), 7.09 (t, *J* = 7.6 Hz, 1H), 6.98 – 6.90 (m, 2H), 6.80 (d, *J* = 8.4 Hz, 1H), 6.64 (s, 1H), 3.92 – 3.84 (m, 1H), 3.46 – 3.35 (m, 1H), 3.29– 3.21 (m, 2H), (d, *J* = 3.4 Hz, 3H), 2.37 (s, 3H); ^13^C NMR (100 MHz, CDCl_3_) *δ* 145.3, 143.8, 136.8, 136.4, 135.7, 135.2, 135.1, 135.0, 134.0, 131.2, 130.2, 130.2, 129.5, 127.5, 127.4, 127.2, 126.6, 125.0, 124.0, 118.7, 117.0, 114.6, 51.7, 39.7, 24.4, 21.7, 21.6; HRMS (ESI) calcd for C_31_H_27_Cl_2_N_2_O_4_S_2_ [M+H]^+^ 625.0784, found 625.0788.

**1-Propyl-2,5-ditosyl-2,3,4,5-tetrahydro-1*H*-pyrido[4,3-*b*]indole (3aj)**

White powder, Yield 82% (**Method A**), Mp 51–53 ºC. ^1^H NMR (400 MHz, CDCl_3_) *δ* 8.03 (d, *J* = 7.2 Hz, 1H), 7.58 (d, *J* = 8.4 Hz, 2H), 7.52 (d, *J* = 8.4 Hz, 2H), 7.40 – 7.35 (m, 1H), 7.30 – 7.27 (m, 1H), 7.25 – 7.22 (m, 1H), 7.18 (d, *J* = 8.4 Hz, 2H), 7.06 (d, *J* = 8.0 Hz, 2H), 5.14 (dd, *J* = 9.6, 2.8 Hz, 1H), 4.10 (dd, *J* = 14.8, 6.4 Hz, 1H), 3.49 – 3.33 (m, 1H), 2.91 (dd, *J* = 18.0, 4.4 Hz, 1H), 2.72 – 2.60 (m, 1H), 2.35 (s, 3H), 2.33 (s, 3H), 1.91 – 1.76 (m, 1H), 1.73 – 1.47 (m, 3H), 0.97 (t, *J* = 7.2 Hz, 3H); ^13^C NMR (100 MHz, CDCl_3_) *δ* 144.9, 143.4, 138.0, 136.0, 132.1, 130.0, 129.6, 127.7, 127.0, 126.5, 124.6, 123.6, 119.2, 118.4, 114.4, 52.4, 38.4, 37.0, 23.2, 21.7, 21.6, 19.8, 14.0; HRMS (ESI) calcd for C_28_H_30_N_2_O_4_S_2_ [M+H]^+^ 523.1720, found 523.1728.

**8-Fluoro-1-propyl-2,5-ditosyl-2,3,4,5-tetrahydro-1*H*-pyrido[4,3-*b*]indole (3bj)**

White powder, Yield 80% (**Method A**), Mp 140–142 ºC. ^1^H NMR (400 MHz, CDCl_3_) *δ* 7.98 (dd, *J* = 8.8, 4.4 Hz, 1H), 7.60 (d, *J* = 8.4 Hz, 2H), 7.50 (d, *J* = 8.4 Hz, 2H), 7.20 (d, *J* = 8.0 Hz, 2H), 7.09 (d, *J* = 8.0 Hz, 2H), 7.01 (d, *J* = 8.8 Hz, 2H), 5.06 (d, *J* = 7.2 Hz, 1H), 4.09 (dd, *J* = 15.2, 6.4 Hz, 1H), 3.45 – 3.31 (m, 1H), 2.92 (dd, *J* = 18.0, 4.4 Hz, 1H), 2.79 – 2.62 (m, 1H), 2.37 (s, 3H), 2.35 (s, 3H), 1.85 – 1.59 (m, 3H), 1.56 – 1.44 (m, 1H), 0.97 (t, *J* = 7.2 Hz, 3H); ^13^C NMR (101 MHz, CDCl_3_) δ 160.8, 158.4, (145.1, 143.4), 137.7, 135.5, 133.9, 132.1, 130.0, 129.5, (128.6, 128.5), 126.9, 126.3, (119.0, 118.9), (115.4, 115.3), (112.2, 112.0), (104.3, 104.1), 51.9, 38.2, 36.7, 23.3, 21.6, 21.5, 19.6, 13.8; HRMS (ESI) calcd for C_28_H_30_FN_2_O_4_S_2_ [M+H]^+^ 541.1626, found 541.1626.

**8-Chloro-1-propyl-2,5-ditosyl-2,3,4,5-tetrahydro-1*H*-pyrido[4,3-*b*]indole (3cj)**

White powder, Yield 83% (**Method A**), Mp 150–152 ºC. ^1^H NMR (400 MHz, CDCl_3_) *δ* 7.96 (d, *J* = 8.8 Hz, 1H), 7.59 (d, *J* = 8.4 Hz, 2H), 7.50 (d, *J* = 8.0 Hz, 2H), 7.32 (d, *J* = 2.0 Hz, 1H), 7.24 (d, *J* = 2.0 Hz, 1H), 7.20 (d, *J* = 8.8 Hz, 2H), 7.09 (d, *J* = 8.0 Hz, 2H), 5.06 (dd, *J* = 10.0, 2.4 Hz, 1H), 4.10 (dd, *J* = 14.8, 6.4 Hz, 1H), 3.41 – 3.29 (m, 1H), 2.91 (dd, *J* = 18.0, 4.4 Hz, 1H), 2.77 – 2.63 (m, 1H), 2.37 (s, 3H), 2.35 (s, 3H), 1.79 – 1.58 (m, 3H), 1.54 – 1.47 (m, 1H), 0.97 (t, *J* = 7.2 Hz, 3H); ^13^C NMR (100 MHz, CDCl_3_) *δ* 145.2, 143.4, 137.6, 135.5, 134.2, 133.6, 130.1, 129.5, 129.3, 128.8, 127.0, 126.4, 124.6, 118.6, 118.0, 115.4, 51.9, 38.1, 36.8, 23.2, 21.7, 21.5, 19.7, 13.8; HRMS (ESI) calcd for C_28_H_30_ClN_2_O_4_S_2_ [M+H]^+^ 557.1330, found 557.1335.

**7-Chloro-1-propyl-2,5-ditosyl-2,3,4,5-tetrahydro-1*H*-pyrido[4,3-*b*]indole (3dj)**

White powder, Yield 82% (**Method A**), Mp 153–155 ºC. ^1^H NMR (400 MHz, CDCl_3_) *δ* 8.07 (s, 1H), 7.57 (d, *J* = 8.0 Hz, 2H), 7.54 (d, *J* = 8.4 Hz, 2H), 7.30 – 7.20 (m, 4H), 7.07 (d, *J* = 8.0 Hz, 2H), 5.10 (d, *J* = 7.6 Hz, 1H), 4.09 (dd, *J* = 14.8, 6.4 Hz, 1H), 3.43 – 3.28 (m, 1H), 2.87 (dd, *J* = 18.0, 4.4 Hz, 1H), 2.72 – 2.58 (m, 1H), 2.38 (s, 3H), 2.34 (s, 3H), 1.80 – 1.59 (m, 3H), 1.55 – 1.47 (m, 1H), 0.96 (t, *J* = 7.2 Hz, 3H); ^13^C NMR (100 MHz, CDCl_3_) *δ* 145.2, 143.4, 137.6, 136.1, 135.5, 132.6, 130.4, 130.1, 129.5, 126.9, 126.4, 126.0, 124.1, 119.0, 118.7, 114.5, 52.0, 38.1, 36.9, 23.0, 21.7, 21.6, 19.7, 13.8; HRMS (ESI) calcd for C_28_H_30_ClN_2_O_4_S_2_ [M+H]^+^ 557.1330, found 557.1335.

**8-Bromo-1-propyl-2,5-ditosyl-2,3,4,5-tetrahydro-1*H*-pyrido[4,3-*b*]indole (3ej)**

White powder, Yield 80% (**Method A**), Mp 151–153 ºC. ^1^H NMR (400 MHz, CDCl_3_) *δ* 7.91 (d, *J* = 9.2 Hz, 1H), 7.59 (d, *J* = 8.0 Hz, 2H), 7.50 (d, *J* = 8.0 Hz, 2H), 7.47 (d, *J* = 1.6 Hz, 1H), 7.37 (dd, *J* = 9.2, 1.6 Hz, 1H), 7.20 (d, *J* = 8.4 Hz, 2H), 7.09 (d, *J* = 8.0 Hz, 2H), 5.06 (dd, *J* = 10.0, 2.4 Hz, 1H), 4.10 (dd, *J* = 15.2, 6.4 Hz, 1H), 3.44 – 3.32 (m, 1H), 2.91 (dd, *J* = 18.0, 4.4 Hz, 1H), 2.76 – 2.64 (m, 1H), 2.37 (s, 3H), 2.35 (s, 3H), 1.81 – 1.58 (m, 3H), 1.53 – 1.47 (m, 1H), 0.97 (t, *J* = 7.2 Hz, 3H); ^13^C NMR (100 MHz, CDCl_3_) *δ* 145.2, 143.4, 137.7, 135.5, 134.6, 133.5, 130.1, 129.5, 129.3, 127.3, 127.0, 126.4, 121.0, 118.4, 117.0, 115.7, 51.9, 38.1, 36.8, 23.2, 21.6, 21.5, 19.7, 13.8; HRMS (ESI) calcd for C_28_H_30_BrN_2_O_4_S_2_ [M+H]^+^ 601.0825, found 601.0826.

**8-Methyl-1-propyl-2,5-ditosyl-2,3,4,5-tetrahydro-1*H*-pyrido[4,3-*b*]indole (3fj)**

White powder, Yield 87% (**Method A**), Mp 155–157 ºC. ^1^H NMR (400 MHz, CDCl_3_) *δ* 7.89 (d, *J* = 8.8 Hz, 1H), 7.58 (d, *J* = 8.0 Hz, 2H), 7.50 (d, *J* = 8.4 Hz, 2H), 7.17 (d, *J* = 8.4 Hz, 2H), 7.14 (s, 1H), 7.09 (d, *J* = 8.0 Hz, 1H), 7.07 (d, *J* = 7.6 Hz, 2H), 5.11 (d, *J* = 7.6 Hz, 1H), 4.08 (dd, *J* = 14.8, 6.0 Hz, 1H), 3.46 – 3.31 (m, 1H), 2.88 (dd, *J* = 18.0, 4.8 Hz, 1H), 2.67– 2.56 (m, 1H), 2.43 (s, 3H), 2.35 (s, 3H), 2.34 (s, 3H), 1.88 – 1.60 (m, 3H), 1.56 – 1.48 (m, 1H), 0.98 (t, *J* = 7.2 Hz, 3H); ^13^C NMR (100 MHz, CDCl_3_) *δ* 144.7, 143.3, 137.9, 135.8, 134.1, 133.2, 131.9, 129.9, 129.5, 127.8, 126.9, 126.3, 125.8, 119.0, 118.3, 114.0, 52.3, 38.3, 36.8, 23.1, 21.6, 21.5, 21.4, 19.7, 13.9; HRMS (ESI) calcd for C_29_H_33_N_2_O_4_S_2_ [M+H]^+^ 537.1876, found 537.1877.

**8-Methoxy-1-propyl-2,5-ditosyl-2,3,4,5-tetrahydro-1*H*-pyrido[4,3-*b*]indole (3gj)**

White powder, Yield 92% (**Method A**), Mp 166–168 ºC. ^1^H NMR (400 MHz, CDCl_3_) *δ* 7.92 (d, *J* = 9.2 Hz, 1H), 7.58 (d, *J* = 8.0 Hz, 2H), 7.48 (d, *J* = 8.4 Hz, 2H), 7.17 (d, *J* = 8.0 Hz, 2H), 7.08 (d, *J* = 7.6 Hz, 2H), 6.87 (dd, *J* = 9.2, 2.4 Hz, 1H), 6.79 (d, *J* = 2.0 Hz, 1H), 5.10 (d, *J* = 7.2 Hz, 1H), 4.08 (dd, *J* = 15.2, 6.4 Hz, 1H), 3.85 (s, 3H), 3.46 – 3.31 (m, 1H), 2.88 (dd, *J* = 18.0, 4.4 Hz, 1H), 2.70 – 2.56 (m, 1H), 2.35 (s, 3H), 2.34 (s, 3H), 1.86 – 1.58 (m, 3H), 1.55 – 1.49 (m, 1H), 0.97 (t, *J* = 7.2 Hz, 3H); ^13^C NMR (100 MHz, CDCl_3_) *δ* 156.4, 144.7, 143.3, 137.8, 135.7, 132.7, 130.4, 129.9, 129.5, 128.6, 126.9, 126.3, 119.2, 115.2, 112.4, 101.6, 55.7, 52.2, 38.3, 36.7, 23.2, 21.6, 21.6, 19.7, 13.9; HRMS (ESI) calcd for C_29_H_33_N_2_O_5_S_2_ [M+H]^+^ 553.1825, found 553.1834.

**1-Propyl-2,5-ditosyl-2,3,4,5-tetrahydro-1*H*-pyrido[4,3-*b*]indole-8-carbonitrile (3hj)**

White powder, Yield 76% (**Method A**), Mp 162–164 ºC. ^1^H NMR (400 MHz, CDCl_3_) *δ* 8.15 (d, *J* = 8.8 Hz, 1H), 7.69 (s, 1H), 7.61 (d, *J* = 8.0 Hz, 2H), 7.56 (d, *J* = 8.4 Hz, 2H), 7.54 (d, *J* = 8.8 Hz, 1H),7.25 (d, *J* = 8.4 Hz, 2H), 7.11 (d, *J* = 8.0 Hz, 2H), 5.09 (d, *J* = 7.2 Hz, 1H), 4.12 (dd, *J* = 14.8, 6.4 Hz, 1H), 3.46 – 3.29 (m, 1H), 2.96 (dd, *J* = 18.0, 4.4 Hz, 1H), 2.87 – 2.74 (m, 1H), 2.39 (s, 3H), 2.35 (s, 3H), 1.79 – 1.60 (m, 3H), 1.54 – 1.40 (m, 1H), 0.97 (t, *J* = 7.2 Hz, 3H); ^13^C NMR (100 MHz, CDCl_3_) *δ* 145.7, 143.6, 137.5, 137.4, 135.2, 134.8, 130.2, 129.6, 127.6, 127.5, 127.0, 126.5, 123.0, 119.2, 118.5, 115.0, 107.0, 51.7, 38.0, 36.9, 23.2, 21.7, 21.5, 19.7, 13.8; HRMS (ESI) calcd for C_29_H_30_N_3_O_4_S_2_ [M+H]^+^ 548.1672, found 548.1677.

**1-Propyl-2,5-ditosyl-8-(trifluoromethyl)-2,3,4,5-tetrahydro-1*H*-pyrido[4,3-*b*]indole (3ij)**

White powder, Yield 74% (**Method A**), Mp 58–60 ºC. ^1^H NMR (400 MHz, CDCl_3_) *δ* 8.14 (d, *J* = 8.4 Hz, 1H), 7.64 – 7.49 (m, 6H), 7.23 (d, *J* = 7.6 Hz, 2H), 7.09 (d, *J* = 7.2 Hz, 2H), 5.13 (d, *J* = 10.0 Hz, 1H), 4.13 (dd, *J* = 15.2, 6.0 Hz, 1H), 3.46 – 3.34 (m, 1H), 2.95 (d, *J* = 18.0 Hz, 1H), 2.86 – 2.64 (m, 1H), 2.38 (s, 3H), 2.34 (s, 3H), 1.86 – 1.60 (m, 3H), 1.56 – 1.45 (m, 1H), 0.98 (t, *J* = 7.2 Hz, 3H); ^13^C NMR (100 MHz, CDCl_3_) *δ* 145.4, 143.5, 137.6, 135.4, 134.0, 130.1, 129.7 (q, *J_C-F_* = 8.2 Hz), 129.5, 128.3 (q, *J_C-F_* = 4.7 Hz), 127.2, 127.0, 126.4, 124.4 (q, *J_C-F_* = 264.6 Hz), 121.2 (q, *J_C-F_* = 2.8 Hz), 119.0, 115.6 (q, *J_C-F_* = 4.1 Hz), 114.5, 51.9, 38.1, 36.9, 23.2, 21.7, 21.5, 19.6, 13.8; HRMS (ESI) calcd for C_29_H_30_F_3_N_2_O_4_S_2_ [M+H]^+^ 591.1594, found 591.1596.

**8-Nitro-1-propyl-2,5-ditosyl-2,3,4,5-tetrahydro-1*H*-pyrido[4,3-*b*]indole (8jj)**

White powder, Yield 72% (**Method A**), Mp 87–89 ºC. ^1^H NMR (400 MHz, CDCl_3_) *δ* 8.27 (s, 1H), 8.17 (s, 2H), 7.62 (d, *J* = 8.0 Hz, 2H), 7.58 (d, *J* = 8.0 Hz, 2H), 7.25 (d, *J* = 8.0 Hz, 2H), 7.11 (d, *J* = 8.0 Hz, 2H), 5.13 (d, *J* = 9.6 Hz, 1H), 4.14 (dd, *J* = 15.2, 6.0 Hz, 1H), 3.45 – 3.33 (m, 1H), 2.98 (dd, *J* = 17.6, 4.0 Hz, 1H), 2.90 – 2.76 (m, 1H), 2.39 (s, 3H), 2.35 (s, 3H), 1.86 – 1.59 (m, 3H), 1.53 – 1.43 (m, 1H), 0.97 (t, *J* = 7.2 Hz, 3H); ^13^C NMR (100 MHz, CDCl_3_) *δ* 145.9, 144.1, 143.6, 138.7, 137.4, 135.6, 135.1, 130.3, 129.6, 127.4, 127.1, 126.5, 119.6, 119.4, 114.4, 114.4, 51.7, 38.0, 37.0, 23.3, 21.7, 21.6, 19.7, 13.8; HRMS (ESI) calcd for C_28_H_30_N_3_O_6_S_2_ [M+H]^+^ 568.1571, found 568.1571.

**Methyl 1-propyl-2,5-ditosyl-2,3,4,5-tetrahydro-1*H*-pyrido[4,3-*b*]indole-8-carboxylate (3kj)**

White powder, Yield 79% (**Method A**)，Mp 79–81 ºC. ^1^H NMR (400 MHz, CDCl_3_) *δ* 8.08 (d, *J* = 3.6 Hz, 1H), 8.07 (d, *J* = 3.6 Hz, 1H), 7.97 (dd, *J* = 8.8, 1.2 Hz, 1H), 7.59 (d, *J* = 8.4 Hz, 2H), 7.54 (d, *J* = 8.4 Hz, 2H), 7.21 (d, *J* = 8.0 Hz, 2H), 7.06 (d, *J* = 8.0 Hz, 2H), 5.15 (dd, *J* = 9.6, 3.2 Hz, 1H), 4.13 (dd, *J* = 14.8, 6.0 Hz, 1H), 3.96 (s, 3H), 3.49 – 3.33 (m, 1H), 2.92 (dd, *J* = 17.6, 4.4 Hz, 1H), 2.81 – 2.64 (m, 1H), 2.37 (s, 3H), 2.33 (s, 3H), 1.91 – 1.60 (m, 3H), 1.56 – 1.47 (m, 1H), 0.98 (t, *J* = 7.2 Hz, 2H); ^13^C NMR (100 MHz, CDCl_3_) *δ* 167.1, 145.3, 143.4, 138.4, 137.6, 135.5, 133.5, 130.1, 129.47, 127.4, 126.9, 126.4, 125.7, 125.5, 120.3, 119.3, 113.9, 52.3, 52.0, 38.2, 36.9, 23.1, 21.6, 21.5, 19.7, 13.8; HRMS (ESI) calcd for C_30_H_33_N_2_O_6_S_2_ [M+H]^+^ 581.1775, found 581.1779.

**2,5-Ditosyl-2,3,4,5-tetrahydro-1*H*-pyrido[4,3-*b*]indole (3ak)**

White powder, Yield 98% (**Method B:** After the mixture was reacted for 3 h, 40% formaldehyde (2equiv) and trifluoroacetic (1.5 equiv) were added), Mp 159–161 ºC. ^1^H NMR (400 MHz, CDCl_3_) *δ* 8.12 (d, *J* = 8.0 Hz, 1H), 7.74 (d, *J* = 7.6 Hz, 2H), 7.62 (d, *J* = 7.6 Hz, 2H), 7.34 (d, *J* = 7.6 Hz, 2H), 7.31 – 7.27 (m, 2H), 7.23 (d, *J* = 7.6Hz, 1H), 7.20 (dd, *J* = 7.6 Hz, 2H),4.20 (s, 2H), 3.42 (t, *J* = 5.6 Hz, 2H), 3.22 (t, *J* = 5.6 Hz, 2H), 2.43 (s, 3H), 2.35 (s, 3H); ^13^C NMR (100 MHz, CDCl_3_) *δ* 145.1, 143.9, 136.3, 135.6, 133.3, 132.4, 130.0, 129.9, 127.7, 127.5, 126.4, 124.7, 123.7, 117.9, 114.5, 114.5, 43.6, 42.6, 29.7, 25.3, 21.5; HRMS (ESI) calcd for C_25_H_24_N_2_O_4_S_2_ [M+H]^+^ 481.1250, found 481.1247.

**1-Cyclohexyl-2,5-ditosyl-2,3,4,5-tetrahydro-1*H*-pyrido[4,3-*b*]indole (3al)**

White powder, Yield 74% (**Method A**), Mp 53–55 ºC. ^1^H NMR (400 MHz, CDCl_3_) *δ* 7.99 (d, *J* = 8.8 Hz, 1H), 7.53 (d, *J* = 8.0 Hz, 2H), 7.41 (d, *J* = 8.4 Hz, 2H), 7.38 (d, *J* = 2.4 Hz, 1H),7.26 – 7.23 (m, 2H), 7.19 (d, *J* = 8.0 Hz, 2H), 6.83 (d, *J* = 8.0 Hz, 2H), 4.81 (d, *J* = 8.0 Hz, 1H), 4.15 (dd, *J* = 15.2, 7.2 Hz, 1H), 3.57 – 3.46 (m, 1H), 2.83 (dd, *J* = 18.4, 5.6 Hz, 1H), 2.71 – 2.59 (m, 1H), 2.35 (s, 3H), 2.22 (s, 3H), 1.72 – 1.59 (m, 4H), 1.32 – 1.27 (m, 2H), 1.19 – 1.10 (m, 3H); ^13^C NMR (100 MHz, CDCl_3_) *δ* 144.8, 143.2, 137.2, 135.8, 135.7, 132.2, 129.9, 129.2, 129.0, 126.6, 126.4, 124.2, 123.4, 119.3, 118.7, 114.2, 57.2, 43.5, 38.9, 31.1, 30.1, 26.5, 26.3, 26.2, 22.7, 21.6, 21.4; HRMS (ESI) calcd for C_31_H_34_N_2_O_4_S_2_ [M+H]^+^ 563.2033, found 563.2042.

**1-(Prop-1-en-1-yl)-2,5-ditosyl-2,3,4,5-tetrahydro-1*H*-pyrido[4,3-*b*]indole** **(3am)**

White powder, Yield 65% (**Method A**), Mp 56–58 ºC. ^1^H NMR (400 MHz, CDCl_3_) *δ* 8.08 (d, *J* = 8.8 Hz, 1H), 7.63 (d, *J* = 8.4 Hz, 2H), 7.58 (d, *J* = 8.4 Hz, 2H), 7.31 – 7.26 (m, 2H), 7.23 – 7.14 (m, 5H), 5.56 (d, *J* = 4.4 Hz, 1H), 5.48 (t, *J* = 4.4 Hz, 2H), 4.06 (dd, *J* = 14.0, 6.0 Hz, 1H), 3.30 – 3.21 (m, 1H), 3.11 (dd, *J* = 17.6, 4.0 Hz, 1H), 2.96 – 2.83 (m, 1H), 2.38 (s, 3H), 2.36 (s, 3H), 1.57 (d, *J* = 4.4 Hz, 3H); ^13^C NMR (100 MHz, CDCl_3_) *δ* 144.9, 143.2, 137.9, 136.0, 135.7, 133.0, 130.4, 130.0, 129.5, 127.9, 127.4, 127.1, 126.4, 124.5, 123.5, 118.9, 117.1, 114.3, 53.6, 38.7, 24.6, 21.6, 21.5, 17.6; HRMS (ESI) calcd for C_28_H_28_N_2_O_4_S_2_ [M+H]^+^ 521.1563, found 521.1567.

**1-Benzyl-2,5-ditosyl-2,3,4,5-tetrahydro-1*H*-pyrido[4,3-*b*]indole (3an)**

White powder, Yield 78% (**Method A**), Mp 70–72 ºC. ^1^H NMR (400 MHz, CDCl_3_) *δ* 8.09 (d, *J* = 8.4 Hz, 1H), 7.54 (d, *J* = 8.4 Hz, 2H), 7.39 (d, *J* = 8.0 Hz, 2H), 7.32 – 7.26 (m, 1H), 7.25 – 7.17 (m, 5H), 7.14 (t, *J* = 7.6 Hz, 2H), 7.06 (d, *J* = 8.4 Hz, 2H), 6.97 (d, *J* = 7.2 Hz, 2H), 5.44 (t, *J* = 6.0 Hz, 1H), 3.89 (dd, *J* = 14.4, 6.0 Hz, 1H), 3.26 (dd, *J* = 13.6, 5.2 Hz, 1H), 3.10 (dd, *J* = 13.6, 6.8 Hz, 1H), 3.05 – 2.98 (m, 1H), 2.98 – 2.90 (m, 1H), 2.88 – 2.76 (m, 1H), 2.37 (s, 3H), 2.34 (s, 3H); ^13^C NMR (100 MHz, CDCl_3_) *δ* 144.9, 143.2, 137.4, 137.1, 136.0, 135.7, 133.1, 130.0, 129.8, 129.5, 128.4, 127.7, 127.0, 126.7, 126.4, 124.5, 123.6, 118.6, 118.1, 114.5, 53.2, 41.2, 38.8, 23.7, 21.6, 21.5; HRMS (ESI) calcd for C_32_H_30_N_2_O_4_S_2_ [M+H]^+^ 571.1720, found 571.1732.

**(*E*)-1-Styryl-2,5-ditosyl-2,3,4,5-tetrahydro-1*H*-pyrido[4,3-*b*]indole (3ao)**

White solid, Yield 91% (**Method A**), Mp 96–98 ºC. ^1^H NMR (400 MHz, CDCl_3_) *δ* 8.11 (d, *J* = 8.0 Hz, 1H), 7.65 (d, *J* = 8.0 Hz, 2H), 7.61 (d, *J* = 8.0 Hz, 2H), 7.33 – 7.17 (m, 8H), 7.16 – 7.09 (m, 4H), 6.30 (d, *J* = 16.0 Hz, 1H), 6.13 (dd, *J* = 16.0, 6.0 Hz, 1H), 5.78 (d, *J* = 6.0 Hz, 1H), 4.13 (dd, *J* = 13.6, 6.0 Hz, 1H), 3.37 – 3.26 (m, 1H), 3.17 (dd, *J* = 17.6, 3.2 Hz, 1H), 3.01 – 2.89 (m, 1H), 2.37 (s, 3H), 2.33 (s, 3H); ^13^C NMR (100 MHz, CDCl_3_) *δ* 145.2, 143.5, 137.8, 136.2, 136.0, 135.7, 134.0, 133.4, 130.2, 129.7, 128.6, 128.2, 127.9, 127.2, 126.8, 126.5, 125.4, 124.8, 123.8, 119.0, 116.6, 114.5, 53.9, 39.0, 24.8, 21.8, 21.6; HRMS (ESI) calcd for C_33_H_31_N_2_O_4_S_2_ [M+H]^+^ 583.1720, found 583.1728.

**1-(Naphthalen-2-yl)-2,5-ditosyl-2,3,4,5-tetrahydro-1*H*-pyrido[4,3-*b*]indole (3ap)**

White solid, Yield 81% (**Method A**), Mp 175–176 ºC. ^1^H NMR (400 MHz, CDCl_3_) *δ* 8.11 (d, *J* = 8.4 Hz, 1H), 7.78 (d, *J* = 7.6 Hz, 1H), 7.75 (d, *J* = 8.8 Hz, 1H), 7.62 (d, *J* = 8.4 Hz, 2H), 7.56 (d, *J* = 8.4 Hz, 2H), 7.54 – 7.50 (m, 2H), 7.48 – 7.38 (m, 2H), 7.35 (s, 1H), 7.29 – 7.21 (m, 3H), 7.07 (t, *J* = 7.2 Hz, 1H), 7.00 (d, *J* = 7.6 Hz, 2H), 6.99 (t, *J* = 7.2 Hz, 1H), 6.44 (s, 1H), 3.99 (dd, *J* = 14.4, 6.0 Hz, 1H), 3.35 – 3.18 (m, 1H), 3.11 (dd, *J* = 18.0, 4.4 Hz, 1H), 3.00 – 2.87 (m, 1H), 2.39 (s, 3H), 2.29 (s, 3H); ^13^C NMR (100 MHz, CDCl_3_) *δ* 145.1, 143.5, 137.7, 136.2, 136.1, 135.8, 134.1, 133.2, 133.0, 130.1, 129.5, 128.6, 128.3, 128.2, 127.7, 127.6, 127.0, 126.5, 126.5, 126.4, 126.3, 124.8, 123.9, 119.1, 116.9, 114.6, 55.2, 38.7, 24.0, 21.7, 21.5; HRMS (ESI) calcd for C_35_H_31_N_2_O_4_S_2_ [M+H]^+^ 607.1720, found 607.1711.

**1-(Thiophen-2-yl)-2,5-ditosyl-2,3,4,5-tetrahydro-1*H*-pyrido[4,3-*b*]indole (3aq)**

White solid, Yield 64% (**Method A**), Mp 142–144 ºC. ^1^H NMR (400 MHz, CDCl_3_) *δ* 8.08 (d, *J* = 8.4 Hz, 1H), 7.58 (t, *J* = 8.0 Hz, 4H), 7.29 – 7.23 (m, 1H), 7.21 (d, *J* = 8.4 Hz, 2H), 7.19 – 7.16 (m, 1H), 7.15 – 7.08 (m, 4H), 6.85 (d, *J* = 3.2 Hz, 2H), 6.50 (s, 1H), 4.01 (dd, *J* = 14.4, 6.0 Hz, 1H), 3.43 – 3.28 (m, 1H), 3.17 (dd, *J* = 18.0, 4.0 Hz, 1H), 3.05 – 2.89 (m, 1H), 2.35 (s, 6H); ^13^C NMR (100 MHz, CDCl_3_) *δ* 145.2, 143.5, 142.7, 137.5, 136.2, 135.8, 133.6, 130.1, 129.6, 127.8, 127.7, 127.1, 126.6, 126.5, 126.4, 124.8, 123.8, 119.0, 117.5, 114.5, 51.0, 38.8, 24.4, 21.7, 21.6; HRMS (ESI) calcd for C_29_H_27_N_2_O_5_S_3_ [M+H]^+^ 563.1127, found 563.1122.

**1-(Furan-2-yl)-2,5-ditosyl-2,3,4,5-tetrahydro-1*H*-pyrido[4,3-*b*]indole (3ar)**

White solid, Yield 36% (**Method A**), Mp 162–163 ºC. ^1^H NMR (400 MHz, CDCl_3_) *δ* 8.09 (d, *J* = 8.4 Hz, 1H), 7.61 (d, *J* = 8.4 Hz, 2H), 7.59 (d, *J* = 8.4 Hz, 2H), 7.29 – 7.19 (m, 4H), 7.16 – 7.12 (m, 4H), 6.29 (s, 1H), 6.19 (dd, *J* = 2.8, 2.0 Hz, 1H), 6.03 (d, *J* = 3.2 Hz, 1H), 4.02 (dd, *J* = 14.0, 6.0 Hz, 1H), 3.41 – 3.31 (m, 1H), 3.24 (dd, *J* = 17.6, 3.6 Hz, 1H), 3.07– 2.96 (m, 1H), 2.37 (s, 3H), 2.36 (s, 3H); ^13^C NMR (100 MHz, CDCl_3_) *δ* 151.6, 145.2, 143.4, 143.0, 137.5, 136.2, 135.8, 134.0, 130.2, 129.6, 127.7, 127.2, 126.6, 124.7, 123.8, 118.8, 115.4, 114.5, 110.2, 109.8, 49.4, 39.6, 24.8, 21.7, 21.6; HRMS (ESI) calcd for C_29_H_27_N_2_O_5_S_2_ [M+H]^+^ 547.1356, found 547.1354.

**1-(Benzo[*d*][1,3]dioxol-5-yl)-2,5-ditosyl-2,3,4,5-tetrahydro-1*H*-pyrido[4,3-*b*]indole (3as)**

White solid, Yield 87% (**Method A**), Mp 92–94 ºC. ^1^H NMR (400 MHz, CDCl_3_) *δ* 8.07 (d, *J* = 8.4 Hz, 1H), 7.57 (d, *J* = 8.8 Hz, 2H), 7.55 (d, *J* = 8.8 Hz, 2H), 7.26 (t, *J* = 7.6 Hz, 1H), 7.21 (d, *J* = 8.0 Hz, 2H), 7.13 (t, *J* = 7.6 Hz, 1H), 7.07 (d, *J* = 8.0 Hz, 2H), 7.00 (d, *J* = 7.6 Hz, 1H), 6.69 (s, 1H), 6.63 (s, 2H), 6.19 (s, 1H), 5.91 (d, *J* = 4.4 Hz, 2H), 3.97 (dd, *J* = 14.8, 6.0 Hz, 1H), 3.29 – 3.16 (m, 1H), 3.06 (dd, *J* = 18.0, 4.0 Hz, 1H), 2.94 – 2.79 (m, 1H), 2.37 (s, 3H), 2.34 (s, 3H); ^13^C NMR (100 MHz, CDCl_3_) *δ* 148.0, 147.7, 145.2, 143.5, 137.9, 136.3, 135.9, 134.0, 132.8, 130.1, 129.6, 128.2, 127.1, 126.5, 124.8, 123.9, 122.4, 119.1, 117.1, 114.6, 109.1, 108.1, 101.3, 55.0, 38.6, 24.0, 21.7, 21.6; HRMS (ESI) calcd for C_32_H_29_N_2_O_6_S_2_ [M+H]^+^ 601.1462, found 601.1468.

**2-(Methylsulfonyl)-1-propyl-5-tosyl-2,3,4,5-tetrahydro-1*H*-pyrido[4,3-*b*]indole (3lj)**

White solid, Yield 72% (**Method A**), Mp 141–143 ºC. ^1^H NMR (400 MHz, CDCl_3_) *δ* 8.21 (d, *J* = 8.4 Hz, 1H), 7.63 (d, *J* = 7.6 Hz, 2H), 7.41 – 7.32 (m, 2H), 7.29 (d, *J* = 7.6 Hz, 1H), 7.20 (d, *J* = 7.6 Hz, 2H), 4.88 (d, *J* = 9.2 Hz, 1H), 4.15 (dd, *J* = 15.2, 5.2 Hz, 1H), 3.51 – 3.34 (m, 1H), 3.24 – 3.02 (m, 2H), 2.55 (s, 3H), 2.34 (s, 3H), 1.90 – 1.64 (m, 2H), 1.62 – 1.47 (m, 2H), 0.99 (t, *J* = 6.8 Hz, 3H); ^13^C NMR (101 MHz, CDCl_3_) δ 145.2, 136.3, 135.8, 131.6, 130.0, 127.5, 126.3, 124.9, 123.8, 119.4, 118.4, 114.7, 52.1, 39.7, 38.2, 36.4, 23.7, 21.6, 19.7, 13.8; HRMS (ESI) calcd for C_22_H_27_N_2_O_4_S_2_ [M+NH_4_]^+^ 464.1672, found 464.1677.

**1-Propyl-5-tosyl-1,3,4,5-tetrahydropyrano[4,3-*b*]indole (3mj)**

Oil, Yield 64% (**Method A**). ^1^H NMR (400 MHz, CDCl_3_) *δ* 8.16 (d, *J* = 8.0 Hz, 1H), 7.64 (d, *J* = 8.0 Hz, 2H), 7.34 (d, *J* = 8.0 Hz, 1H), 7.29 (d, *J* = 7.6 Hz, 1H), 7.22 (d, *J* = 7.6 Hz, 1H), 7.18 (d, *J* = 8.4 Hz, 2H), 4.84 (dd, *J* = 8.4, 2.0 Hz, 1H), 4.16 (dt, *J* = 11.6, 4.4 Hz, 1H), 3.79 – 3.70 (m, 1H), 3.18 – 3.08 (m, 2H), 2.33 (s, 3H), 1.99 – 1.90 (m, 1H), 1.83 – 1.69 (m, 1H), 1.49 – 1.34 (m, 2H); ^13^C NMR (100 MHz, CDCl_3_) *δ* 144.8, 136.3, 135.7, 133.0, 129.8, 127.7, 126.4, 124.1, 123.4, 120.3, 118.8, 114.7, 72.8, 62.2, 36.3, 26.1, 21.6, 18.2, 14.1; HRMS (ESI) calcd for C_21_H_24_NO_3_S [M+H]^+^ 370.1471, found 370.1467.

**(2-Hydroxyphenyl)(2-propyl-1-tosylpyrrolidin-3-yl)methanone (3nj’)**

White solid, Yield 85% (**Method A**), Mp 136–138 ºC. ^1^H NMR (400 MHz, CDCl_3_) *δ* 11.66 (s, 1H), 7.65 (d, *J* = 8.0 Hz, 2H), 7.60 (d, *J* = 8.0 Hz, 1H), 7.48 (t, *J* = 7.2 Hz, 1H), 7.23 (d, *J* = 8.0 Hz, 2H), 6.95 (d, *J* = 8.3 Hz, 1H), 6.89 (t, *J* = 8.0 Hz, 1H), 4.15 – 4.07 (m, 1H), 3.67 – 3.61 (m, 1H), 3.60 – 3.52 (m, 1H), 3.43 – 3.33 (m, 1H), 2.44 (s, 3H), 2.21 – 2.10 (m, 1H), 2.02 – 1.68 (m, 3H), 1.52 – 1.40 (m, 2H), 0.99 (t, *J* = 7.3 Hz, 3H); ^13^C NMR (100 MHz, CDCl_3_) *δ* 204.6, 163.1, 143.7, 136.7, 134.2, 129.5, 129.4, 127.6, 119.0, 118.9, 117.7, 62.8, 50.5, 48.4, 39.6, 28.4, 21.6, 19.4, 14.1; HRMS (ESI) calcd for C_21_H_26_NO_4_S [M+H]^+^ 388.1577, found 388.1581.

**1-Phenyl-2,6-ditosyl-1,2,3,4,5,6-hexahydroazepino[4,3-*b*]indole (3oa)**

White solid, Yield (1) 88% (**Method B:** After the mixture was reacted for 3 h, **2a** (2equiv) was added), Yield (2) 70% (**Method A**), Mp 79–81 ºC. ^1^H NMR (400 MHz, CDCl_3_) *δ* 8.18 (d, *J* = 8.4 Hz, 1H), 7.58 (d, *J* = 8.4 Hz, 2H), 7.44 (d, *J* = 8.0 Hz, 2H), 7.37 – 7.28 (m, 1H), 7.25 – 7.02 (m, 7H), 7.06 – 7.02 (m, 2H), 7.00 (d, *J* = 8.0 Hz, 2H), 6.68 (s, 1H), 3.84– 3.72 (m, 1H), 3.20 (dt, *J* = 14.0, 4.4 Hz, 1H), 3.04 (dt, *J* = 17.6, 4.8 Hz, 1H), 2.79 – 2.64 (m, 1H), 2.39 (s, 3H), 2.35 (s, 3H), 1.66 – 1.56 (m, 2H); ^13^C NMR (100 MHz, CDCl_3_) *δ* 145.0, 143.5, 139.0, 137.9, 137.3, 136.3, 136.3, 130.1, 129.9, 129.4, 128.8, 127.8, 127.7, 127.0, 126.4, 124.7, 124.1, 120.7, 118.1, 115.2, 55.1, 45.2, 26.1, 25.3, 21.8, 21.6; HRMS (ESI) calcd for C_32_H_30_N_2_O_4_S_2_ [M+H]^+^ 571.1720, found 571.1723.

**1-(4-Chlorophenyl)-2,6-ditosyl-1,2,3,4,5,6-hexahydroazepino[4,3-*b*]indole (3od)**

White solid, Yield (1) 80% (**Method B:** After the mixture was reacted for 3 h, **2d** (2equiv) was added), Yield (2) 68% (**Method A**), Mp 82–84 ºC. ^1^H NMR (400 MHz, CDCl_3_) *δ* 8.19 (d, *J* = 8.4 Hz, 1H), 7.59 (d, *J* = 8.4 Hz, 2H), 7.42 (d, *J* = 8.4 Hz, 2H), 7.36 – 7.30 (m, 1H), 7.25 – 7.21 (m, 4H), 7.19 (d, *J* = 8.8 Hz, 2H), 7.00 (d, *J* = 5.6 Hz, 2H), 6.98 (d, *J* = 6.0 Hz, 2H), 6.64 (s, 1H), 3.85 – 3.71 (m, 1H), 3.18 (dt, *J* = 14.4, 4.0 Hz, 1H), 3.04 (ddd, *J* = 9.6, 6.4, 2.8 Hz, 1H), 2.68 (ddd, *J* = 12.8, 9.2, 3.2 Hz, 1H), 2.39 (s, 3H), 2.34 (s, 3H), 1.67 – 1.52 (m, 2H); ^13^C NMR (100 MHz, CDCl_3_) *δ* 145.1, 143.6, 139.0, 137.0, 136.5, 136.2, 136.1, 133.7, 130.1, 129.6, 129.4, 129.0, 128.9, 126.9, 126.3, 124.8, 124.1, 120.0, 117.9, 115.1, 54.5, 45.1, 26.0, 25.2, 21.7, 21.6; HRMS (ESI) calcd for C_32_H_33_ClN_3_O_4_S_2_ [M+NH_4_]^+^ 622.1596, found 622.1596.

**1-(*p*-tolyl)-2,6-ditosyl-1,2,3,4,5,6-hexahydroazepino[4,3-*b*]indole (3oe)**

White solid, Yield (1) 79% (**Method B:** After the mixture was reacted for 3 h, **2e** (2equiv) was added), Yield (2) 65% (**Method A**), Mp 85–87 ºC. ^1^H NMR (400 MHz, CDCl_3_) *δ* 8.18 (d, *J* = 8.4 Hz, 1H), 7.58 (d, *J* = 8.4 Hz, 2H), 7.44 (d, *J* = 8.0 Hz, 2H), 7.35 – 7.28 (m, 1H), 7.25 – 7.19 (m, 4H), 7.04 – 6.97 (m, 4H), 6.91 (d, *J* = 8.0 Hz, 2H), 6.64 (s, 1H), 3.84 – 3.72 (m, 1H), 3.19 (dt, *J* = 14.4, 4.0 Hz, 1H), 3.12 – 3.00 (m, 1H), 2.79 – 2.64 (m, 1H), 2.38 (s, 3H), 2.34 (s, 3H), 2.29 (s, 3H), 1.61 (t, *J* = 5.6 Hz, 2H); ^13^C NMR (100 MHz, CDCl_3_) *δ* 145.0, 143.4, 139.0, 137.5, 137.4, 136.3, 136.2, 134.7, 130.0, 129.9, 129.4, 129.4, 127.6, 127.0, 126.3, 124.6, 124.0, 120.9, 118.1, 115.1, 54.9, 45.0, 26.0, 25.4, 21.7, 21.6, 21.1; HRMS (ESI) calcd for C_33_H_33_N_2_O_4_S_2_ [M+H]^+^ 585.1876, found 585.1885.

**1-Propyl-2,6-ditosyl-1,2,3,4,5,6-hexahydroazepino[4,3-*b*]indole (3oj)**

White solid, Yield (1) 62% (**Method B:** After the mixture was reacted for 3 h, **2j** (2equiv) was added), Yield (2) 60% (**Method A**), Mp 69–71 ºC. ^1^H NMR (400 MHz, CDCl_3_) *δ* 8.15 – 8.12 (m, 1H), 7.51 (d, *J* = 8.4 Hz, 2H), 7.44 (d, *J* = 8.4 Hz, 2H), 7.42 – 7.38 (m, 1H), 7.31 – 7.26 (m, 2H), 7.18 (d, *J* = 8.0 Hz, 2H), 7.00 (d, *J* = 8.0 Hz, 2H), 5.38 (dd, *J* = 9.6, 6.0 Hz, 1H), 4.03 – 3.88 (m, 1H), 3.44 – 3.37 (m, 1H), 3.05 (t, *J* = 6.0 Hz, 2H), 2.35 (s, 3H), 2.32 (s, 3H), 1.94 – 1.74 (m, 2H), 1.74 – 1.62 (m, 2H), 1.43 – 1.35 (m, 1H), 1.24 – 1.16 (m, 1H), 0.91 (t, *J* = 7.2 Hz, 3H); ^13^C NMR (100 MHz, CDCl_3_) *δ* 144.8, 143.1, 137.8, 137.5, 136.9, 136.3, 136.2, 132.0, 129.9, 129.3, 127.0, 126.2, 124.4, 123.8, 117.8, 115.2, 52.0, 44.1, 35.2, 26.7, 25.5, 21.7, 21.5, 20.0, 13.8; HRMS (ESI) calcd for C_29_H_32_N_2_O_4_S_2_ [M+H]^+^ 537.1876, found 537.1868.

**1-Cyclohexyl-2,6-ditosyl-1,2,3,4,5,6-hexahydroazepino[4,3-*b*]indole (3ol)**

White solid, Yield (1) 75% (**Method B:** After the mixture was reacted for 3 h, **2l** (2equiv) was added), Yield (2) 62% (**Method A**), Mp 87–88 ºC. ^1^H NMR (400 MHz, CDCl_3_) *δ* 8.17 – 8.12 (m, 1H), 7.43 (d, *J* = 8.8 Hz, 1H), 7.42 (d, *J* = 8.0 Hz, 2H), 7.33 (d, *J* = 8.4 Hz, 2H), 7.30 – 7.27 (m, 2H), 7.12 (d, *J* = 8.0 Hz, 2H), 6.85 (d, *J* = 8.0 Hz, 2H), 4.92 (d, *J* = 10.8 Hz, 1H), 4.14 – 4.03 (m, 1H), 3.56 – 3.48 (m, 1H), 3.42 – 3.34 (m, 1H), 2.88 – 2.80 (m, 1H), 2.33 (s, 3H), 2.24 (s, 3H), 1.95 – 1.57 (m, 6H), 1.52 – 1.32 (m, 4H), 1.52 – 1.32 (m, 3H); ^13^C NMR (100 MHz, CDCl_3_) *δ* 144.8, 142.8, 138.1, 137.5, 136.1, 135.8, 131.2, 129.7, 129.2, 127.0, 126.2, 124.4, 124.2, 124.1, 118.6, 115.8, 56.6, 45.0, 39.0, 30.7, 30.4, 26.4, 26.3, 26.0, 26.0, 25.7, 21.7, 21.5; HRMS (ESI) calcd for C_32_H_37_N_2_O_4_S_2_ [M+H]^+^ 577.2189, found 577.2192.

**1-Benzyl-2,6-ditosyl-1,2,3,4,5,6-hexahydroazepino[4,3-*b*]indole (3on)**

White solid, Yield (1) 80% (**Method B:** After the mixture was reacted for 3 h, **2n** (2equiv) was added), Yield (2) 71% (**Method A**), Mp 167–169 ºC. ^1^H NMR (400 MHz, CDCl_3_) *δ* 8.13 (d, *J* = 8.0 Hz, 1H), 7.56 (d, *J* = 8.4 Hz, 2H), 7.24 – 7.18 (m, 5H), 7.17 – 7.07 (m, 5H), 6.96 (t, *J* = 8.0 Hz, 4H), 5.62 (t, *J* = 8.0 Hz, 1H), 4.02 – 3.85 (m, 1H), 3.55 – 3.46 (m, 1H), 3.36 (ddd, *J* = 11.2, 8.4, 2.4 Hz, 1H), 3.23 (ddd, *J* = 11.6, 9.2, 2.4 Hz, 1H), 3.13 – 2.99 (m, 2H), 2.35 (s, 3H), 2.32 (s, 3H), 2.07 – 1.94 (m, 1H), 1.92 – 1.94 (m, 1H); ^13^C NMR (100 MHz, CDCl_3_) *δ* 144.9, 143.0, 138.0, 137.6, 137.4, 136.1, 136.1, 130.0, 129.4, 129.3, 129.1, 128.6, 127.1, 126.8, 126.4, 124.4, 123.8, 123.7, 117.8, 115.3, 54.3, 44.6, 39.7, 27.0, 25.6, 21.7, 21.6; HRMS (ESI) calcd for C_33_H_33_N_2_O_4_S_2_ [M+H]^+^ 585.1876, found 585.1880.

**1-(Thiophen-2-yl)-2,6-ditosyl-1,2,3,4,5,6-hexahydroazepino[4,3-*b*]indole (3oq)**

White solid, Yield (1) 48% (**Method B:** After the mixture was reacted for 3 h, **2q** (2equiv) was added), Yield (2) 32% (**Method A**), Mp 74–76 ºC. ^1^H NMR (400 MHz, CDCl_3_) *δ* 8.19 (d, *J* = 8.8 Hz, 1H), 7.55 (t, *J* = 8.4 Hz, 4H), 7.37 – 7.28 (m, 2H), 7.25 – 7.17 (m, 4H), 7.10 (d, *J* = 8.0 Hz, 2H), 6.78 (t, *J* = 4.4 Hz, 2H), 6.43– 6.43 (m, 1H), 3.90 – 3.79 (m, 1H), 3.36 (dt, *J* = 14.0, 5.2 Hz, 1H), 3.17 – 2.98 (m, 2H), 2.38 (s, 6H), 1.88 – 1.65 (m, 2H); ^13^C NMR (100 MHz, CDCl_3_) *δ* 145.0, 143.6, 142.5, 139.2, 137.4, 136.2, 136.1, 130.1, 129.9, 129.6, 129.2, 128.1, 127.2, 126.9, 126.3, 124.8, 124.1, 121.8, 118.0, 115.3, 51.9, 45.1, 25.9, 25.8, 21.8, 21.6; HRMS (ESI) calcd for C_30_H_32_N_3_O_4_S_3_ [M+NH_4_]^+^ 594.1549, found 594.1547.

**2,6-Ditosyl-1,2,3,4,5,6-hexahydroazepino[4,3-*b*]indole (3ok)**

White powder, Yield 75% (**Method B:** After the mixture was reacted for 3 h, 40% formaldehyde (2equiv) and trifluoroacetic (1.5 equiv) were added), Mp 157–159 ºC. ^1^H NMR (400 MHz, CDCl_3_) *δ* 8.15 (d, *J* = 7.2 Hz, 1H), 7.58 (d, *J* = 8.0 Hz, 2H), 7.49 (d, *J* = 8.4 Hz, 2H), 7.43 (dd, *J* = 6.4 Hz, 2.0 Hz, 1H), 7.32 – 7.26 (m, 2H), 7.20 (d, *J* = 8.0 Hz, 2H), 7.11 (d, *J* = 8.0 Hz, 2H), 4.49 (s, 2H), 3.54 (t, *J* = 6.0 Hz,2H), 3.12 (t, *J* = 6.0 Hz,2H), 2.37 (s, 3H), 2.35 (s, 3H), 1.97 – 1.90 (m, 2H); ^13^C NMR (100 MHz, CDCl_3_) *δ* 145.0, 143.5, 138.1, 136.3, 136.1, 130.1, 129.6, 128.8, 127.1, 126.3, 124.5, 123.7, 118.4, 117.8, 114.9, 49.6, 41.7, 26.0, 25.9, 21.7, 21.6; HRMS (ESI) calcd for C_26_H_26_N_2_O_4_S_2_ [M+H]^+^ 495.1407, found 495.1412.

**2,7-Ditosyl-2,3,4,5,6,7-hexahydro-1*H*-azocino[4,3-*b*]indole (3pk)**

White solid, Yield 73% (**Method B:** After the mixture was reacted for 3 h, 40% formaldehyde (2equiv) and trifluoroacetic (1.5 equiv) were added), Mp 79–80 ºC.^1^H NMR (400 MHz, CDCl_3_) *δ* 8.22 (d, *J* = 8.4 Hz, 1H), 7.69 (d, *J* = 8.0 Hz, 2H), 7.63 (d, *J* = 8.0 Hz, 2H), 7.41 (d, *J* = 7.6 Hz, 1H), 7.30 (d, *J* = 8.0 Hz, 3H), 7.20 (d, *J* = 8.0 Hz, 3H), 4.44 (s, 2H), 3.35 (t, *J* = 6.0 Hz, 2H), 3.24 (t, *J* = 5.2 Hz , 2H), 2.44 (s, 3H), 2.35 (s, 3H), 1.85 – 1.75 (m, 2H), 1.57 – 1.48 (m, 2H);  ^13^C NMR (100 MHz, CDCl_3_) *δ*145.0, 143.5, 137.7, 136.6, 136.4, 136.0, 130.1, 129.9, 128.6, 127.3, 126.3, 124.6, 123.6, 118.3, 116.0, 114.9, 48.2, 42.9, 27.4, 25.7, 23.5, 21.7, 21.6; HRMS (ESI) calcd for C_27_H_28_N_2_O_4_S_2_ [M+H]^+^ 509.1563, found 509.1570.

**2,8-Ditosyl-1,2,3,4,5,6,7,8-octahydroazonino[4,3-*b*]indole (3qk)**

White solid, Yield 53% (**Method B:** After the mixture was reacted for 3 h, 40% formaldehyde (2equiv) and trifluoroacetic (1.5 equiv) were added), Mp 82–83 ºC. ^1^H NMR (400 MHz, CDCl_3_) δ 8.18 (d, *J* = 8.0 Hz, 1H), 7.73 (d, *J* = 8.0 Hz, 2H), 7.57 (d, *J* = 8.0 Hz, 2H), 7.47 (d, *J* = 7.6 Hz, 1H), 7.31 (d, *J* = 8.0 Hz, 2H), 7.29 – 7.19 (m, 2H), 7.15 (d, *J* = 8.0 Hz, 2H), 4.34 (s, 2H), 3.34 (t, *J* = 6.0 Hz, 2H), 3.12 (t, *J* = 5.2 Hz, 2H), 2.44 (s, 3H), 2.32 (s, 3H), 2.04 – 1.92 (m, 3H), 1.68 – 1.57 (m, 3H); ^13^C NMR (100 MHz, CDCl_3_) *δ* 144.9, 143.6, 139.8, 136.7, 136.0, 135.8, 129.9, 129.6, 127.5, 126.3, 124.6, 123.8, 118.6, 117.3, 115.2, 51.4, 45.8, 30.5, 28.6, 26.2, 23.0, 21.7; HRMS (ESI) calcd for C_28_H_31_N_2_O_4_S_2_ [M+H]^+^ 523.1720, found 523.1725.

**Formal synthesis of eraticine and conolidine**

**Synthesis of 2,3,4,5,6,7-hexahydro-1*H*-azocino[4,3-*b*]indole (4pk)**

To a stirred solution of naphthalene (5.60 g, 43.8 mmol) in anhydrous THF (100 mL) under argon was added sodium (0.92 g, 40.00 mmol) at rt and the mixture was stirred until the formation of sodium naphthalide was complete, and then cooled to -78 °C. A solution of **3pk** (1.02 g, 2.00 mmol) in anhydrous THF (15 mL) was added to the above solution at -78 °C and the resulting mixture was stirred at the same temperature for 1 h. The reaction was quenched by the addition of sat. *aq* NH_4_Cl (35 mL) and the organic layer was washed with water (2 × 30 mL), brine (3 × 30 mL), dried over anhydrous Na_2_SO_4_ and concentrated in vacuo. The residue was purified by flash chromatography on silica gel using dichloromethane and methanol (v/v = 20:1) as eluent to give the title compound as white solid (0.38 g, 95 %); Mp 265–267 ºC. ^1^H NMR (400 MHz, MeOD-*d_4_*) *δ* 7.58 (d, *J* = 8.0 Hz, 1H), 7.35 (d, *J* = 7.6 Hz, 1H), 7.15 – 7.05 (m, 2H), 4.49 (s, 2H), 3.06 – 2.98 (m, 2H), 2.94 – 2.86 (m, 2H), 1.97 – 1.87 (m, 4H); ^13^C NMR (100 MHz, MeOD-*d_4_*) *δ* 142.2, 137.1, 128.8, 122.6, 120.9, 118.2, 112.2, 100.7, 43.8, 39.0, 29.0, 26.5, 24.7; HRMS (ESI) calcd for C_13_H_7_N_2_ [M+H]^+^ 201.1386, found 201.1389.

**Synthesis of *tert*-Butyl 3,4,5,6-tetrahydro-1*H*-azocino[4,3-*b*]indole-2(7*H*)-carboxylate (5pk)**

To a solution of **4pk** (0.38 g, 1.90 mmol) in dichloromethane (50 mL) were added trimethylamine (0.38 g, 3.76 mmol) and (Boc)_2_O (0.83 g, 0.38 mmol), and the mixture was stirred at room temperature under argon atmosphere for 12 hours. H_2_O (30 mL) were added to the resulting mixture when the reaction was complete as monitored by TLC. After separation of the organic layer, the water layer was extracted with DCM (3×30 mL). The combined organic layer was dried over anhydrous Na_2_SO_4_, filtered, evaporated and purified via column chromatography on silica gel (eluent: petroleum ether:ethyl acetate = 10:1) to afford the desired product **5pk** as white solid (0.53 g, 93%); Mp 50–52ºC. ^1^H NMR (400 MHz, CDCl_3_) *δ* 7.79 (br, 1H), 7.48 (d, *J* = 7.2 Hz, 1H), 7.32 – 7.27 (m, 1H), 7.19 – 7.04 (m, 2H), 4.75 and 4.69 (s, 2H), 3.52 and 3.42 (t, *J* = 4.8 Hz, 2H), 2.93 – 2.77 (m, 2H), 1.86 – 1.69 (m, 4H), 1.50 and 1.43 (s, 9H); ^13^C NMR (100 MHz, CDCl_3_) *δ* 155.9, 135.9, 134.9 and 134.8, 128.0 and 127.7, 121.0, 119.2 and 119.1, 117.7 and 117.6, 110.8 and 110.6, 108.2 and 107.2, 79.8 and 79.6, 46.4 and 46.3, 43.0 and 42.8, 28.8 and 28.7, 27.2 and 27.1, 27.0 and 26.1, 25.9 and 25.5; HRMS (ESI) calcd for C_18_H_25_N_2_O_2_ [M+H]^+^ 301.1911, found 301.1915.

**Synthesis of *tert*-butyl 6-oxo-3,4,5,6-tetrahydro-1*H*-azocino[4,3-b]indole-2(7*H*)-carboxylate (6pk)**

In a round bottom flask, **5pk** (0.15g, 0.50 mmol) and SeO_2_ (0.061g, 0.55mmol) was dissolved in 1,4-dioxane (30 mL), and the mixture was stirred at 80 ºC for 18 hours. H_2_O (30 mL) was added to the resulting mixture. After separation of the organic layer, the water layer was extracted with DCM (3×30 mL). The combined organic layer was dried over anhydrous Na_2_SO_4_, filtered, evaporated and purified via column chromatography on silica gel (eluent: petroleum ether:ethyl acetate = 10:1) to afford the desired product **6pk** as yellow solid (0.088g, 56%); Mp 57–59 ºC. ^1^H NMR (400 MHz, CDCl_3_) *δ* 9.48 (d, *J* = 23.6 Hz, 1H), 7.75 (dd, *J* = 20.0, 8.0 Hz, 1H), 7.41 (d, *J* = 8.0 Hz, 1H), 7.35 (t, *J* = 7.6 Hz, 1H), 7.16 (t, *J* = 6.8 Hz, 1H), 5.01 and 4.88 (s, 2H), 3.62 and 3.48 (m, 1H), 2.97 (m, 2H), 2.04 and 1.96 (m, 2H), 1.49 and 1.30 (s, 9H); ^13^C NMR (100 MHz, CDCl_3_) *δ* 193.6 and 193.0, 155.5 and 155.4, 136.2 and 135.9, 134.1 and 132.8, 128.1 and 127.7, 126.9 and 126.7, 121.2 and 121.1,121.0 and 120.6, 119.6 and 117.6, 112.2, 80.3 and 80.2, 46.4 and 43.3, 42.8 and 42.7, 39.7 and 38.9, 28.6 and 28.5, 25.2 and 24.1; HRMS (ESI) calcd for C_18_H_22_N_2_NaO_3_ [M+Na]^+^ 337.1523, found 337.1515.

**Formal synthesis of latrepirdine**

**8-methyl-2,5-ditosyl-2,3,4,5-tetrahydro-1*H*-pyrido[4,3-b]indole (3fk)**

Reaction between **1f** and formaldehyde by means of the method described for the synthesis of **3ak** gave **3fk** as a white solid in 95% yield, Mp 215–216 ºC. ^1^H NMR (400 MHz, CDCl_3_) *δ* 7.98 (d, *J* = 8.4 Hz, 1H), 7.73 (d, *J* = 8.0 Hz, 2H), 7.60 (d, *J* = 8.4 Hz, 2H), 7.33 (d, *J* = 8.4 Hz, 2H), 7.19 (d, *J* = 8.0 Hz, 2H), 7.10 (d, *J* = 8.4 Hz, 1H), 7.06 (s, 1H), 4.16 (s, 2H), 3.41 (t, *J* = 5.6 Hz, 2H), 3.19 (t, *J* = 5.6 Hz, 2H), 2.43 (s, 3H), 2.39 (s, 3H), 2.34 (s, 3H); ^13^C NMR (100 MHz, CDCl_3_) *δ* 145.0, 144.0, 135.6, 134.5, 133.4, 133.2, 132.4, 130.0, 129.9, 127.8, 127.7, 126.4, 126.1, 117.9, 114.4, 114.2, 43.7, 42.7, 25.4, 21.6, 21.3; HRMS (ESI) calcd for C_26_H_27_N_2_O_4_S_2_ [M+H]^+^ 495.1407, found 495.1406.

**Synthesis of 8-methyl-2,3,4,5-tetrahydro-1*H*-pyrido[4,3-*b*]indole (4fk)**

To a stirred solution of naphthalene (5.60 g, 43.8 mmol) in anhydrous THF (100 mL) under argon was added sodium (0.92 g, 40.00 mmol) at rt and the mixture was stirred until the formation of sodium naphthalide was complete, and then cooled to -78 °C. A solution of **3fk** (1.00 g, 2.02 mmol) in anhydrous THF (15 mL) was added to the above solution at -78 °C and the resulting mixture was stirred at the same temperature for 1 h. The reaction was quenched by the addition of sat. *aq* NH_4_Cl (35 mL) and the organic layer was washed with water (2 × 30 mL), brine (3 × 30 mL), dried over anhydrous Na_2_SO_4_ and concentrated in vacuo. The residue was purified by flash chromatography on silica gel using dichloromethane and methanol (v/v = 20:1) as eluent to give the title compound as white solid (0.34 g, 91 %); Mp 254–256 ºC. ^1^H NMR (400 MHz, MeOD-*d_4_*) *δ* 7.24 – 7.19 (m, 2H), 6.97 (d, *J* = 8.4 Hz, 1H), 4.39 (s, 2H), 3.60 (t, *J* = 6.0 Hz, 2H), 3.13 (t, *J* = 6.0 Hz, 2H), 2.40 (s, 3H); ^13^C NMR (100 MHz, MeOD-*d_4_*) *δ* 136.3, 130.8, 129.8, 126.7, 124.6, 118.0, 111.9, 102.1, 43.1, 42.4, 21.6, 21.4; HRMS (ESI) calcd for C_12_H_15_N_2_ [M+H]^+^ 187.1230, found 187.1235.

**Synthesis of 2,8-dimethyl-2,3,4,5-tetrahydro-1*H*-pyrido[4,3-b]indole (5fk)**

**4fk** (0.34 g, 1.83 mmol) and NaCNBH_3_ (0.28 g, 4.44 mmol) were added to a round bottomed flask, dissolved in MeOH, and treated with 40% solution of formaldehyde in water (1.36 g, 18.13 mmol). This mixture was stirred for 1 h, after which, 2N HCl (10 mL) was added, followed by stirring for 15 min. The mixture was taken to pH = 11 by addition of concentrated, aqueous NaOH and extracted with DCM. The combined organic layer was dried over anhydrous Na_2_SO_4_, filtered, evaporated and purified via column chromatography on silica gel (eluent: petroleum ether:ethyl acetate = 10:1) to afford the desired product **4fk** as a white solid (0.33 g, 89%); Mp 174–175 ºC. ^1^H NMR (400 MHz, CDCl_3_) *δ* 8.40 (br, 1H), 7.15 (s, 1H), 7.11 (d, *J* = 8.0 Hz, 1H), 6.92 (d, *J* = 8.0 Hz, 1H), 3.73 (s, 2H), 2.86 (t, *J* = 5.2 Hz, 2H), 2.79 (t, *J* = 5.2 Hz, 2H), 2.58 (s, 3H), 2.42 (s, 3H); ^13^C NMR (100 MHz, CDCl_3_) *δ* 134.5, 131.4, 128.5, 126.1, 122.8, 117.3, 110.4, 106.8, 52.1, 51.4, 45.0, 23.0, 21.5; HRMS (ESI) calcd for C_13_H_16_N_2_ [M+H]^+^ 201.1386, found 201.1390.

**Reference**

1. [Trost](http://pubs.acs.org/action/doSearch?ContribStored=Trost%2C+B+M), B. M., [Machacek](http://pubs.acs.org/action/doSearch?ContribStored=Machacek%2C+M+R), M. R. & [Faulk](http://pubs.acs.org/action/doSearch?ContribStored=Faulk%2C+B+D), B. D. Sequential Ru−Pd catalysis:  A two-catalyst one-pot protocol for the synthesis of *N*- and *O*-heterocycles. *J. Am. Chem. Soc.* 2006, **128**, 6745**–**6754(2006)

2. Zhu, C. & Ma, S. M. Sc(OTf)_3_-catalyzed bicyclization of *o*-alkynylanilines with aldehydes: Ring-fused 1,2-dihydroquinolines. *Angew. Chem., Int. Ed.* 53, 13532–13535 (2014).

The ORTEP of 3aa, 3ak, 3pk, and 3oq


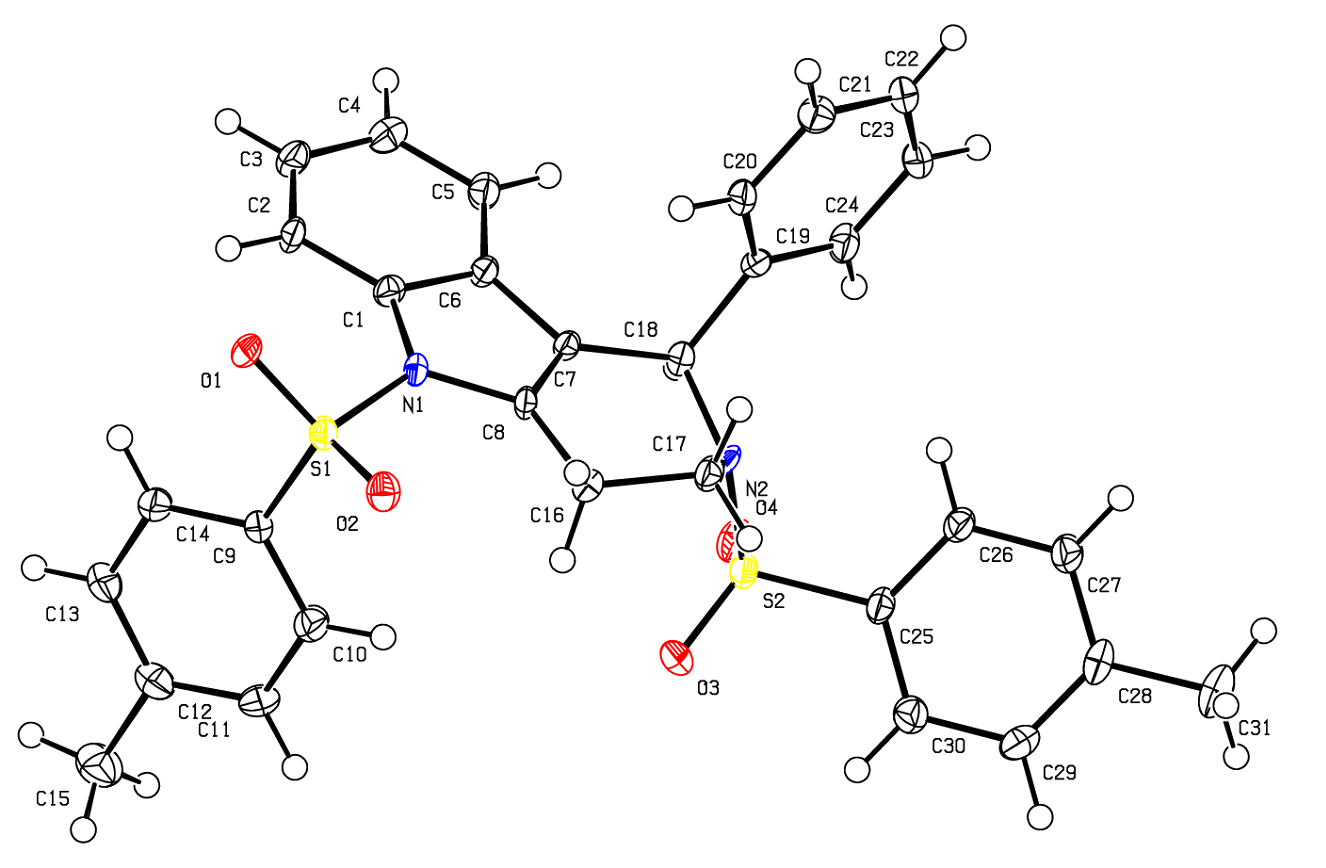


Table 1. Crystal data and structure refinement for 3aa.

| Empirical formula | C31 H28 N2 O4 S2 |
| --- | --- |
| Formula weight | 556.67 |
| Temperature | 113(2) K |
| Wavelength | 0.71073 A |
| Crystal system, space group | Triclinic, P-1 |
| Unit cell dimensions | a = 9.882(2) A alpha = 72.26(3) deg.  b = 10.111(2) A beta = 73.53(3) deg.  c = 15.242(3) A gamma = 73.25(3) deg. |
| Volume | 1356.7(5) A^3 |
| Z, Calculated density | 2, 1.363 Mg/m^3 |
| Absorption coefficient | 0.237 mm^-1 |
| F(000) | 584 |
| Crystal size | 0.20 x 0.18 x 0.12 mm |
| Theta range for data collection | 1.44 to 27.92 deg. |
| Limiting indices | -12<=h<=13, -12<=k<=13, -16<=l<=19 |
| Reflections collected / unique | 13465 / 6305 [R(int) = 0.0626] |
| Completeness to theta = 27.93 | 97.1 % |
| Absorption correction | Semi-empirical from equivalents |
| Max. and min. transmission | 0.9721 and 0.9541 |
| Refinement method | Full-matrix least-squares on F^2 |
| Data / restraints / parameters | 6305 / 0 / 355 |
| Goodness-of-fit on F^2 | 1.027 |
| Final R indices [I>2sigma(I)] | R1 = 0.0494, wR2 = 0.0981 |
| R indices (all data) | R1 = 0.0934, wR2 = 0.1354 |
| Extinction coefficient | 0.0135(11) |
| Largest diff. peak and hole | 0.374 and -0.641 e.A^-3 |


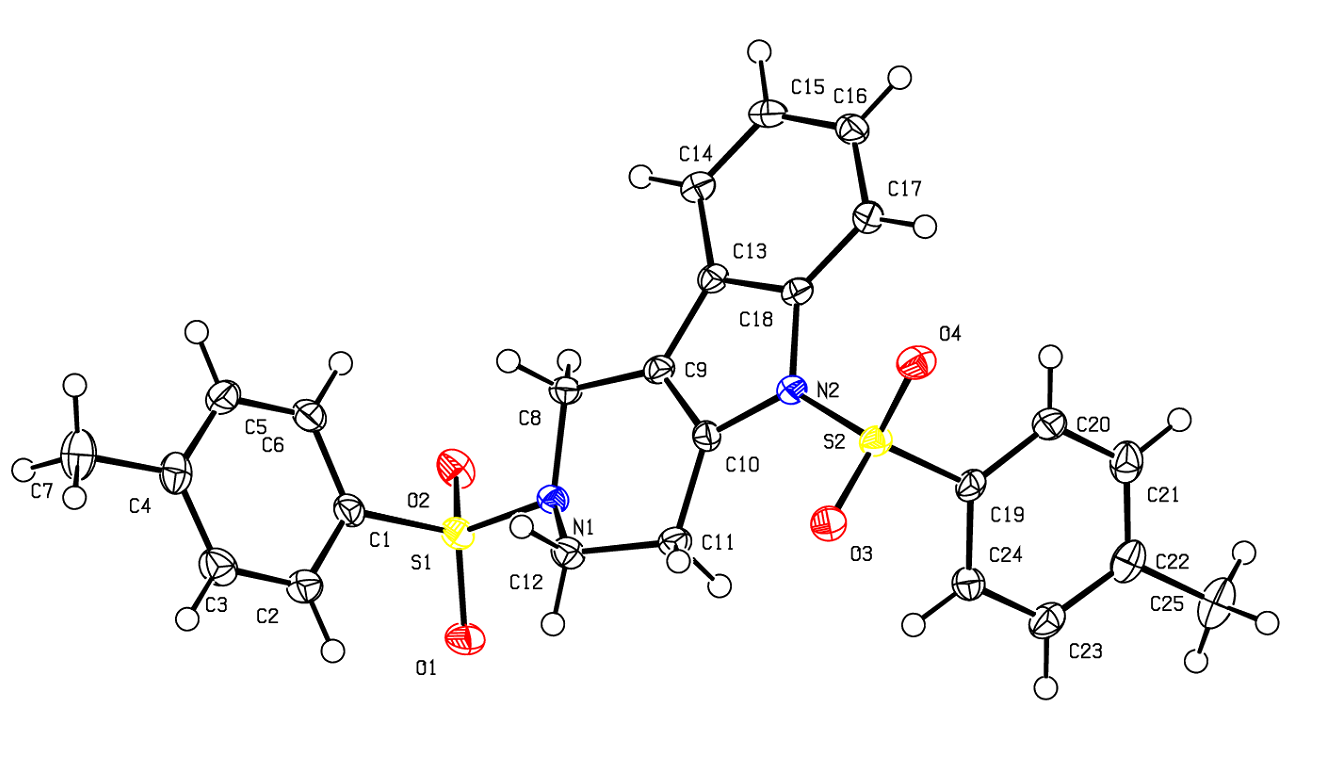


Table 2. Crystal data and structure refinement for 3ak.

| Empirical formula | C25 H24 N2 O4 S2 |
| --- | --- |
| Formula weight | 480.58 |
| Temperature | 113(2) K |
| Wavelength | 0.71073 A |
| Crystal system, space group | Triclinic, P-1 |
| Unit cell dimensions | a = 7.5834(15) A alpha = 103.22(3) deg.  b = 10.629(2) A beta = 101.43(3) deg.  c = 14.722(3) A gamma = 94.11(3) deg. |
| Volume | 1123.7(4) A^3 |
| Z, Calculated density | 2, 1.420 Mg/m^3 |
| Absorption coefficient | 0.273 mm^-1 |
| F(000) | 504 |
| Crystal size | 0.20 x 0.18 x 0.12 mm |
| Theta range for data collection | 1.46 to 28.02 deg. |
| Limiting indices | -9<=h<=9, -13<=k<=13, -19<=l<=19 |
| Reflections collected / unique | 11668 / 5298 [R(int) = 0.0392] |
| Completeness to theta = 28.02 | 97.7 % |
| Absorption correction | Semi-empirical from equivalents |
| Max. and min. transmission | 0.9679 and 0.9474 |
| Refinement method | Full-matrix least-squares on F^2 |
| Data / restraints / parameters | 5298 / 0 / 300 |
| Goodness-of-fit on F^2 | 0.996 |
| Final R indices [I>2sigma(I)] | R1 = 0.0454, wR2 = 0.1073 |
| R indices (all data) | R1 = 0.0652, wR2 = 0.1193 |
| Largest diff. peak and hole | 0.374 and -0.641 e.A^-3 |


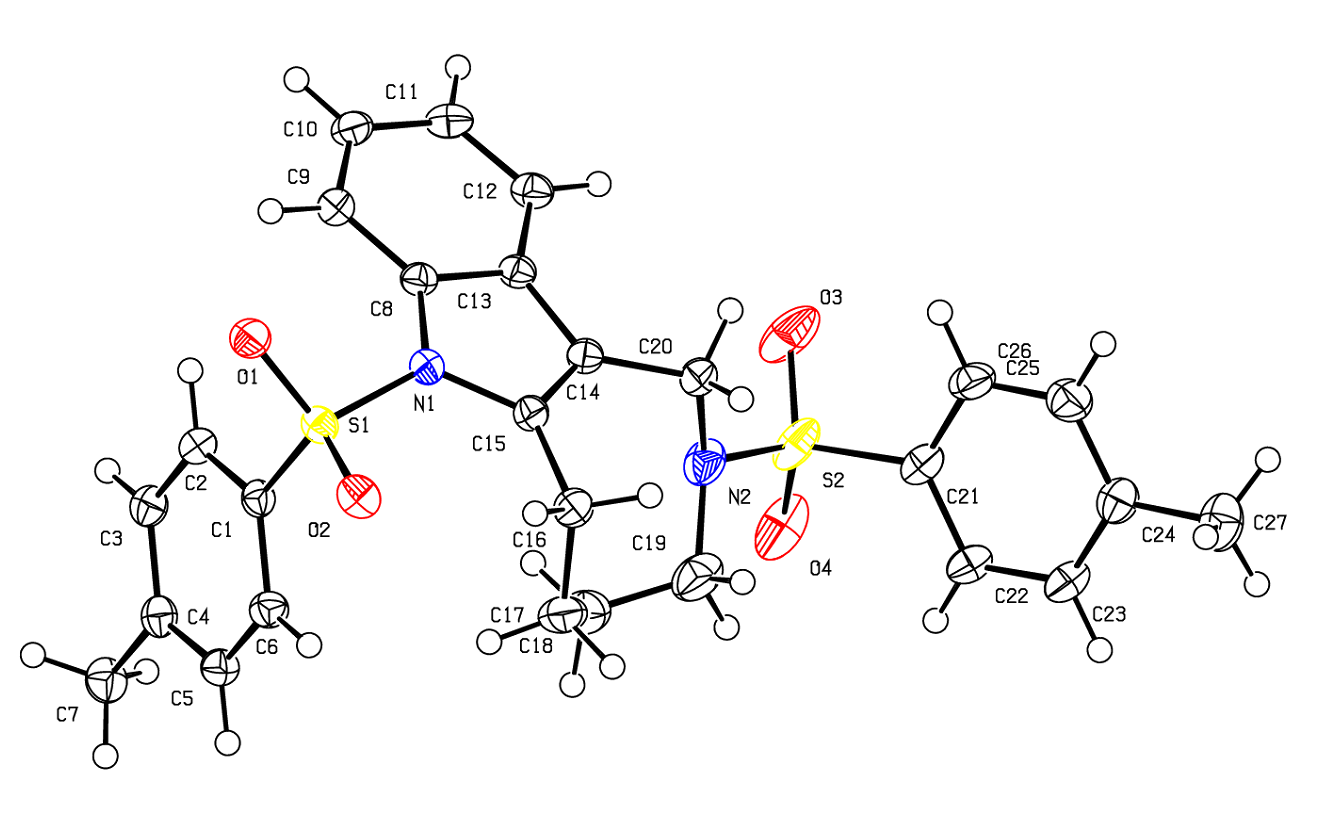


Table 3. Crystal data and structure refinement for 3pk.

| Empirical formula | C27 H28 N2 O4 S2 |
| --- | --- |
| Formula weight | 508.63 |
| Temperature | 113(2) K |
| Wavelength | 0.71073 A |
| Crystal system, space group | Monoclinic, P2(1)/n |
| Unit cell dimensions | a = 9.960(2) A alpha = 90 deg.  b = 22.894(5) A beta = 100.04(3) deg.  c = 10.819(2) A gamma = 90 deg. |
| Volume | 2429.2(8) A^3 |
| Z, Calculated density | 4, 1.391 Mg/m^3 |
| Absorption coefficient | 0.257 mm^-1 |
| F(000) | 1072 |
| Crystal size | 0.20 x 0.18 x 0.12 mm |
| Theta range for data collection | 1.78 to 27.92 deg. |
| Limiting indices | -13<=h<=12, -26<=k<=30, -14<=l<=14 |
| Reflections collected / unique | 24231 / 5798 [R(int) = 0.0453] |
| Completeness to theta = 27.92 | 99.7 % |
| Absorption correction | Semi-empirical from equivalents |
| Max. and min. transmission | 0.9698 and 0.9504 |
| Refinement method | Full-matrix least-squares on F^2 |
| Data / restraints / parameters | 5798 / 0 / 319 |
| Goodness-of-fit on F^2 | 1.019 |
| Final R indices [I>2sigma(I)] | R1 = 0.0602, wR2 = 0.1575 |
| R indices (all data) | R1 = 0.0700, wR2 = 0.1665 |
| Extinction coefficient | 0.0121(16) |
| Largest diff. peak and hole | 1.359 and -0.837 e.A^-3 |


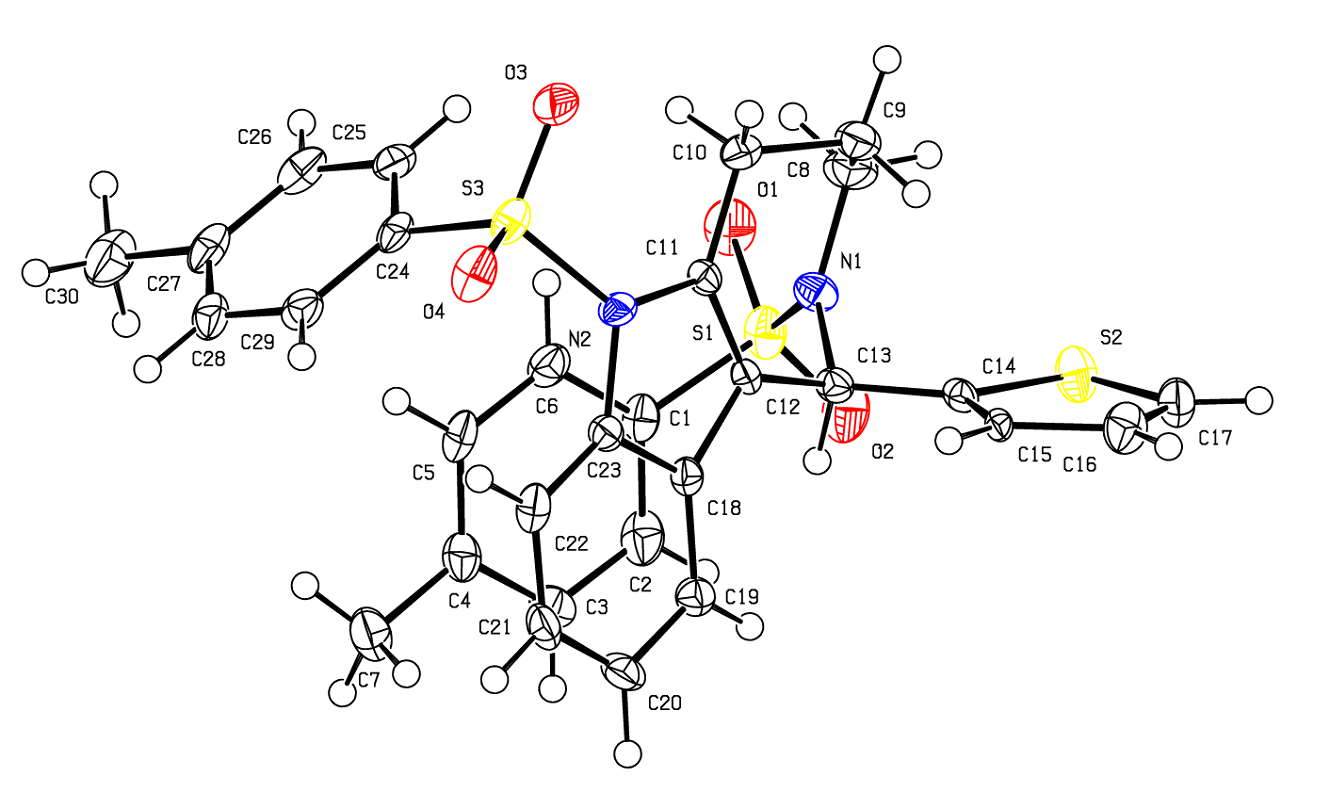


Table 4. Crystal data and structure refinement for 3oq.

| Empirical formula | C30 H28 N2 O4 S3 |
| --- | --- |
| Formula weight | 576.72 |
| Temperature | 113(2) K |
| Wavelength | 0.71073 A |
| Crystal system, space group | Triclinic, P-1 |
| Unit cell dimensions | a = 8.4379(17) A alpha = 99.40(3) deg  b = 11.874(2) A beta = 90.49(3) deg.  c = 13.696(3) A gamma = 98.37(3) deg. |
| Volume | 1338.7(5) A^3 |
| Z, Calculated density | 2, 1.431 Mg/m^3 |
| Absorption coefficient | 0.318 mm^-1 |
| F(000) | 604 |
| Crystal size | 0.20 x 0.18 x 0.12 mm |
| Theta range for data collection | 1.51 to 25.01 deg. |
| Limiting indices | -10<=h<=10, -14<=k<=13, -16<=l<=16 |
| Reflections collected / unique | 11300 / 4690 [R(int) = 0.0643] |
| Completeness to theta = 25.01 | 99.5 % |
| Absorption correction | Semi-empirical from equivalents |
| Max. and min. transmission | 0.9629 and 0.9392 |
| Refinement method | Full-matrix least-squares on F^2 |
| Data / restraints / parameters | 4690 / 0 / 354 |
| Goodness-of-fit on F^2 | 0.940 |
| Final R indices [I>2sigma(I)] | R1 = 0.0453, wR2 = 0.1060 |
| R indices (all data) | R1 = 0.0900, wR2 = 0.1334 |
| Largest diff. peak and hole | 0.339 and -0.431 e.A^-3 |

Copies of ^1^H, ^13^C spectra


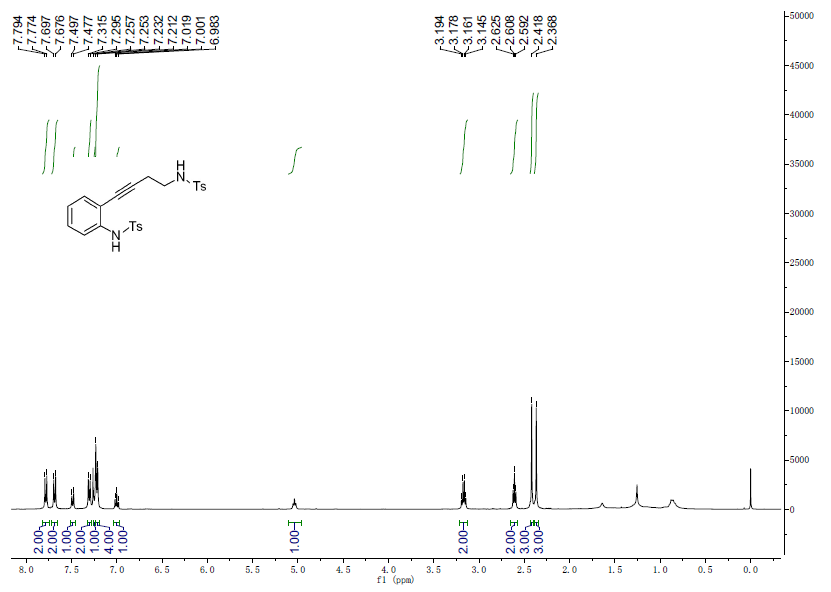


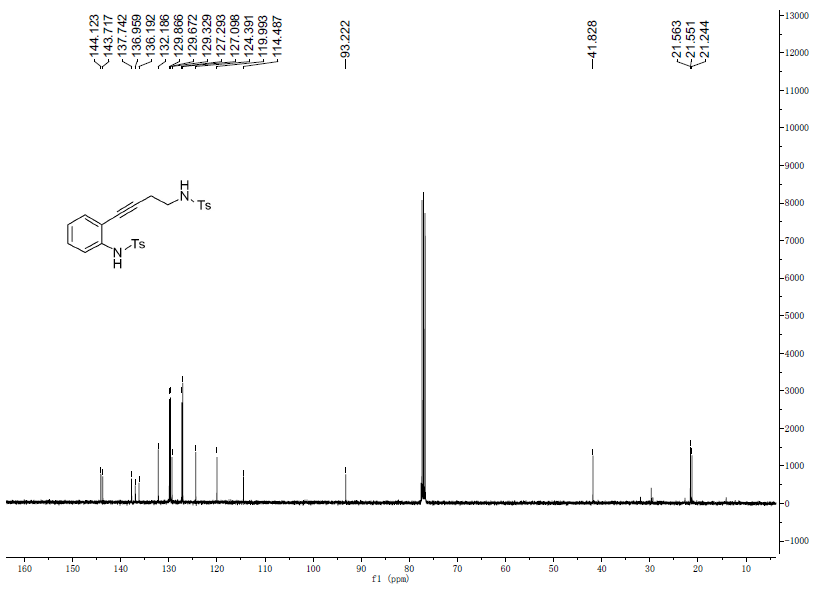


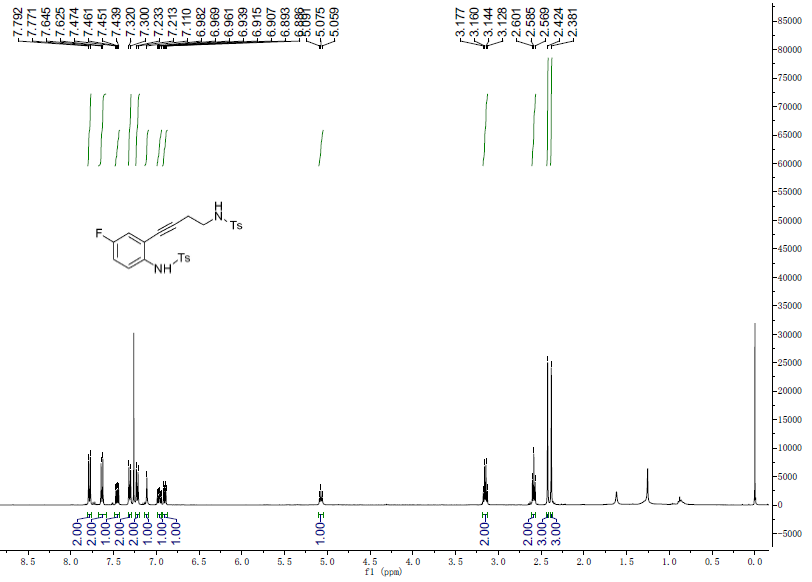


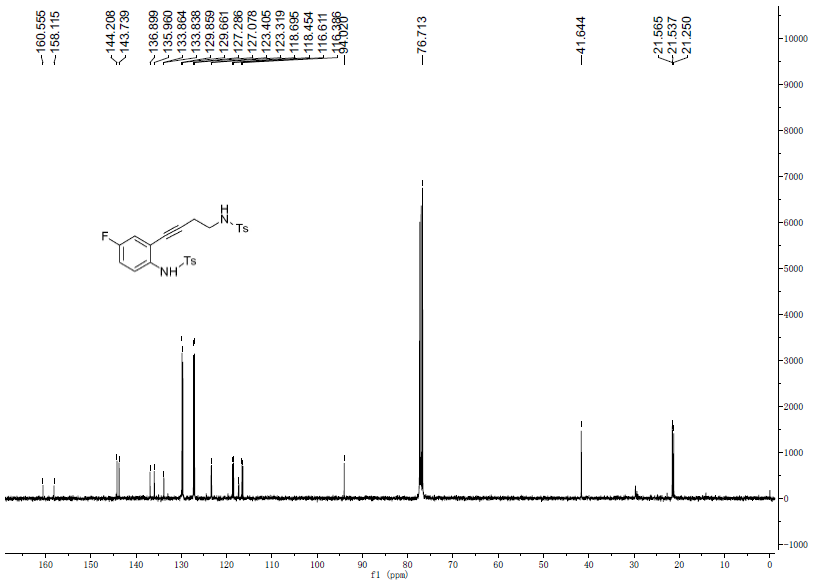


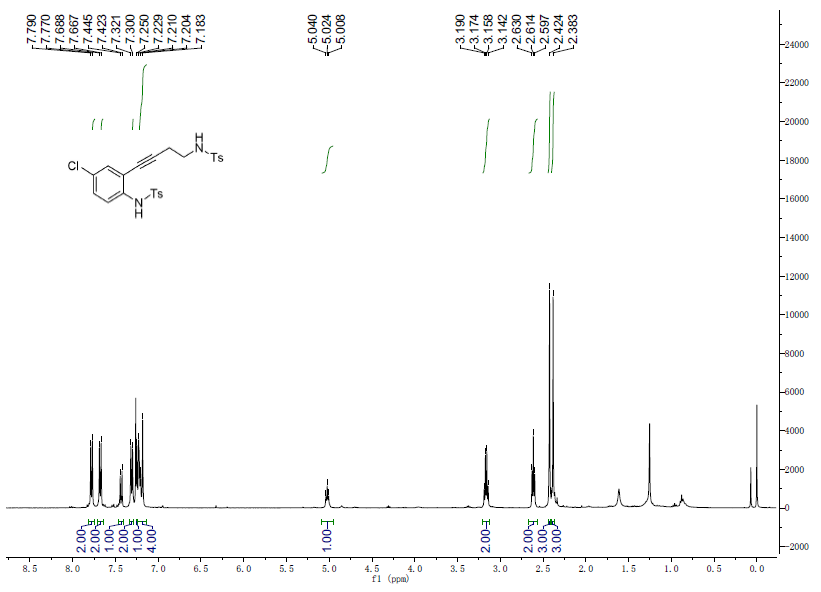


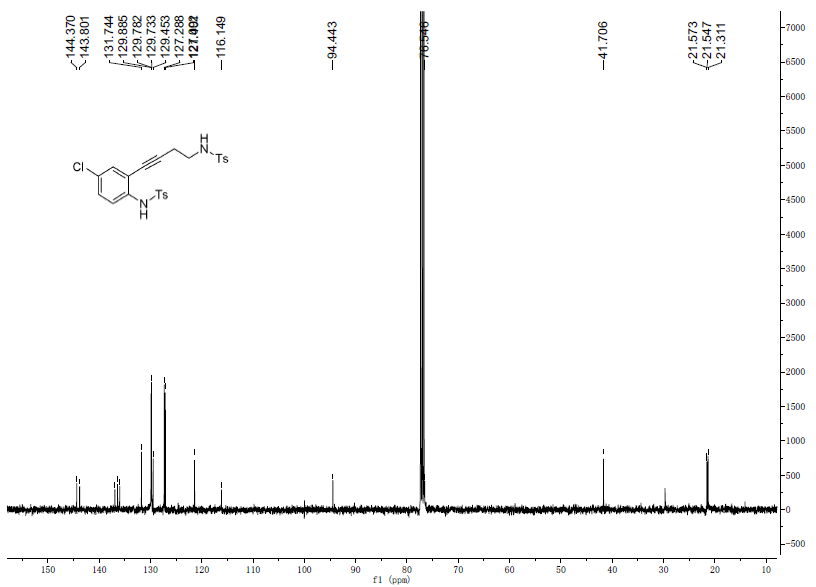


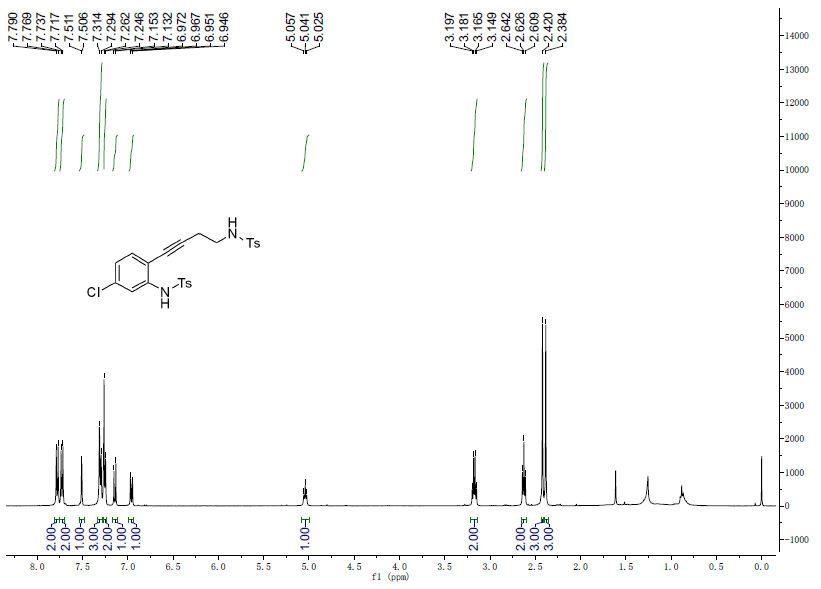


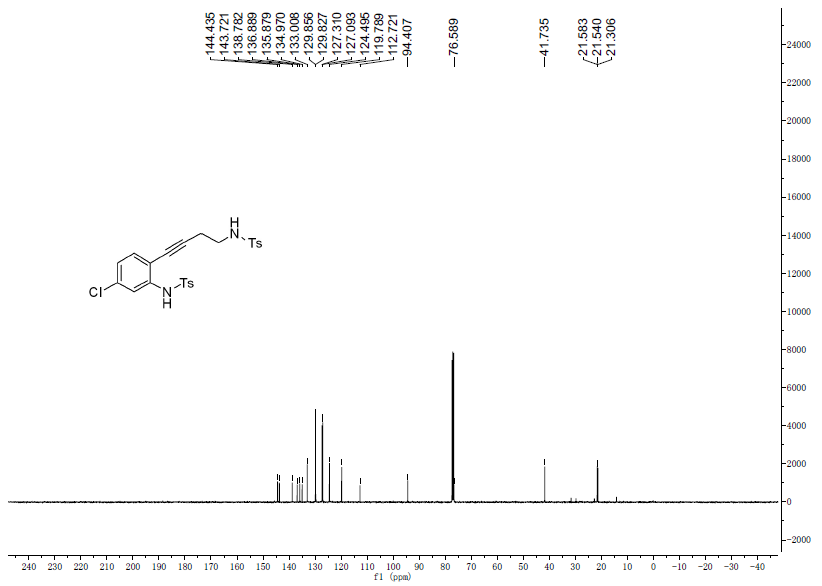


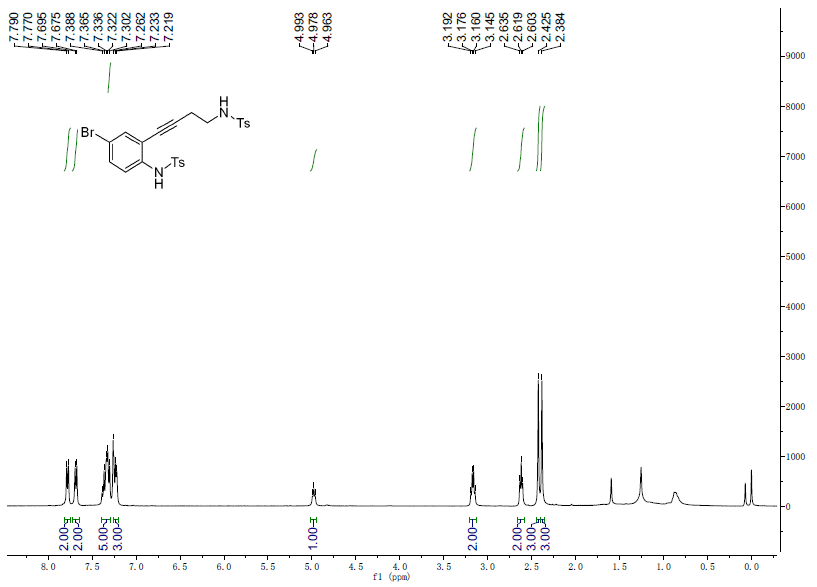


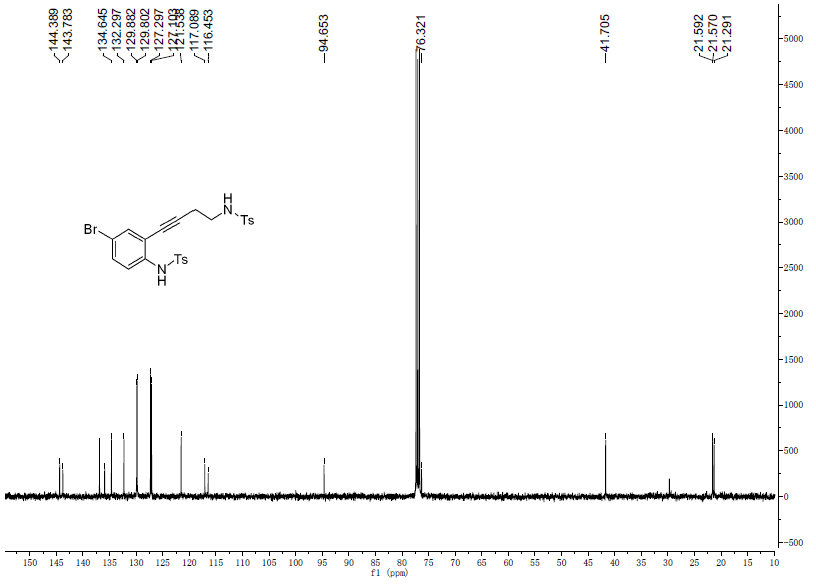


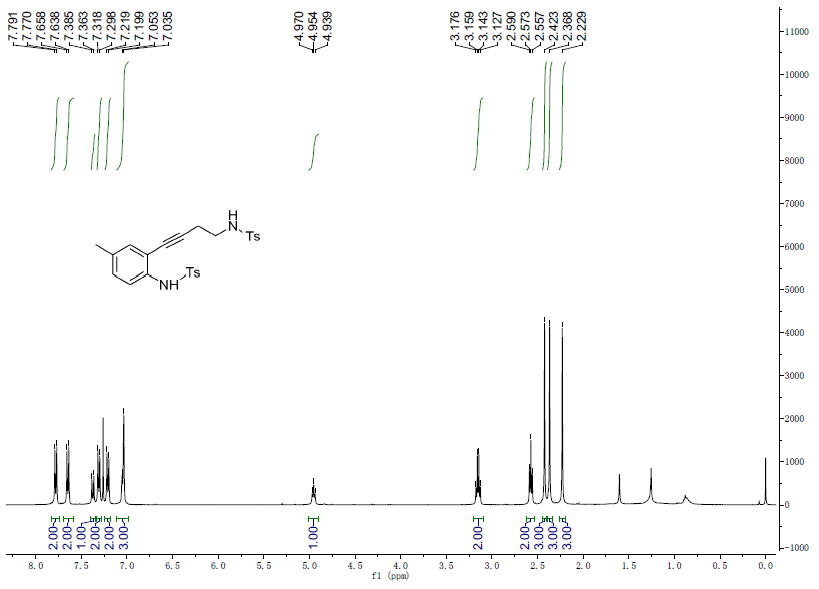


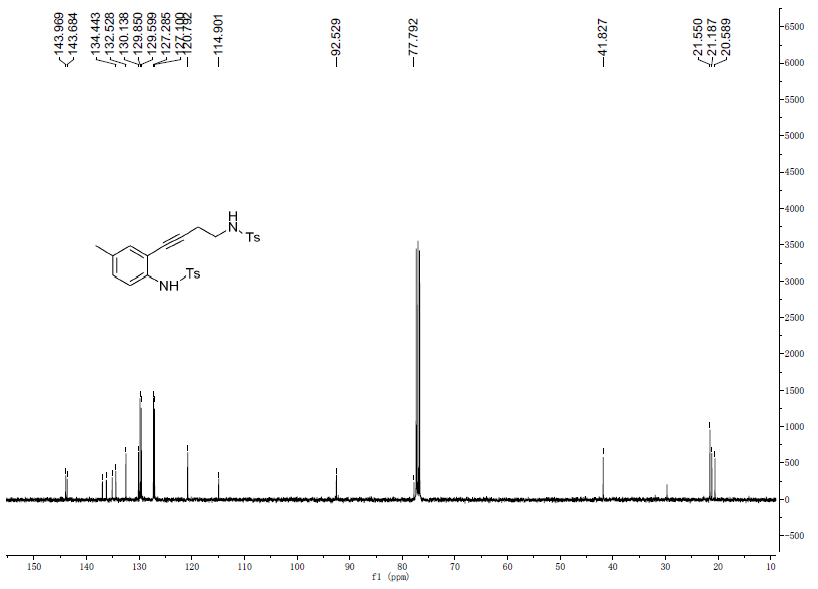


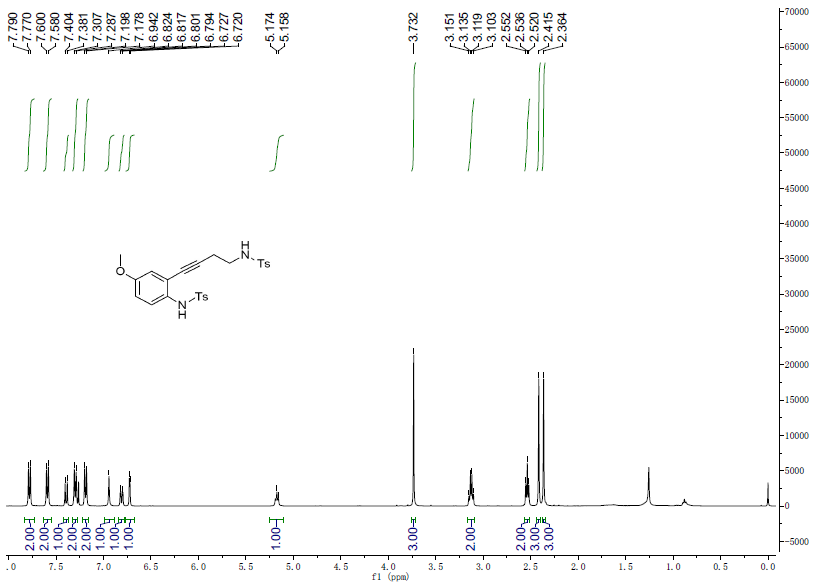


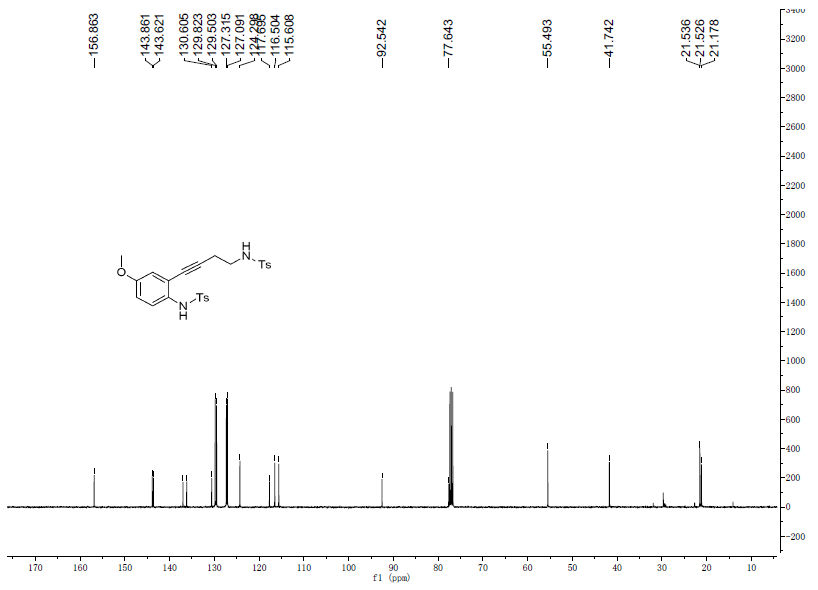


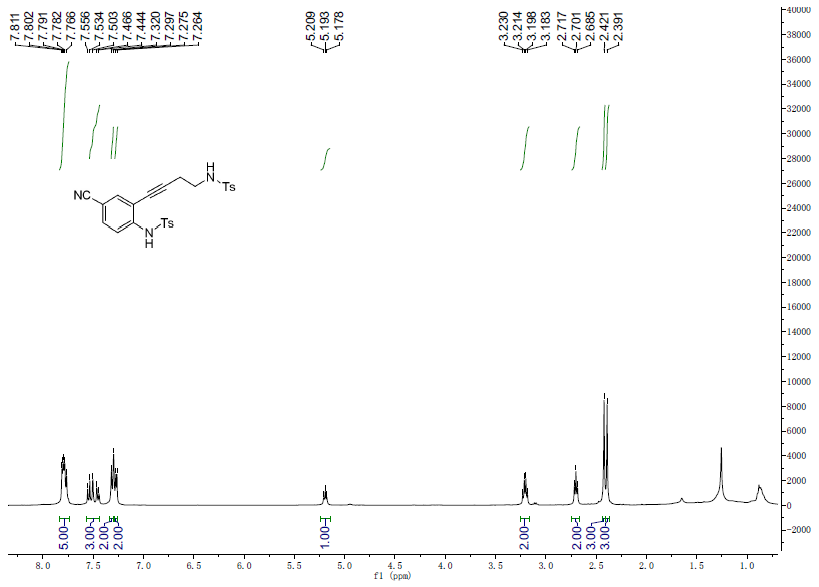


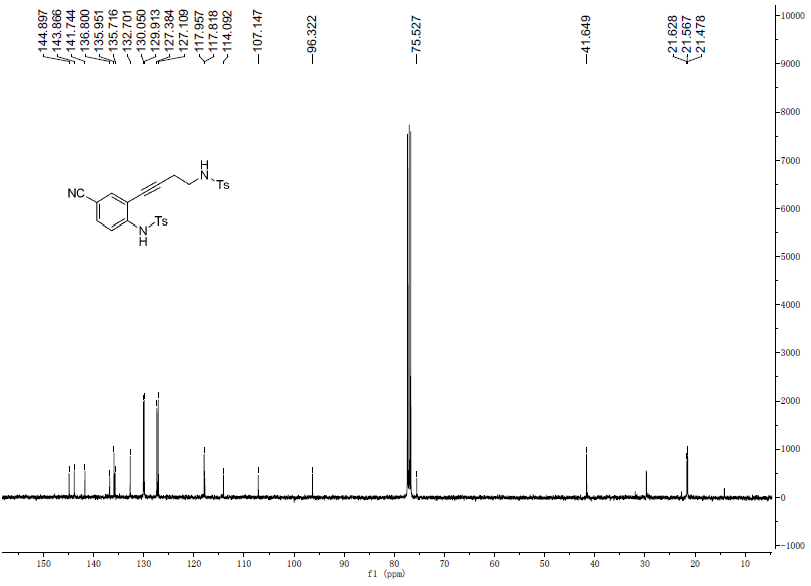


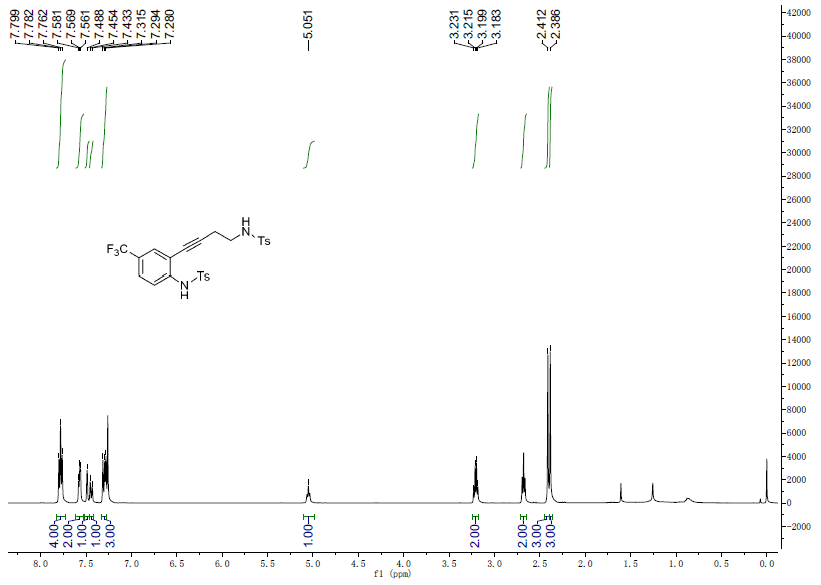


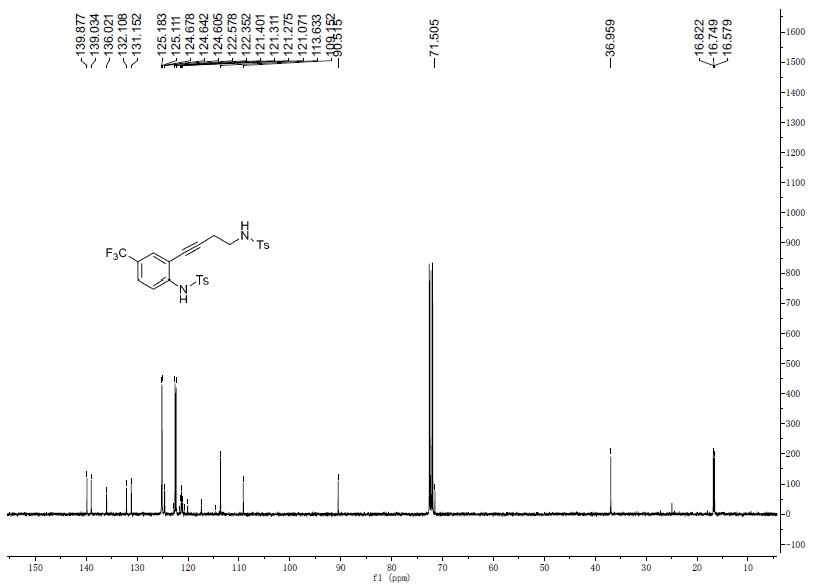


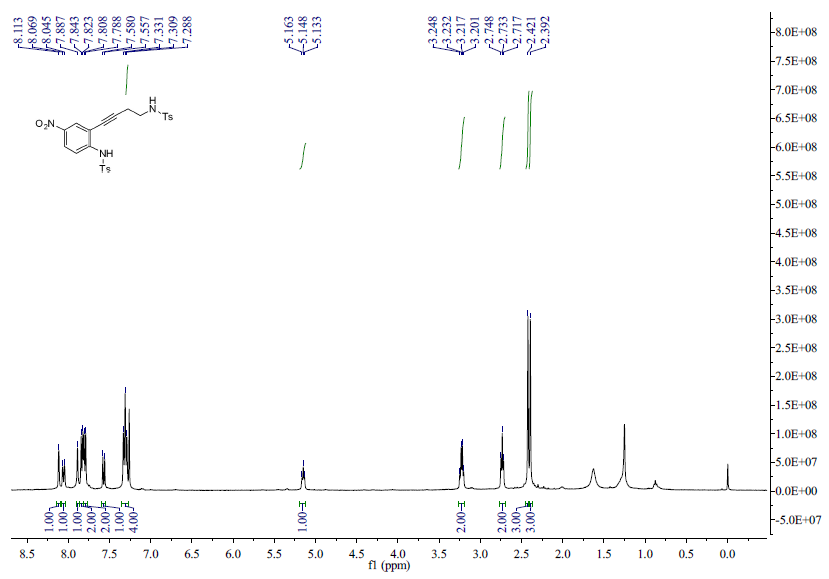


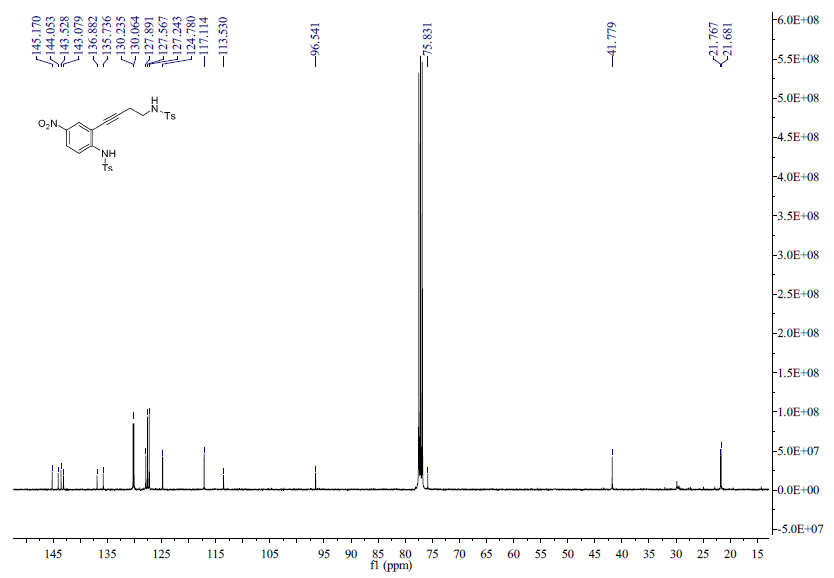


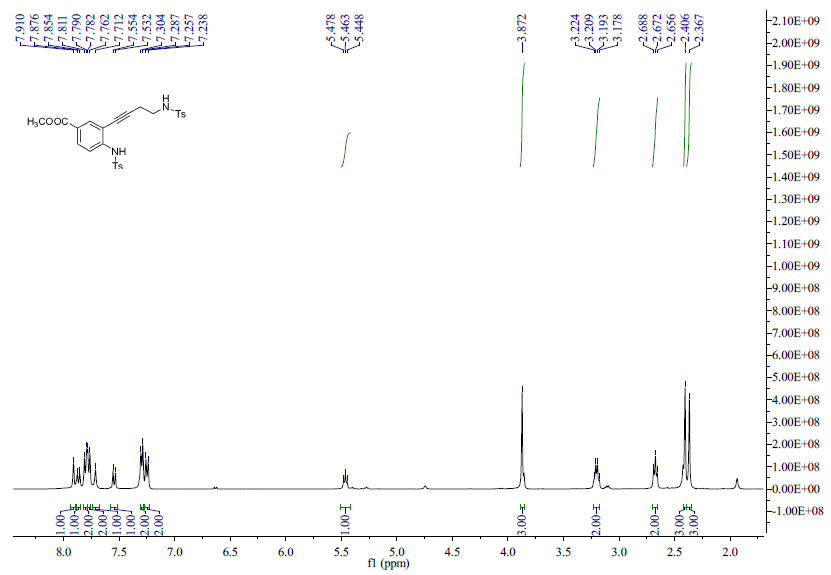


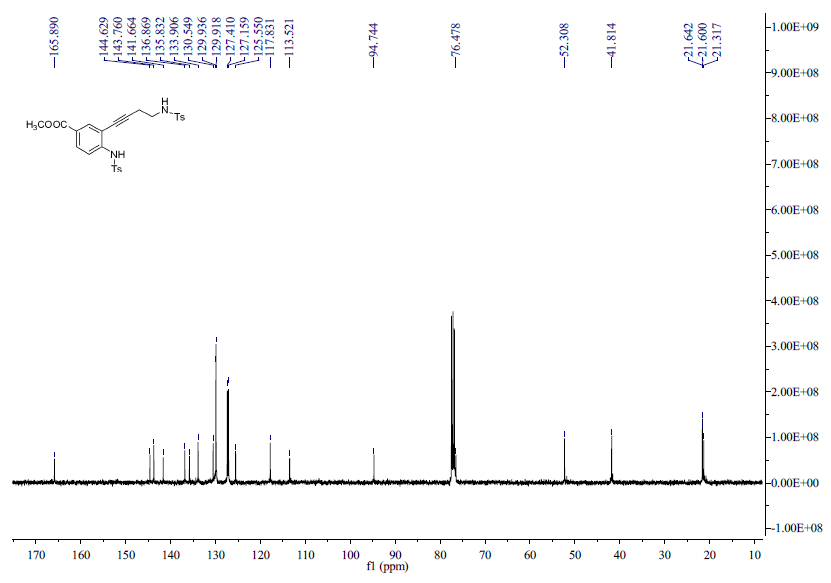


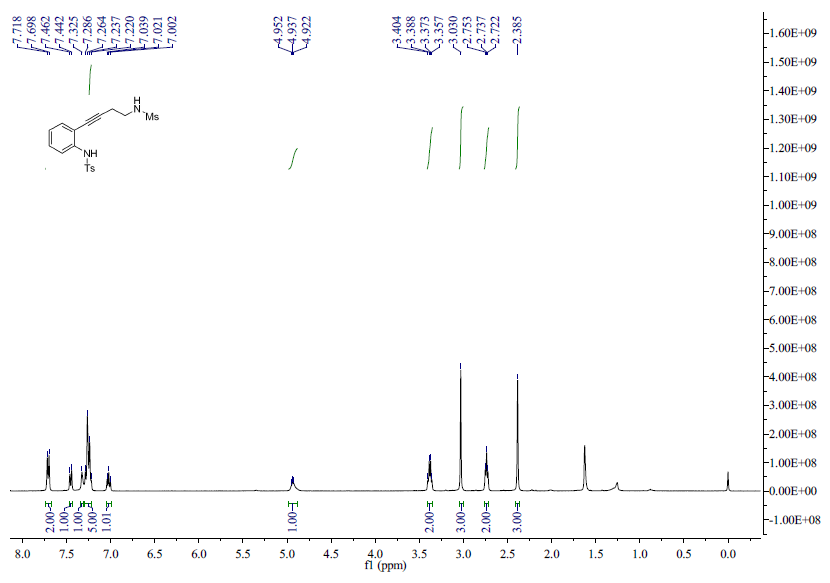


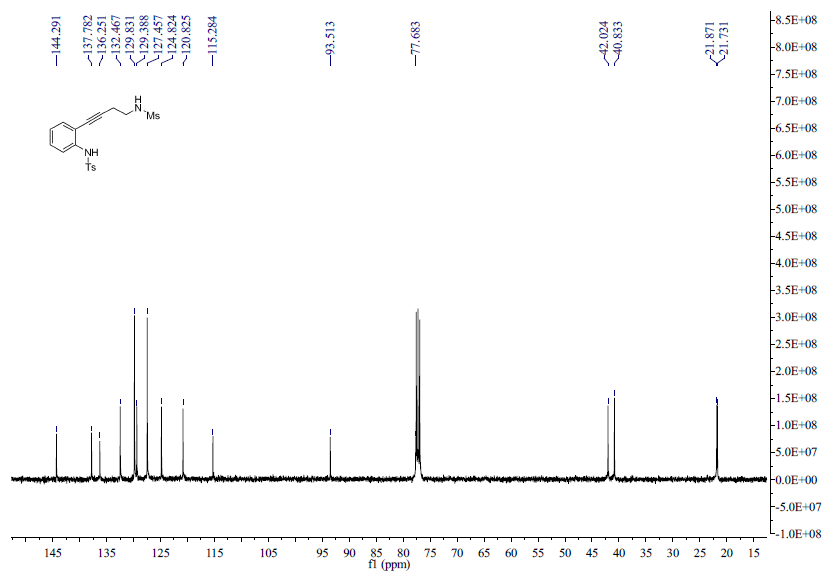


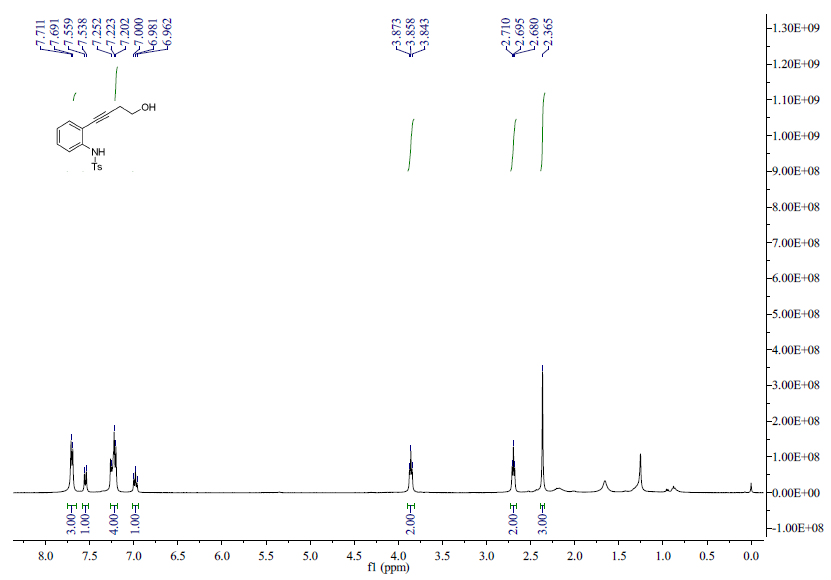


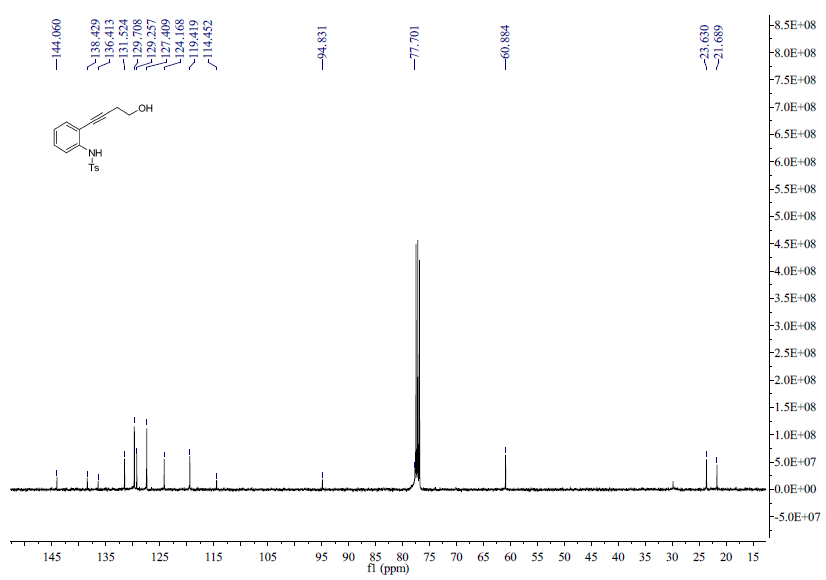


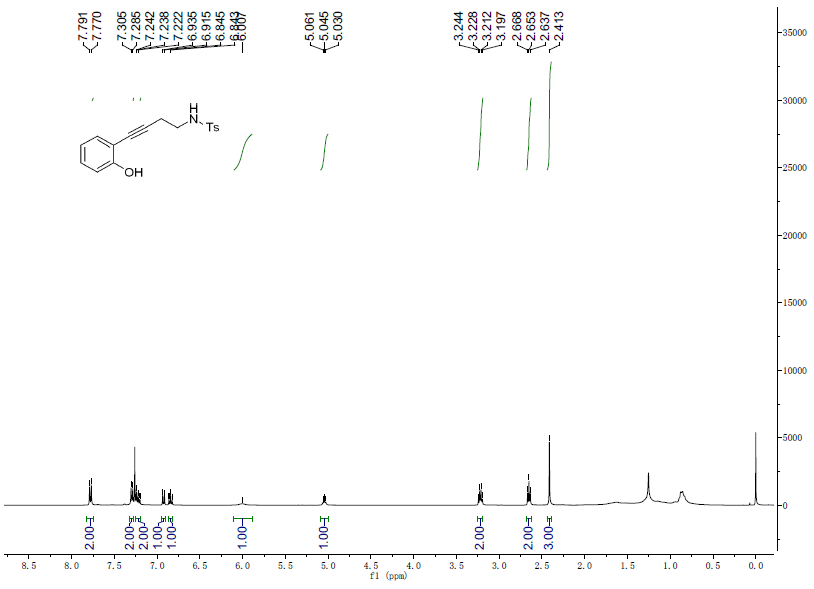


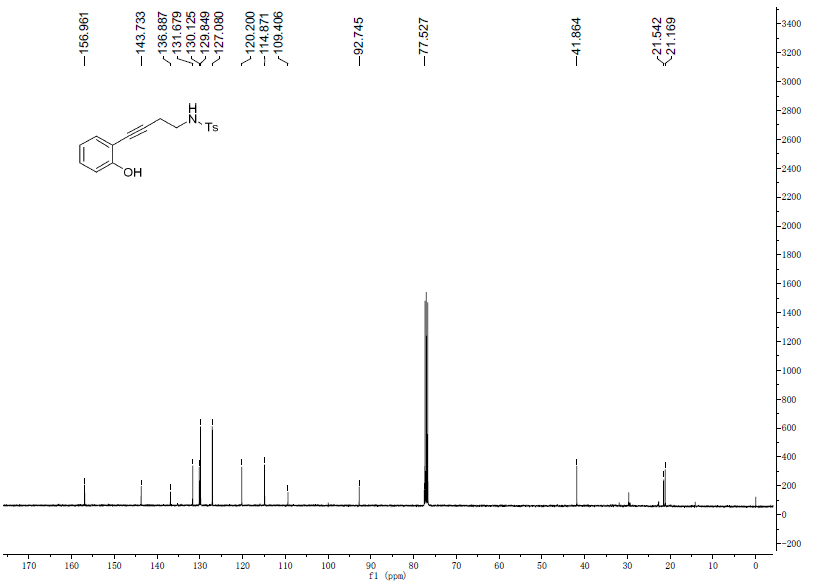


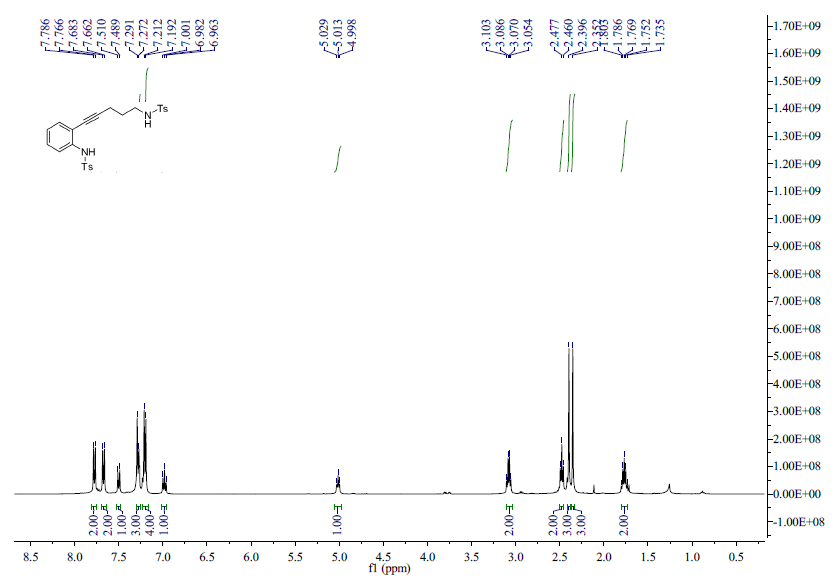


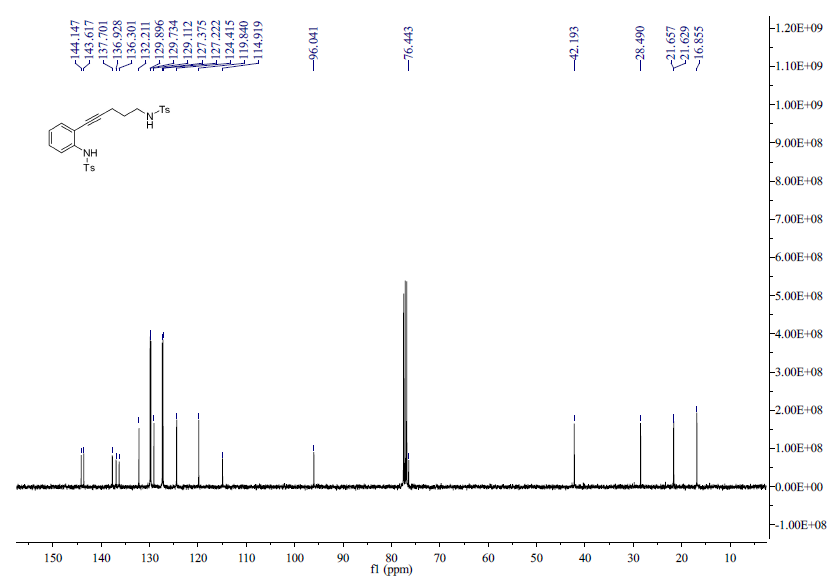


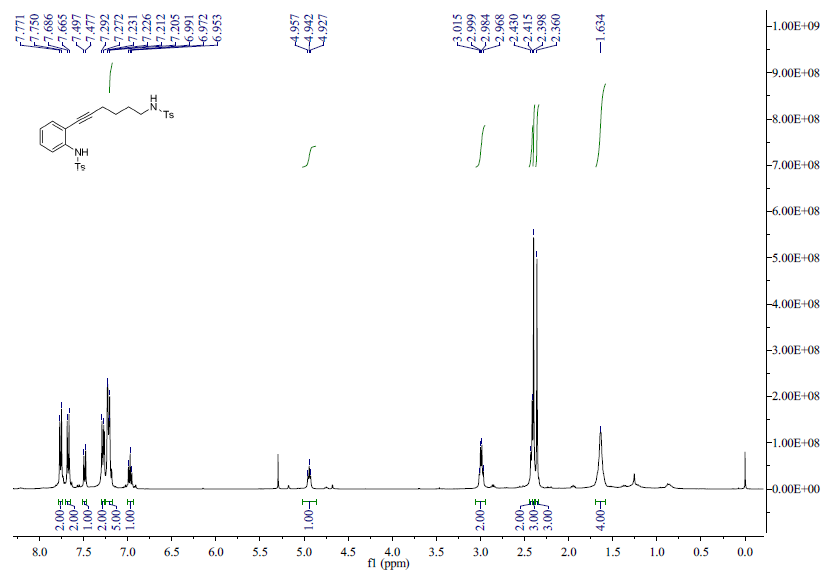


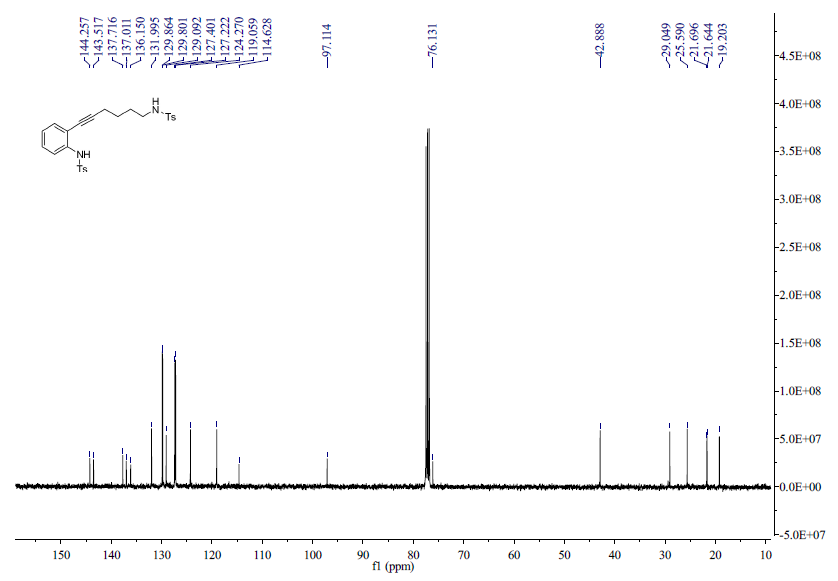


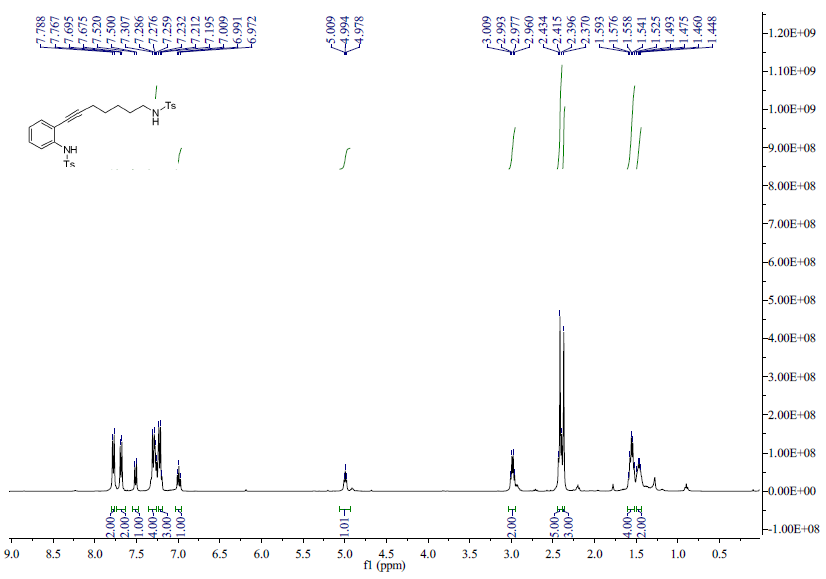


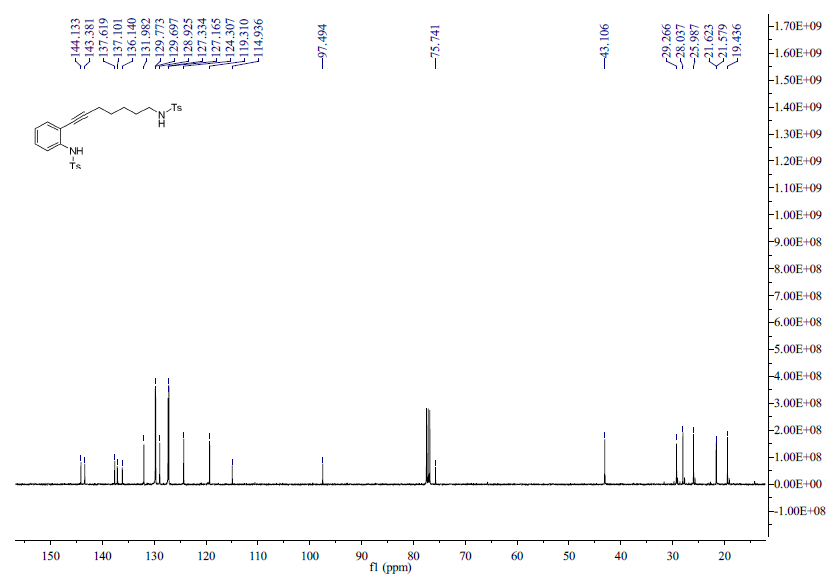


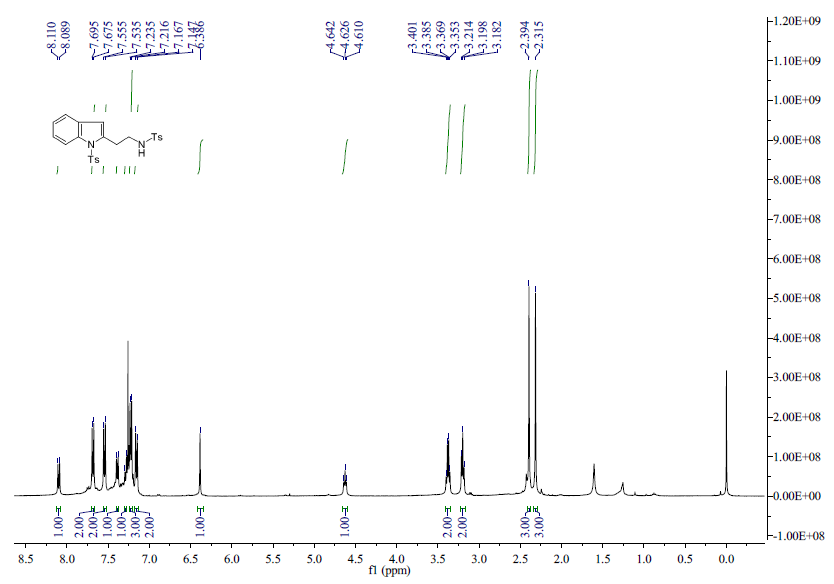


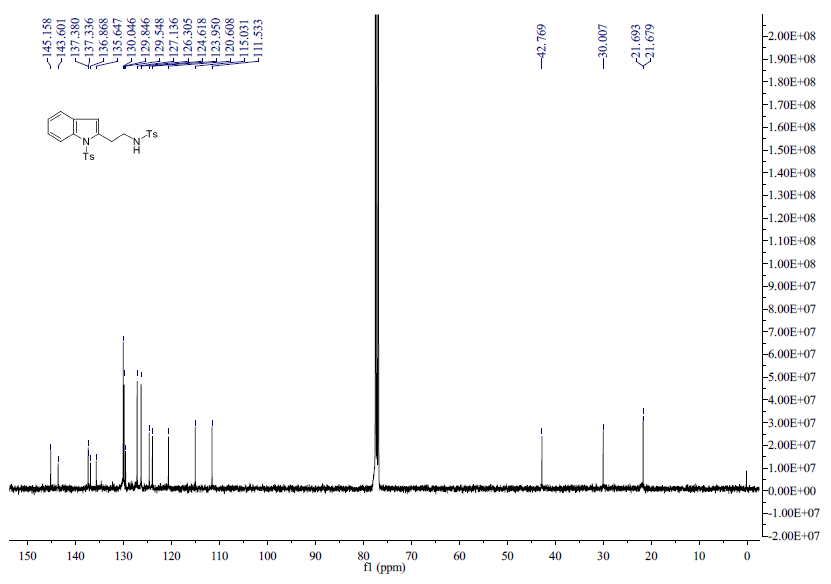


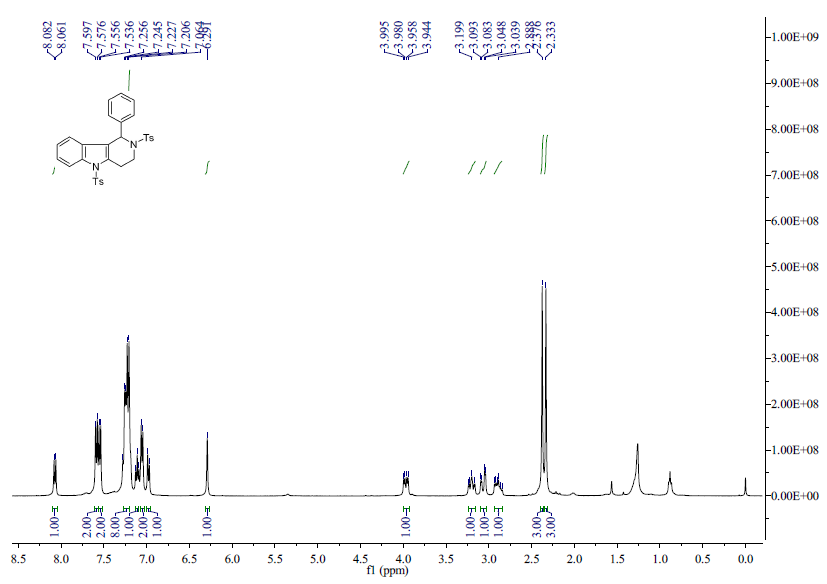


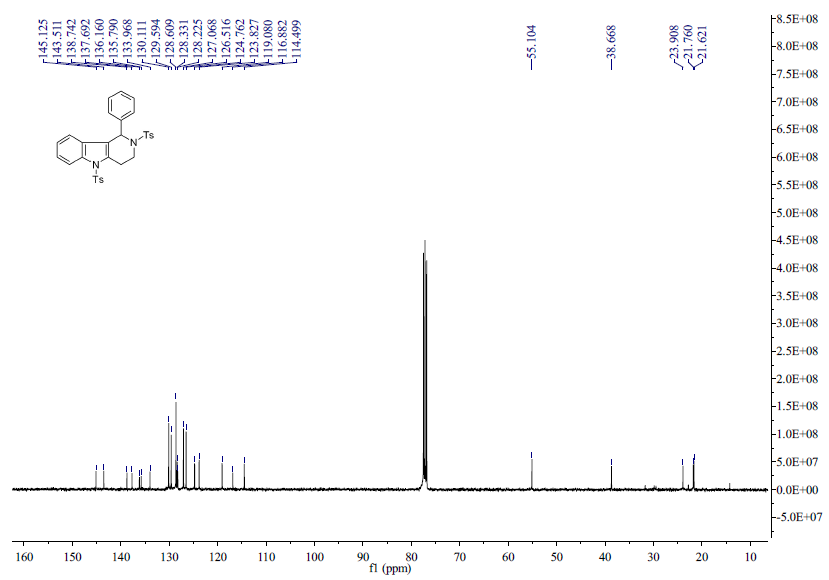


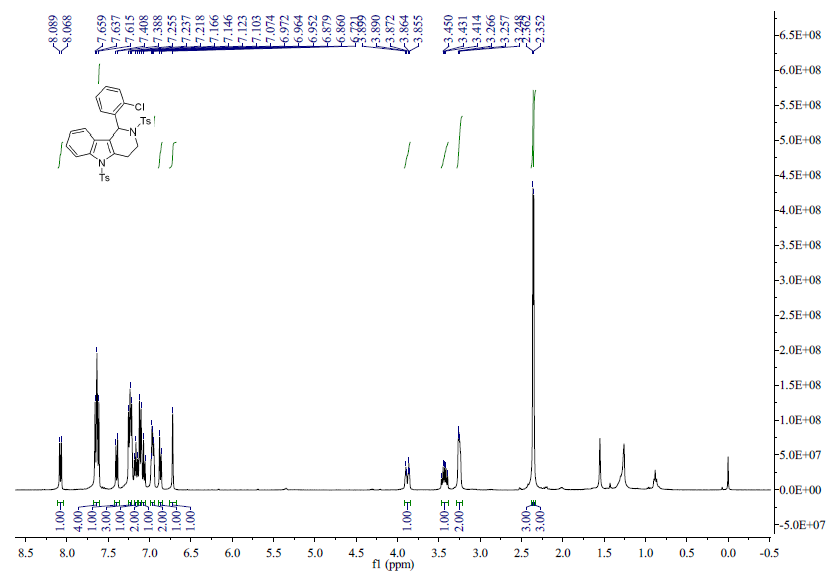


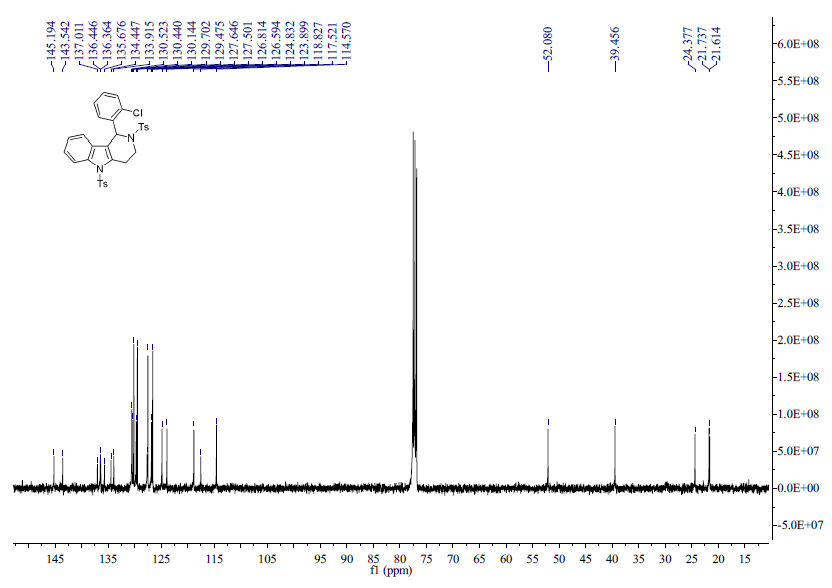


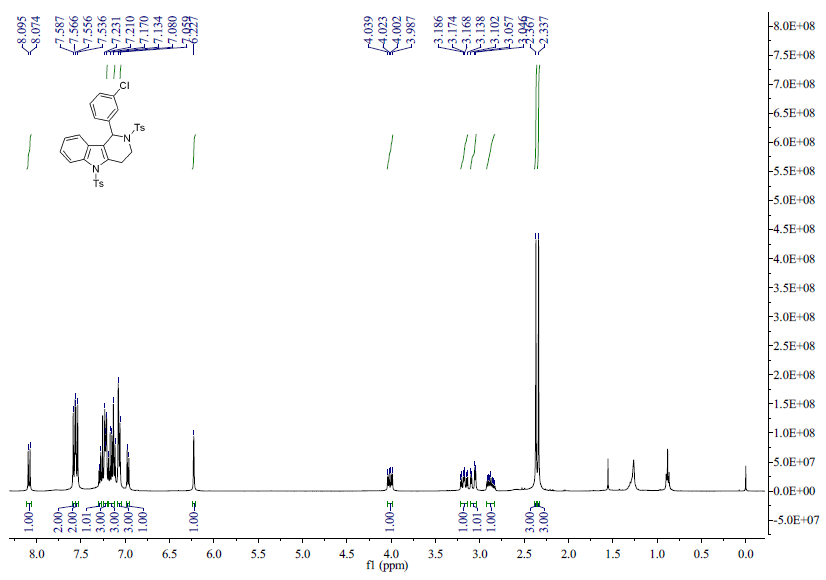


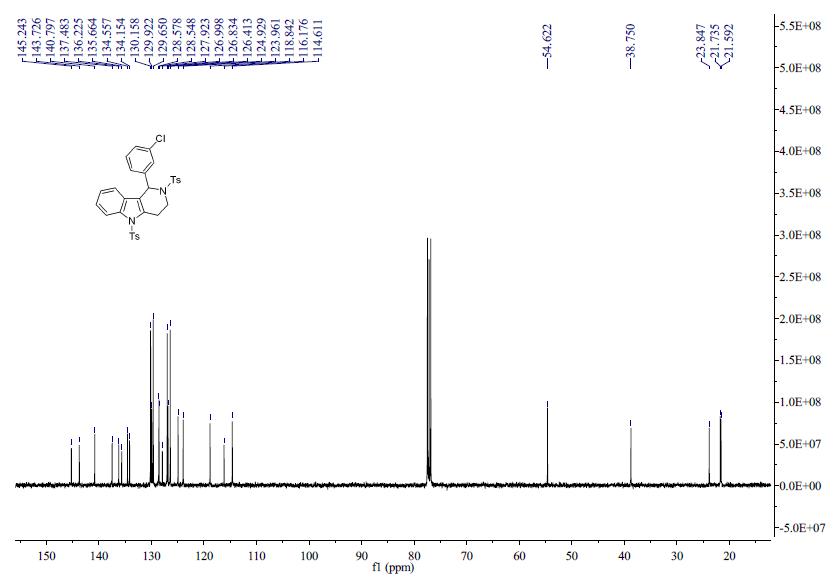


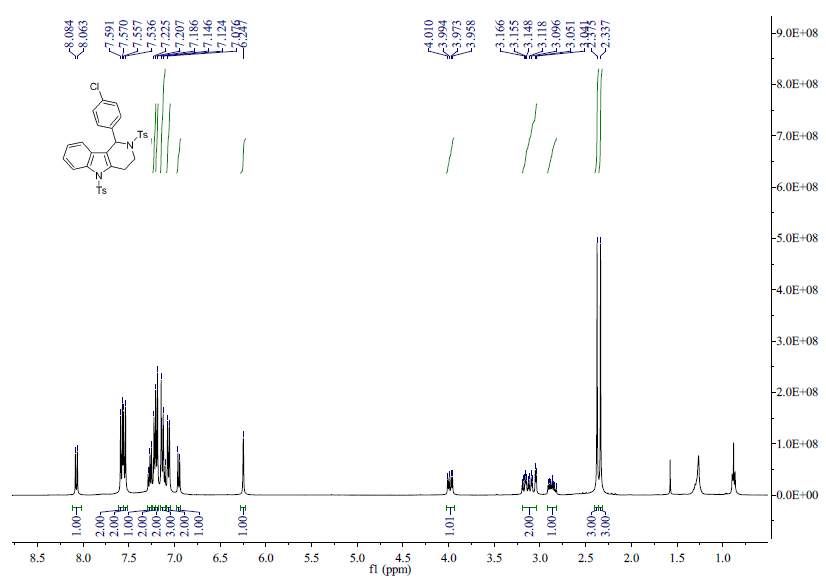


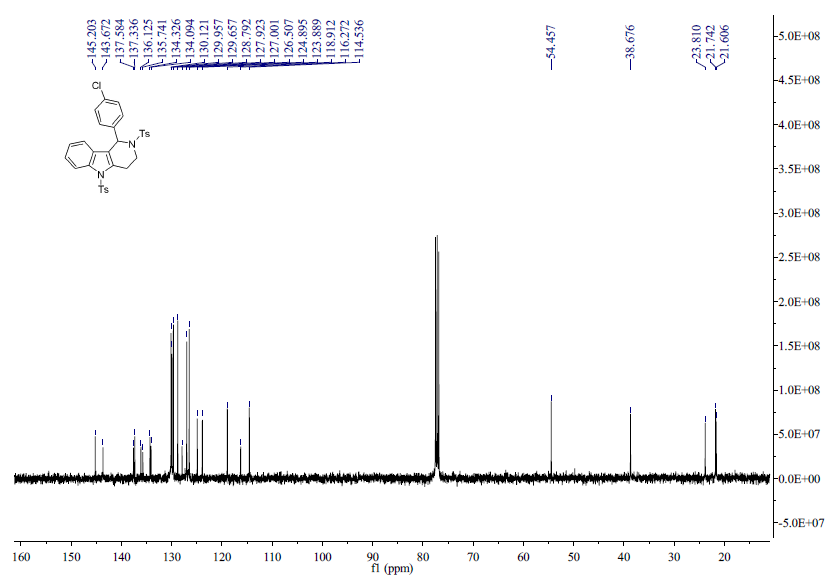


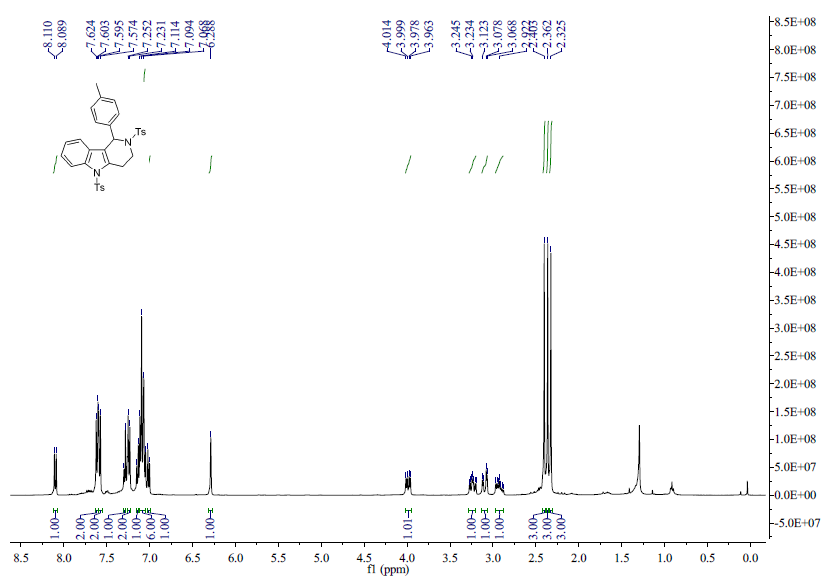


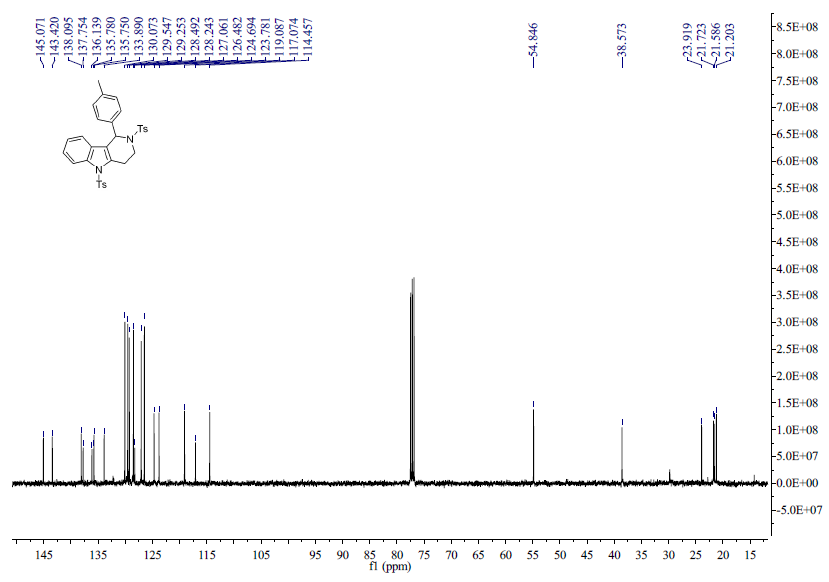


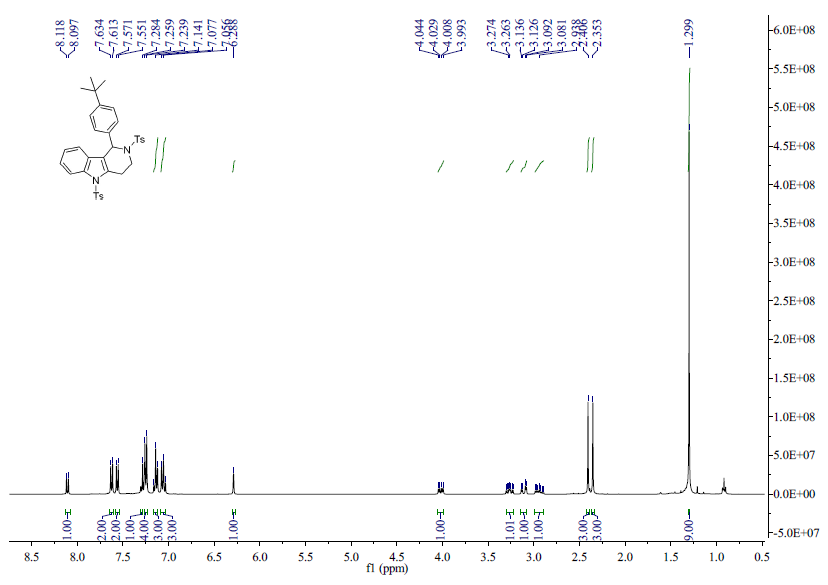


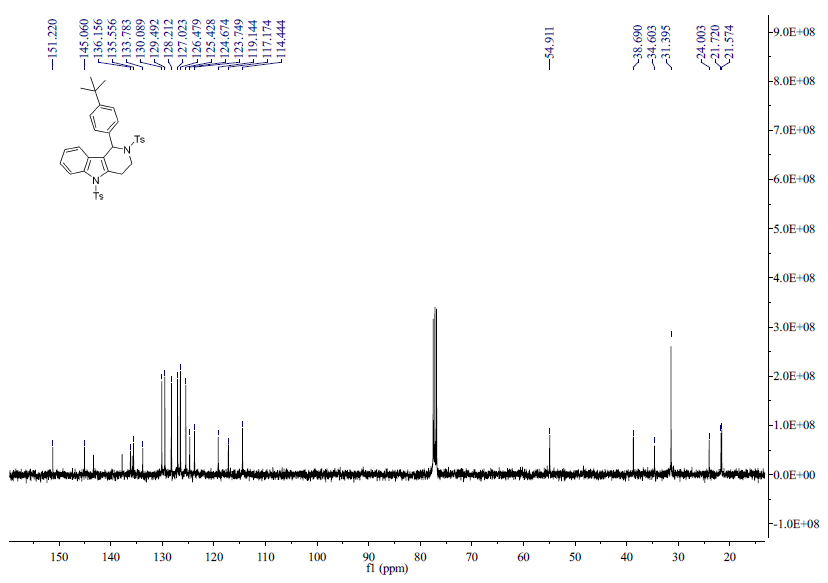


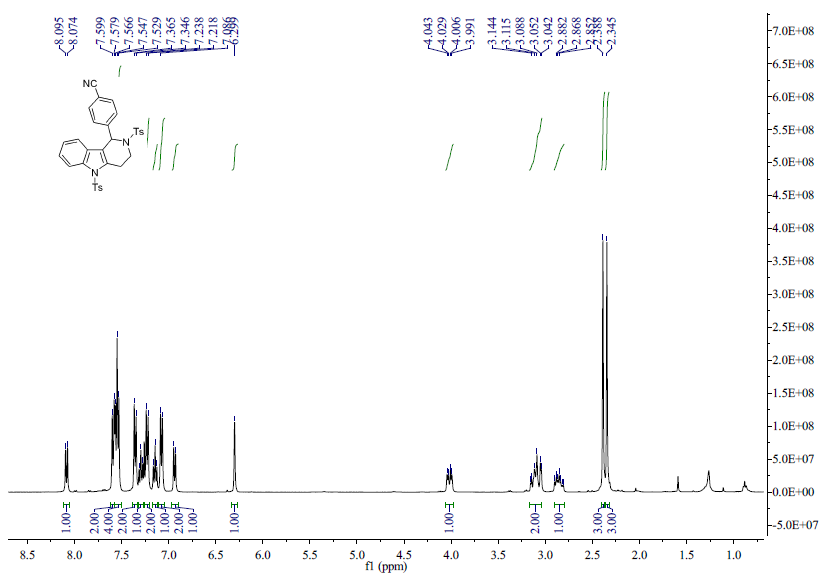


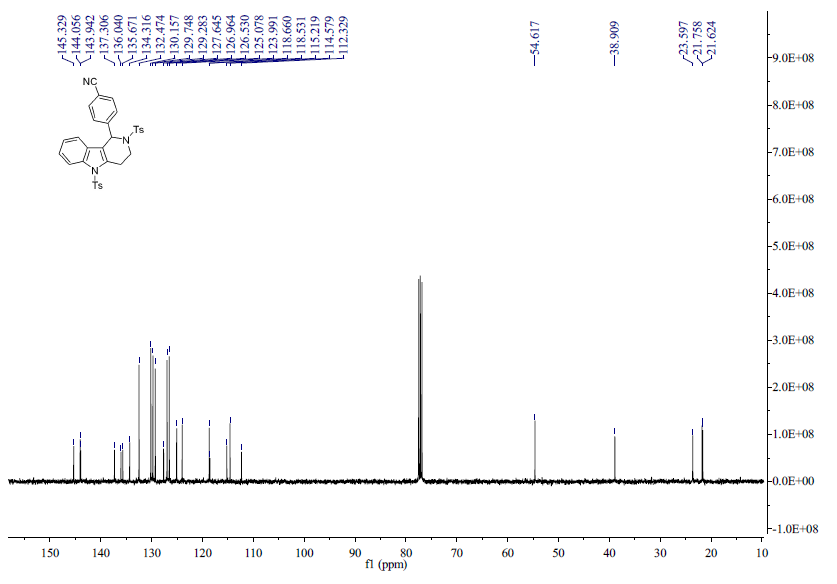


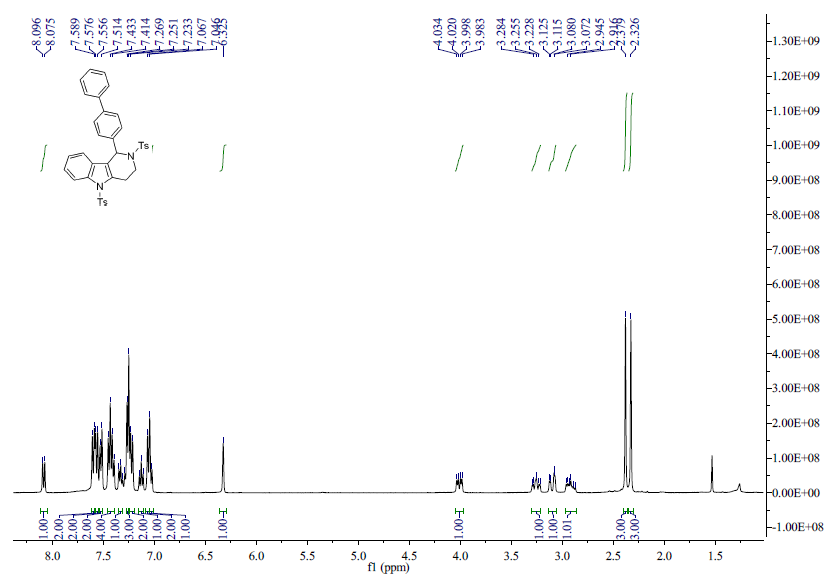


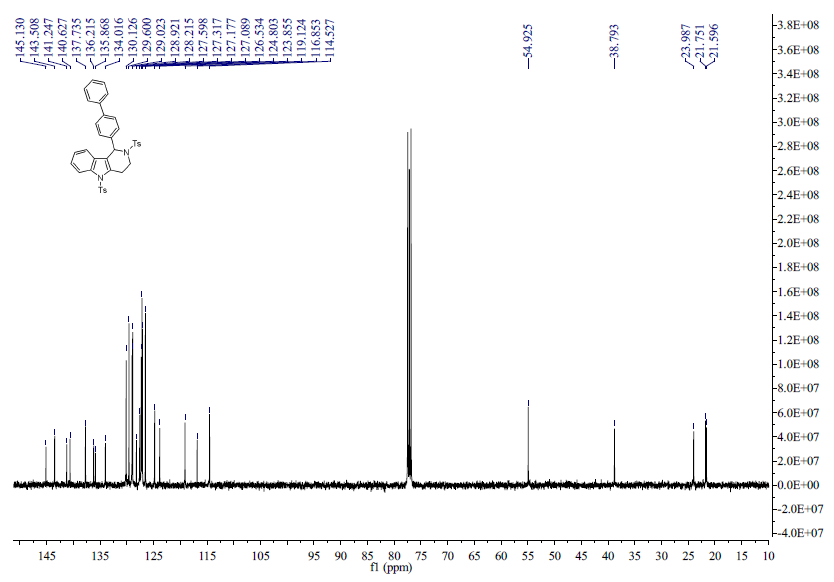


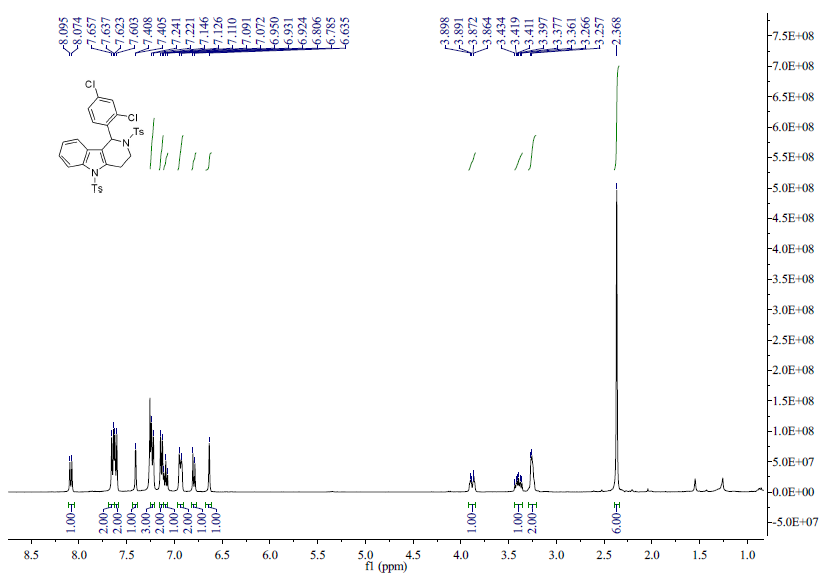


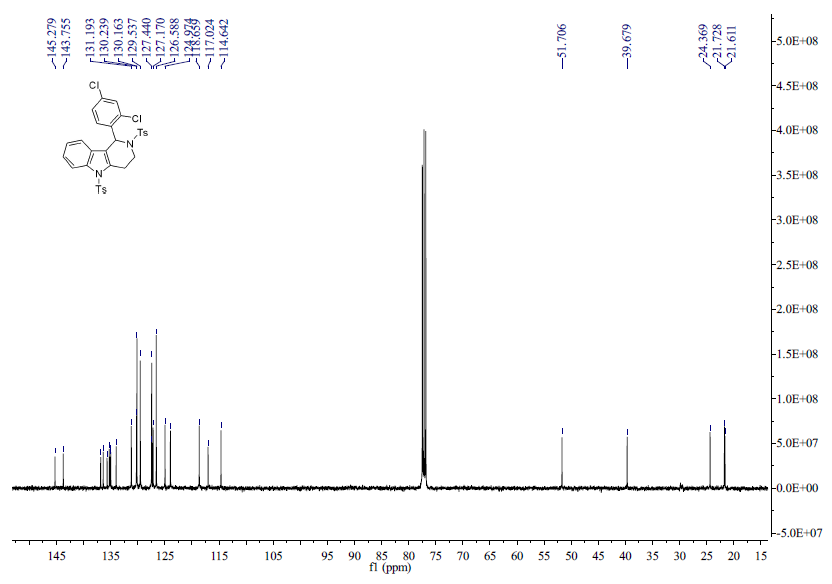


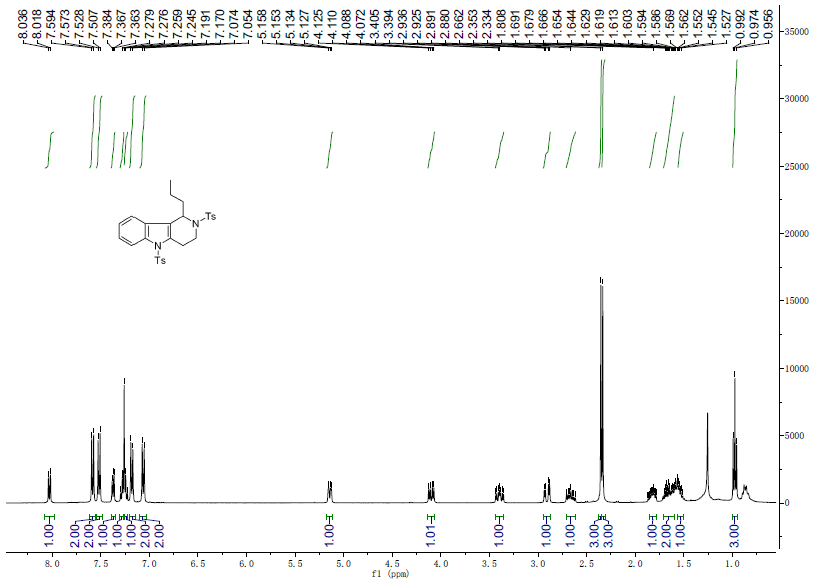


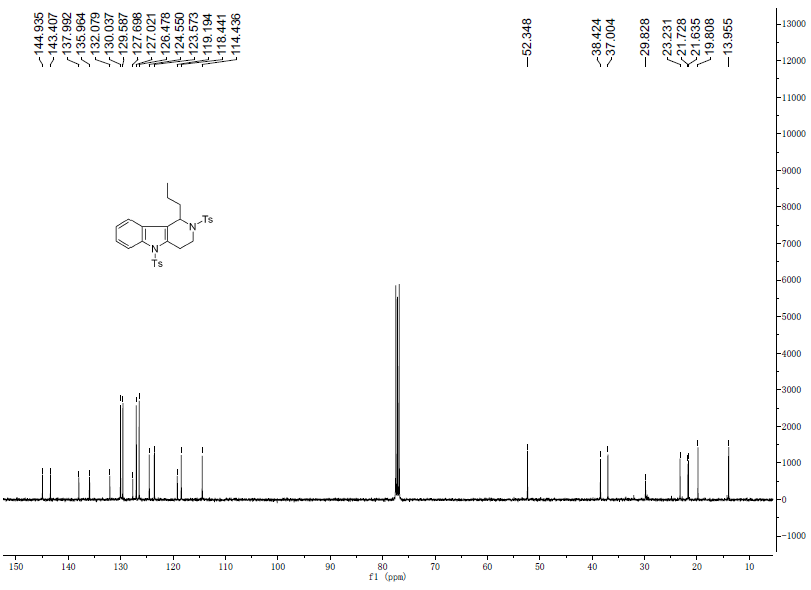


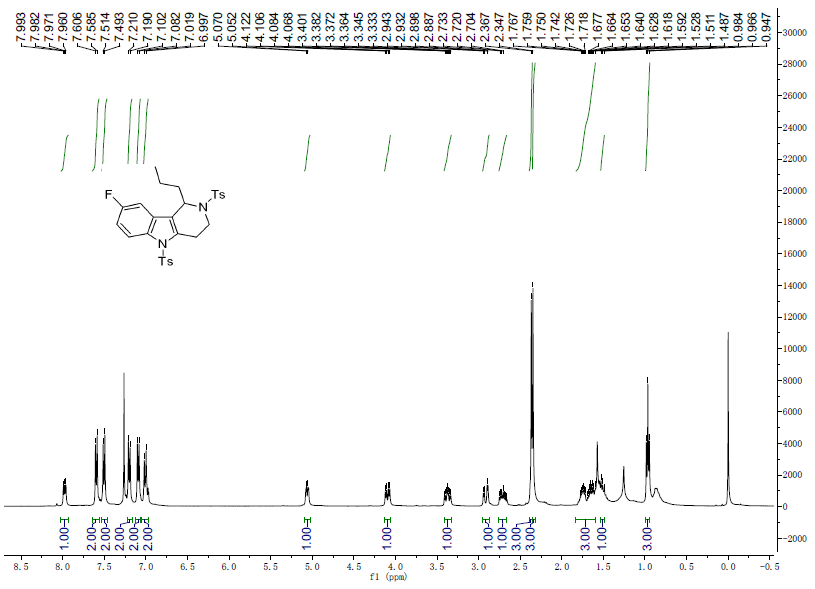


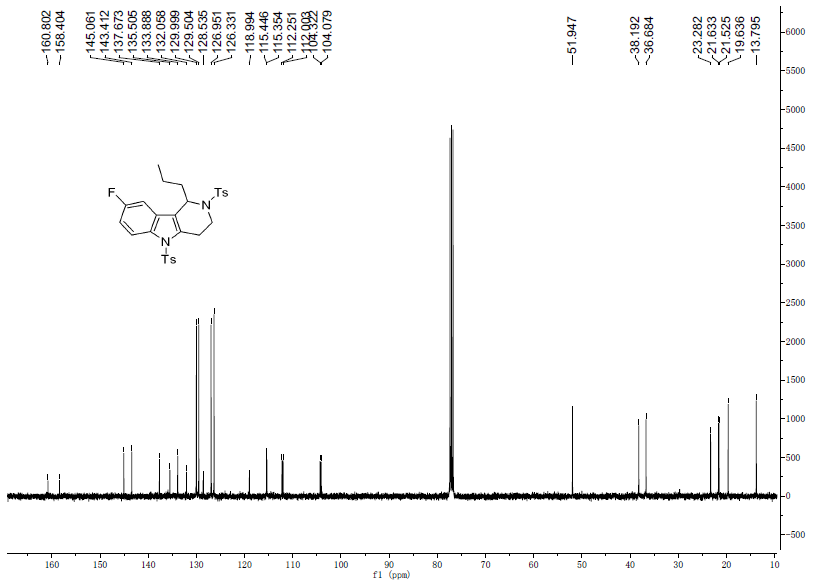


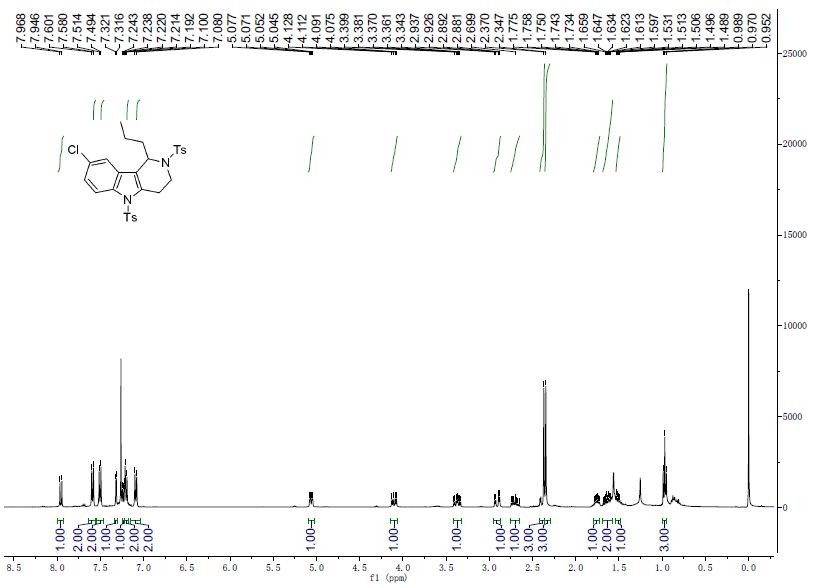


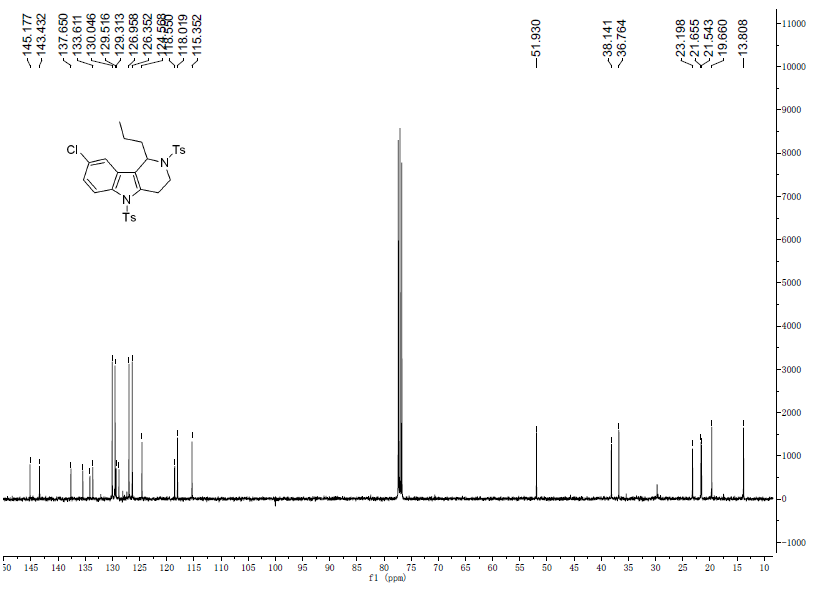


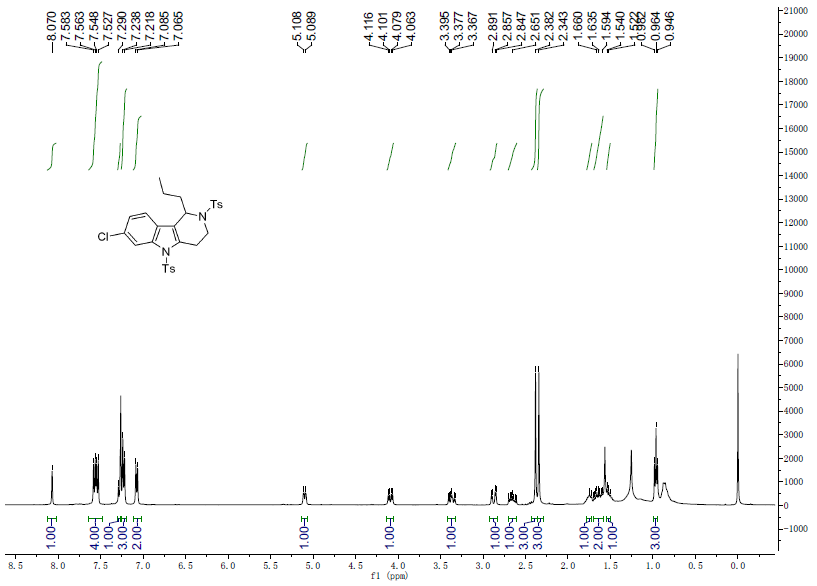


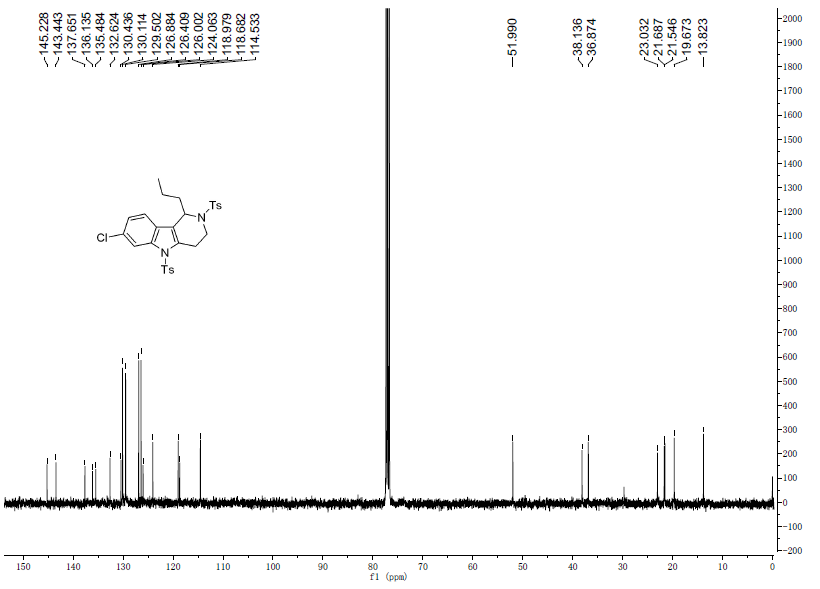


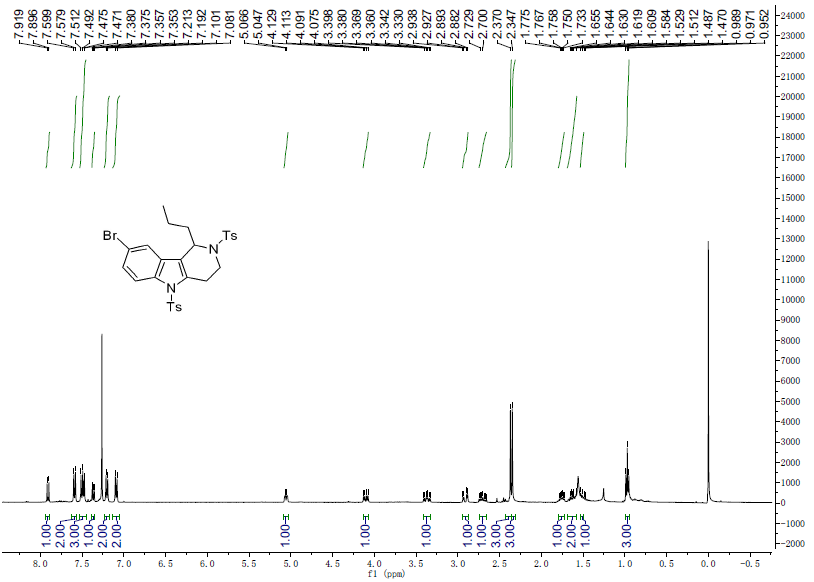


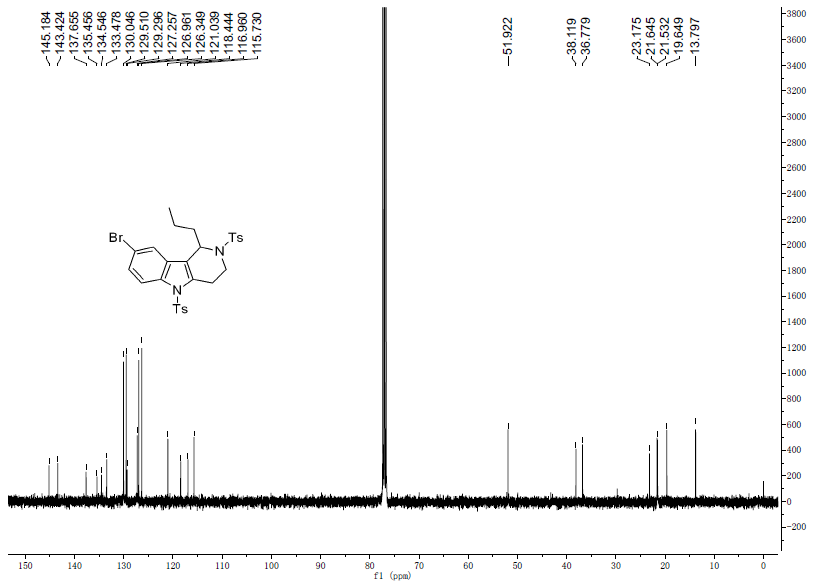


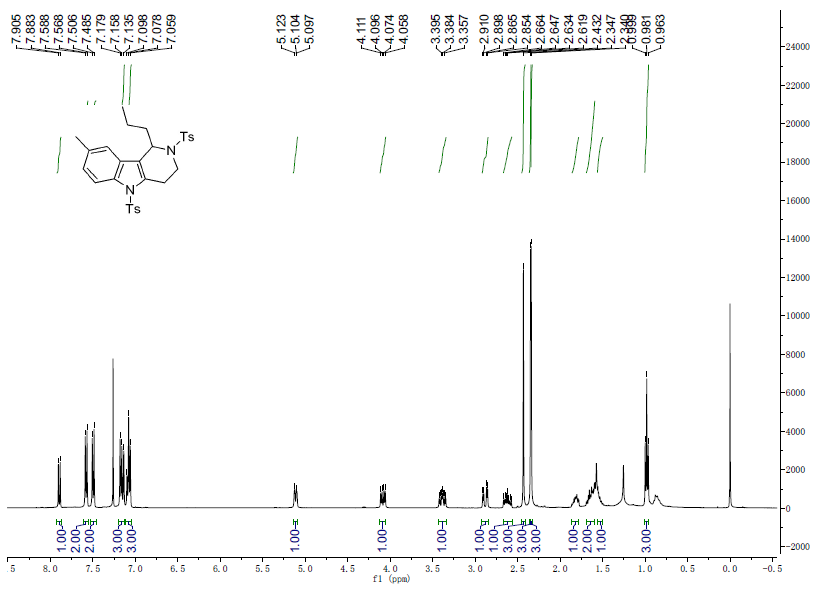


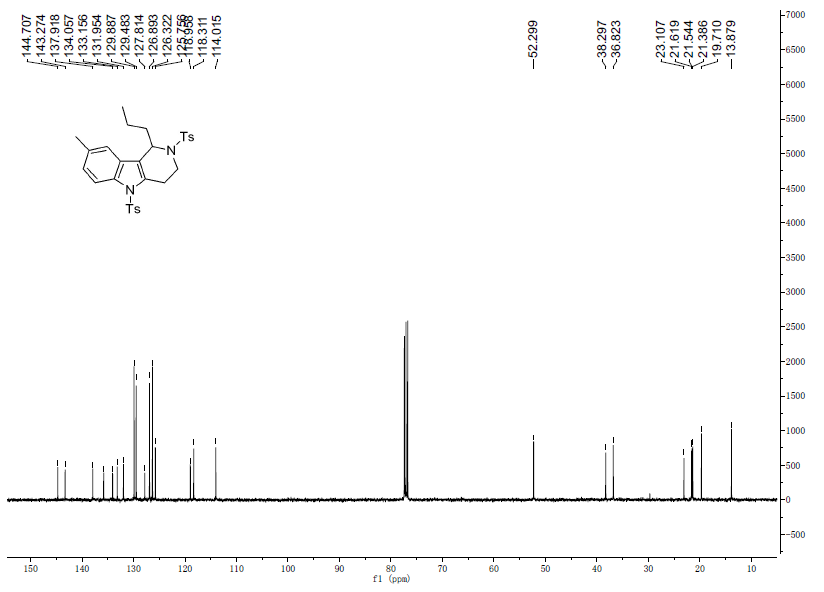


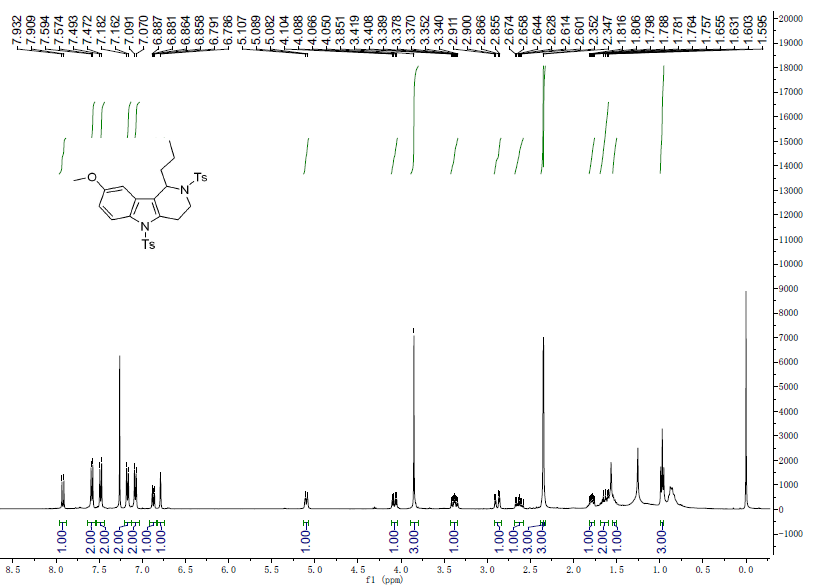


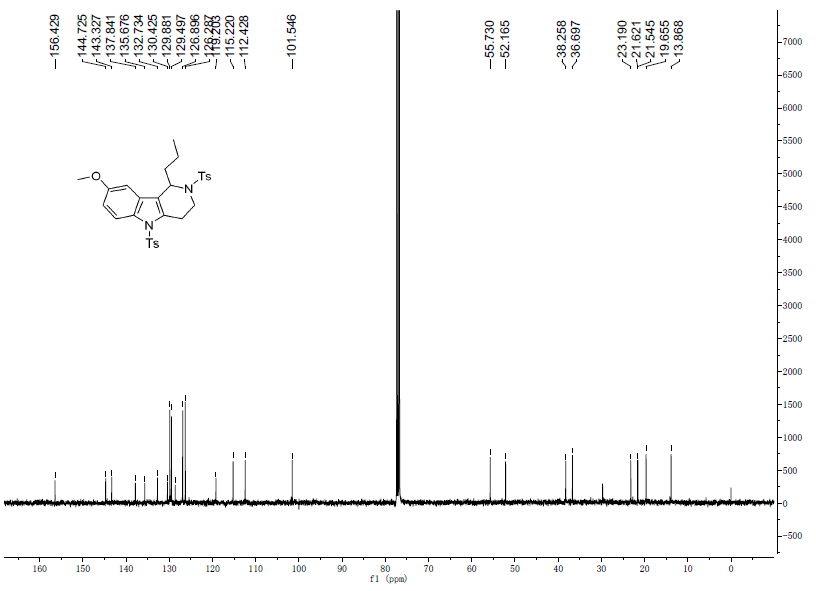


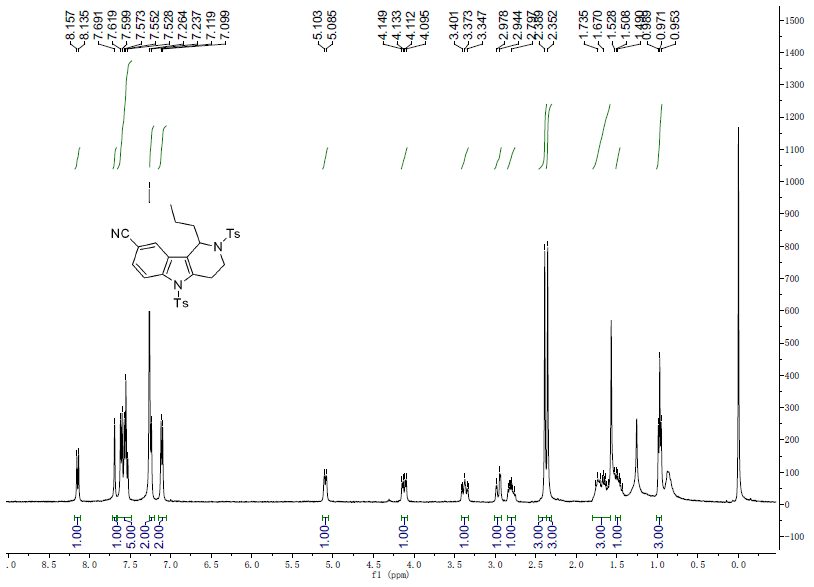


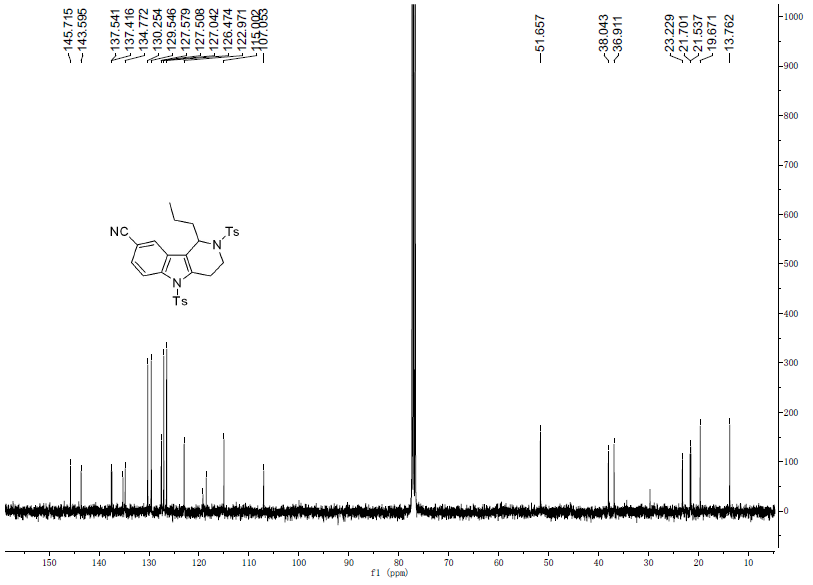


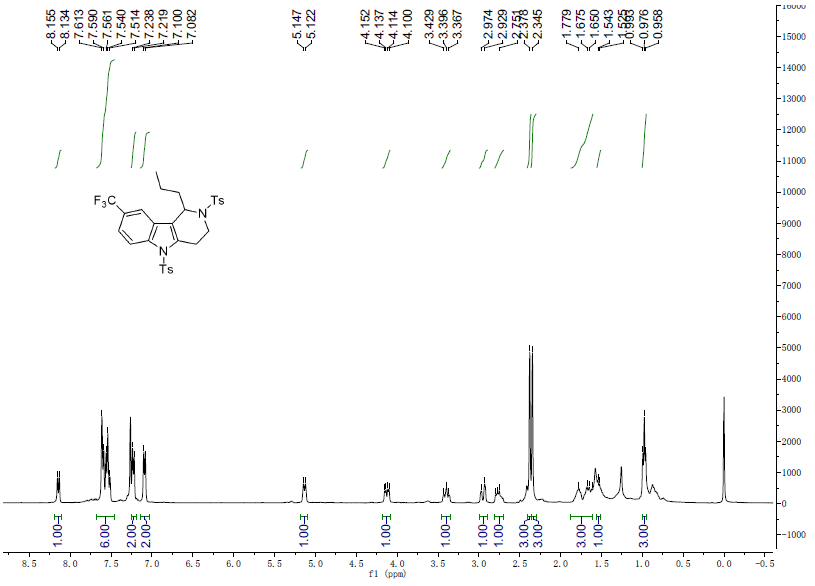


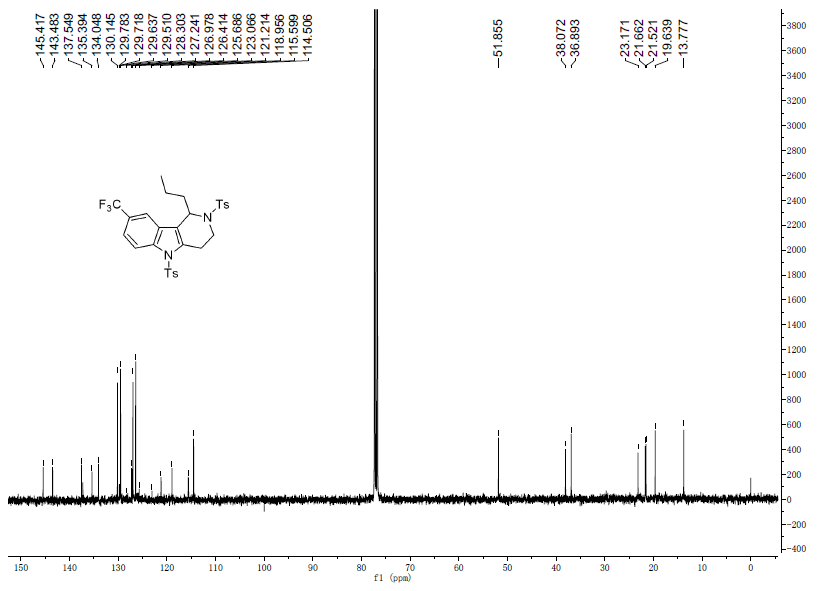


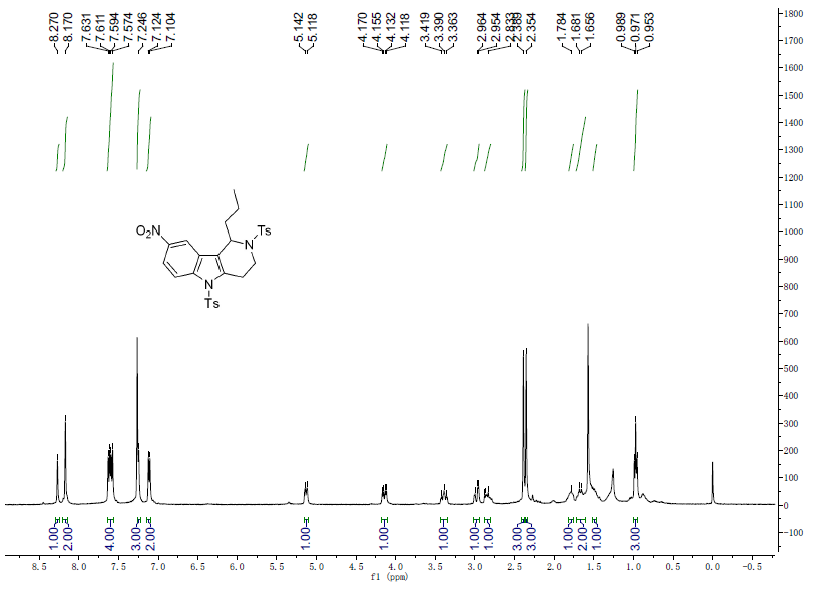


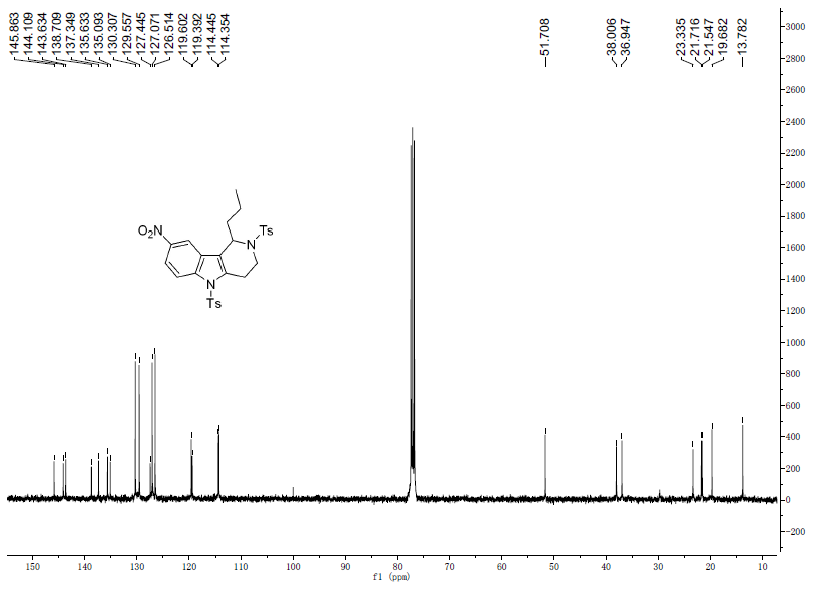


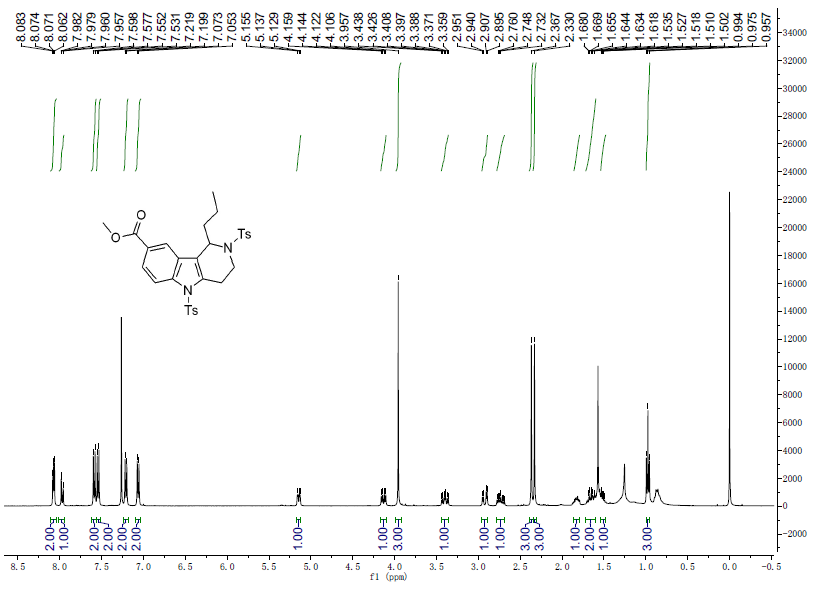


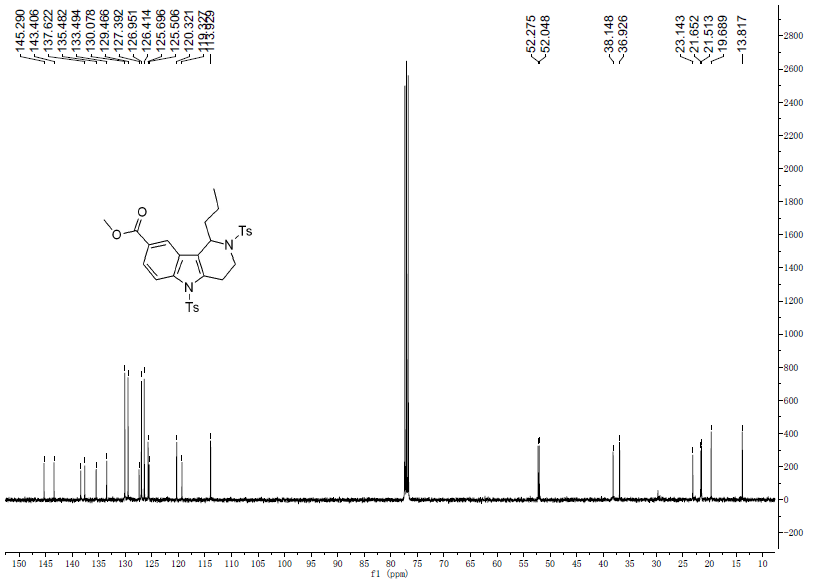


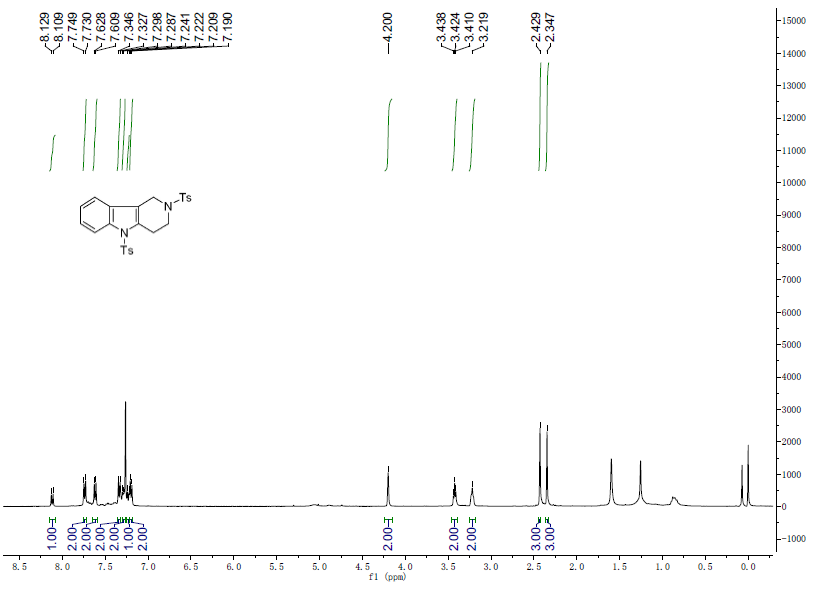


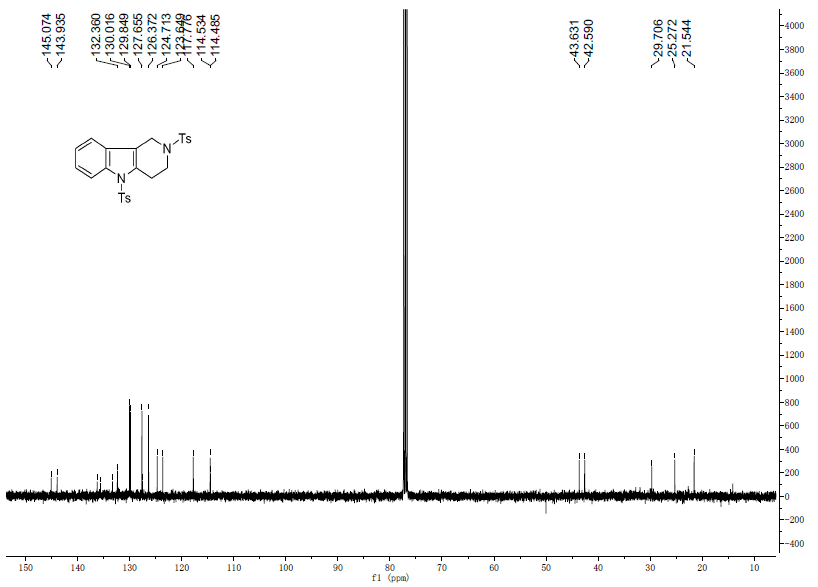


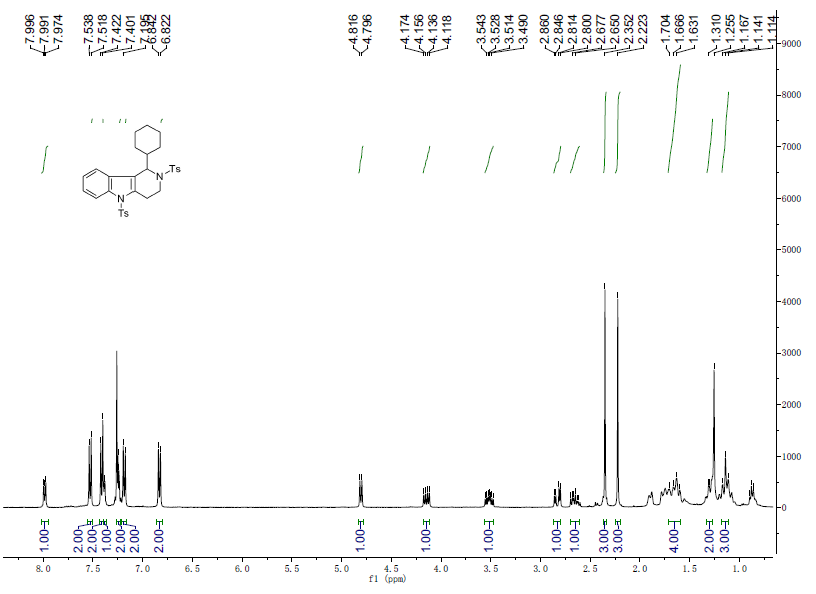


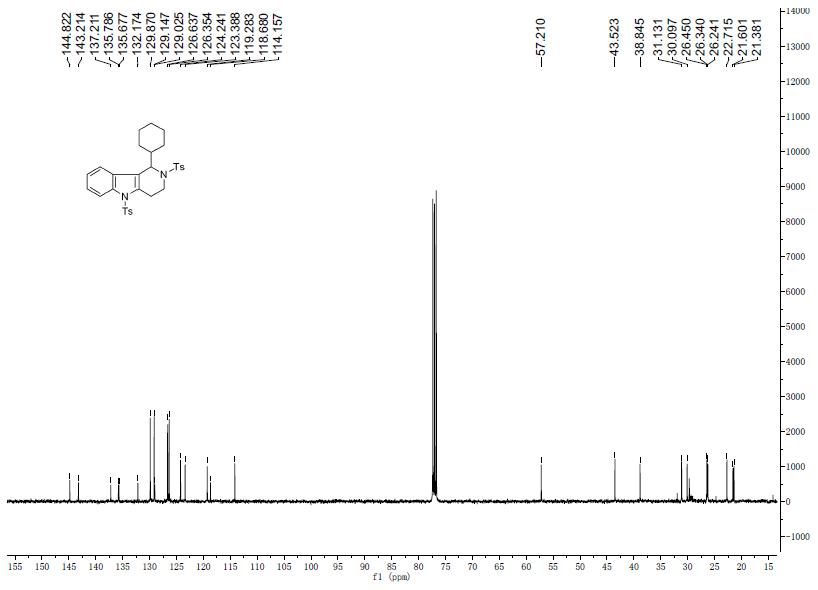


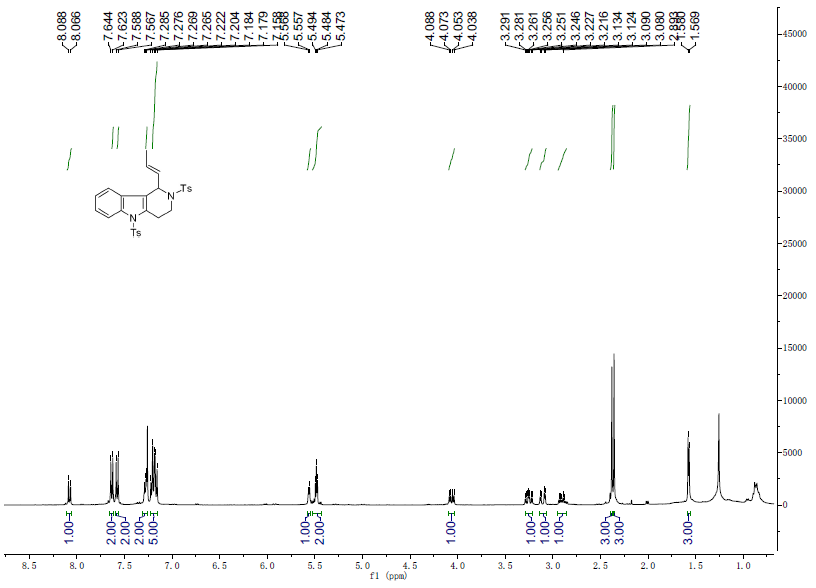


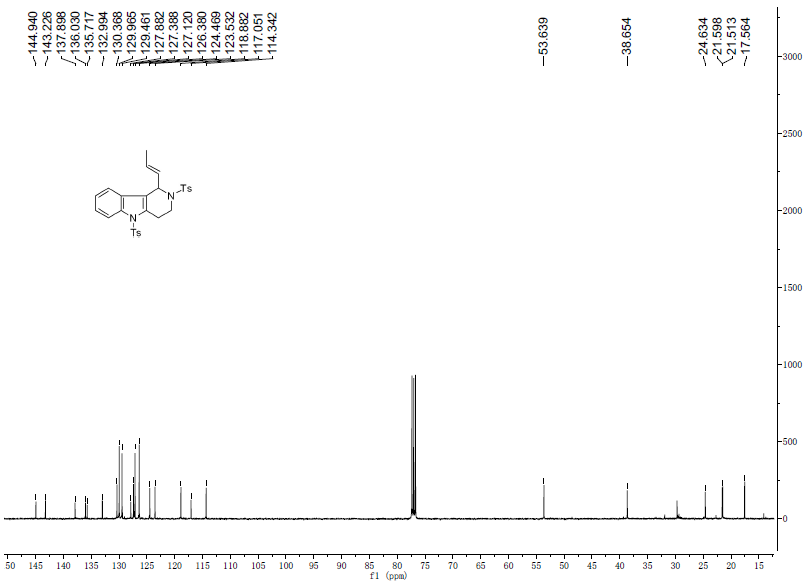


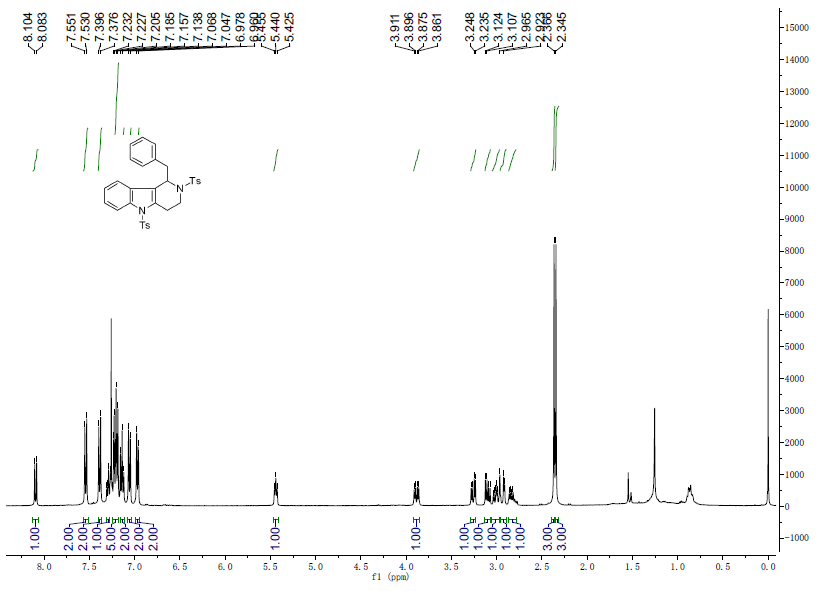


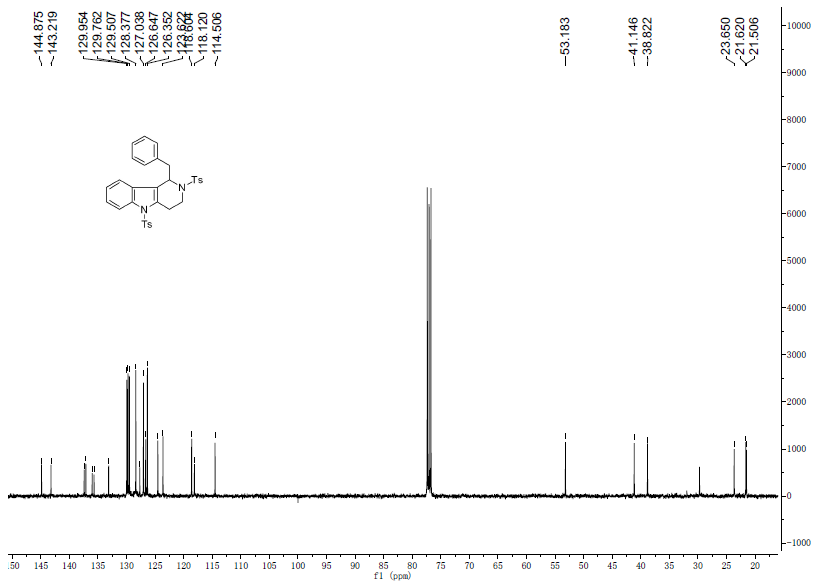


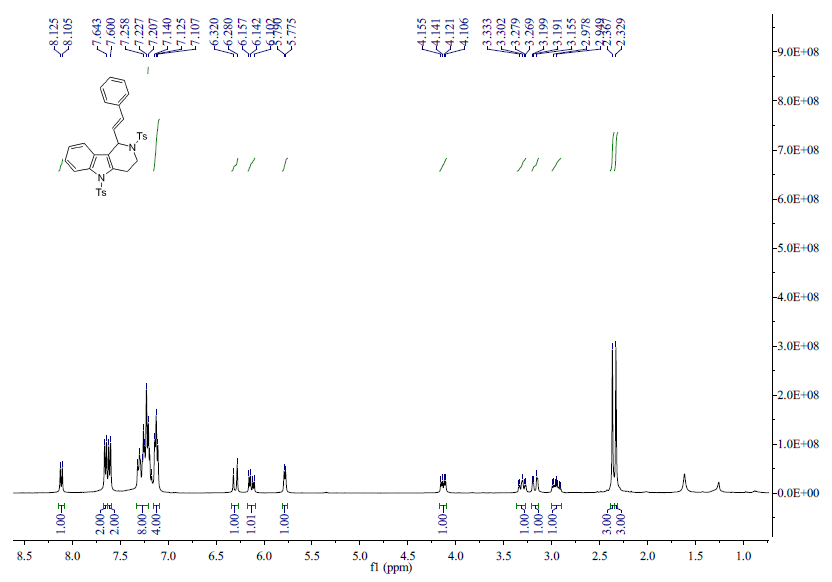


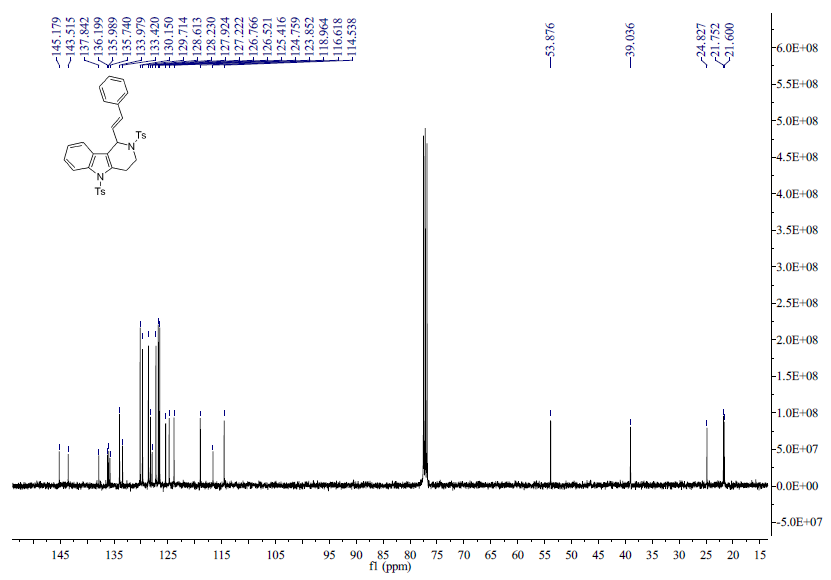


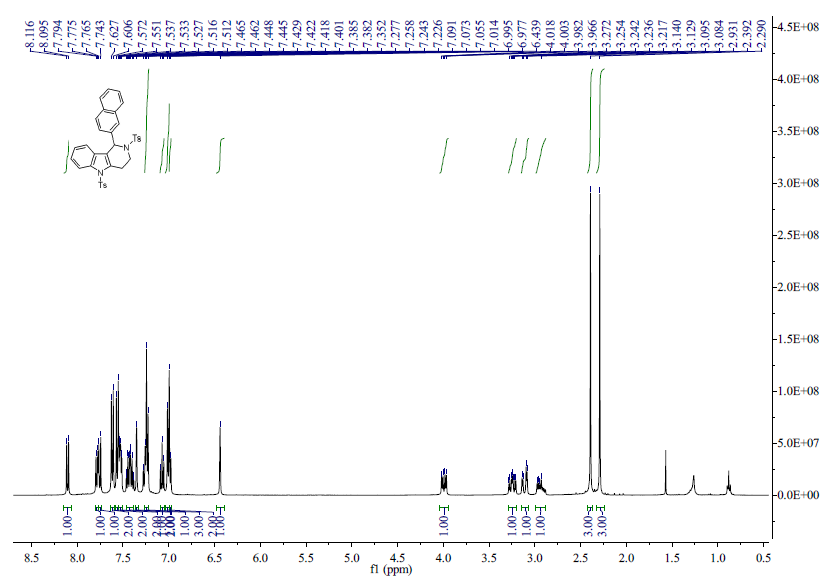


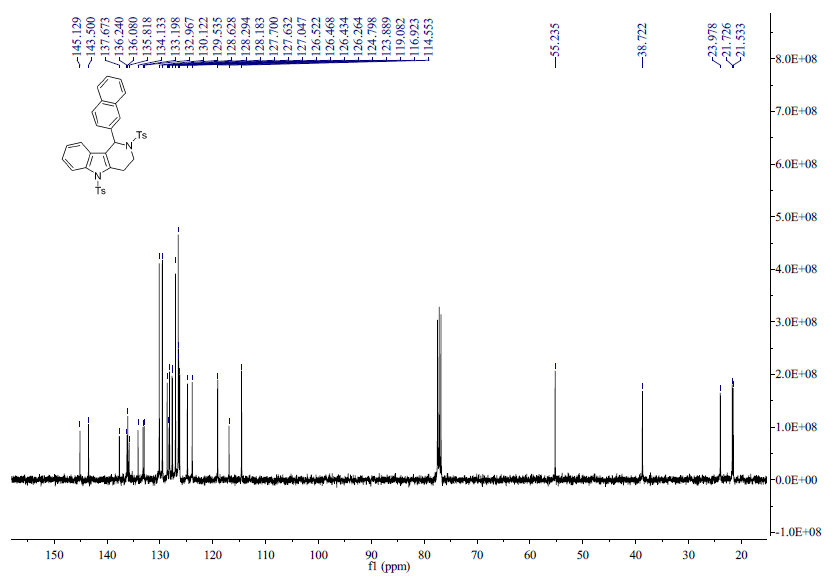


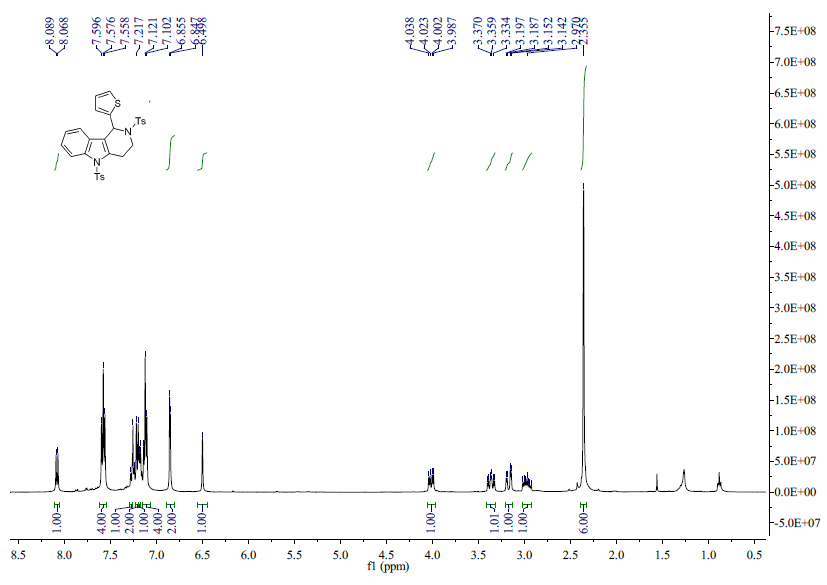


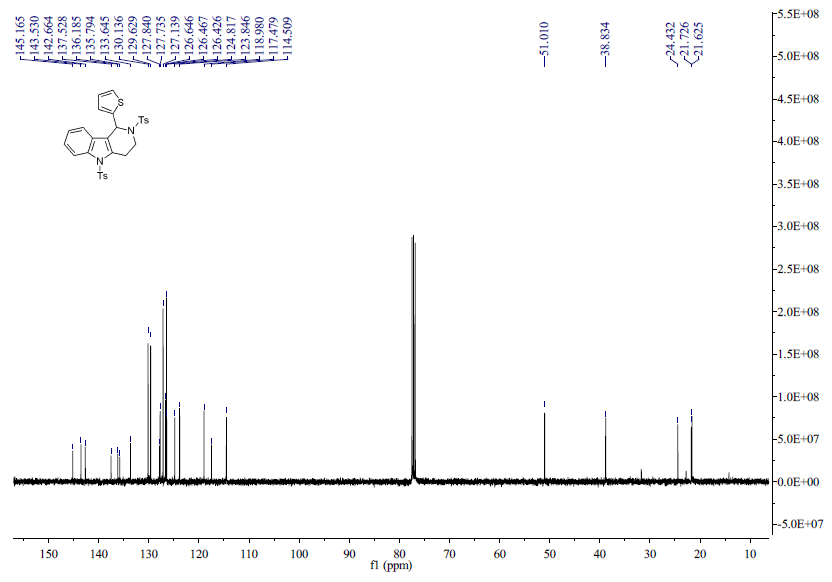


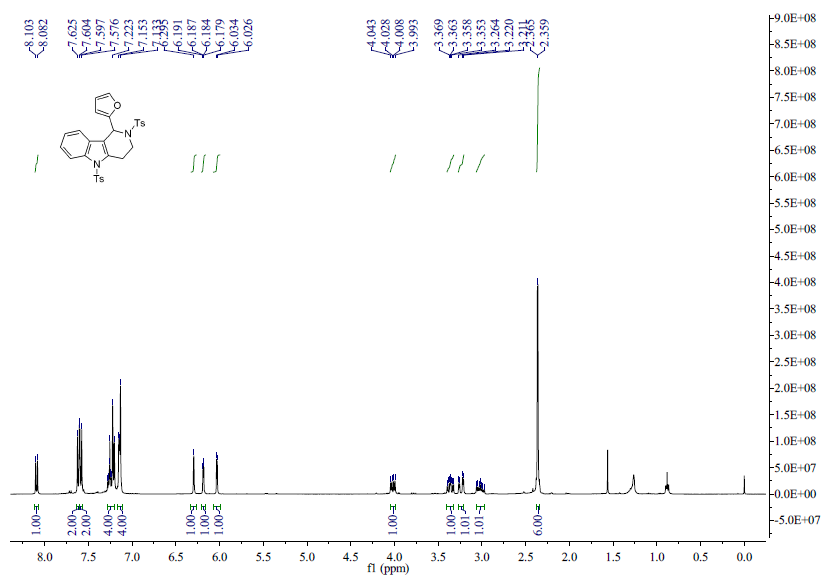


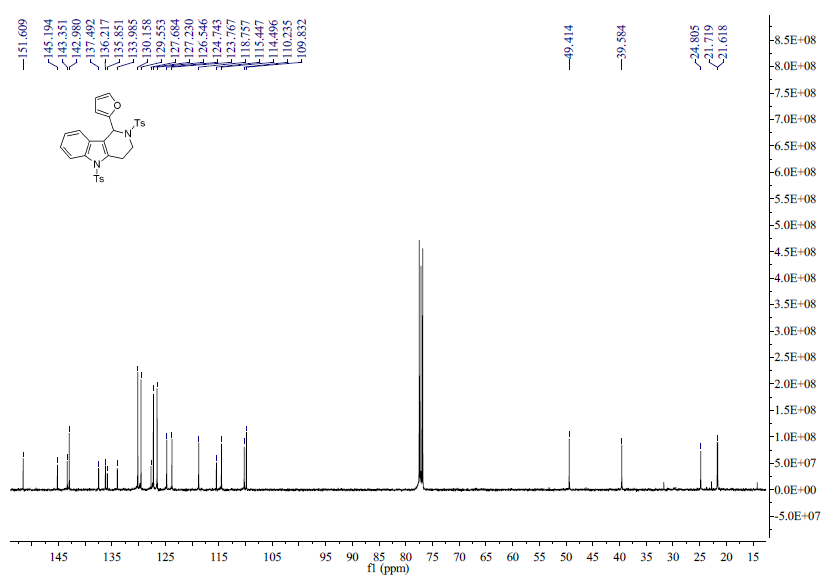


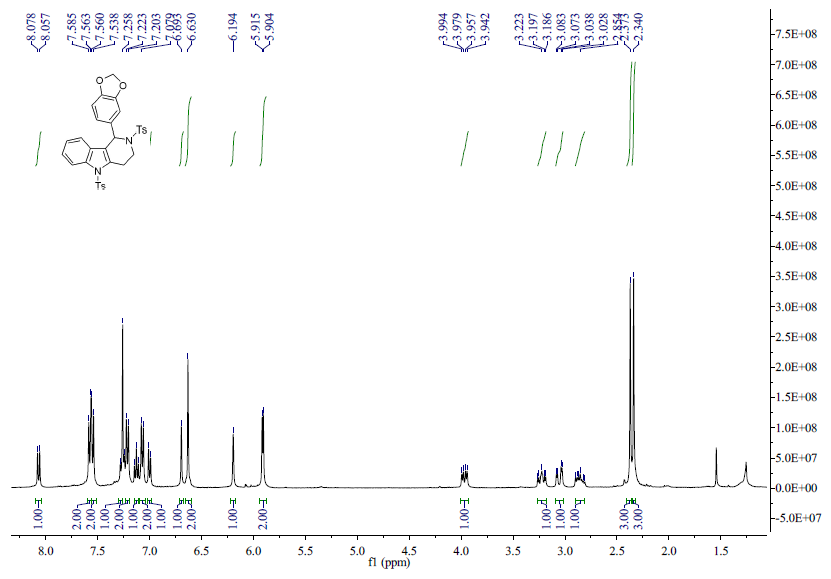


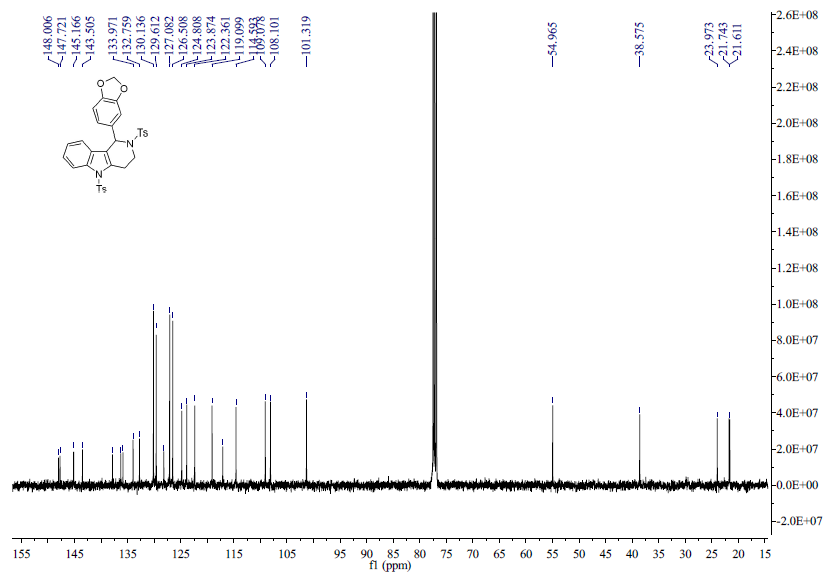


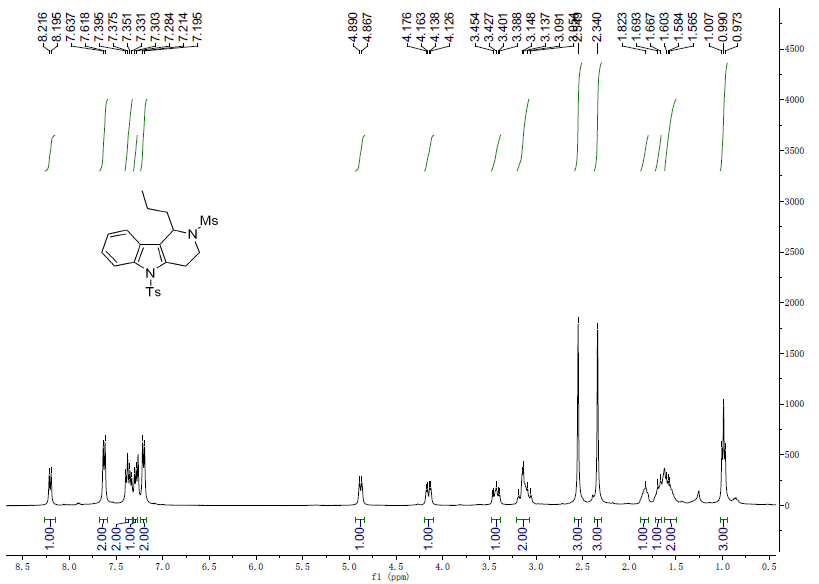


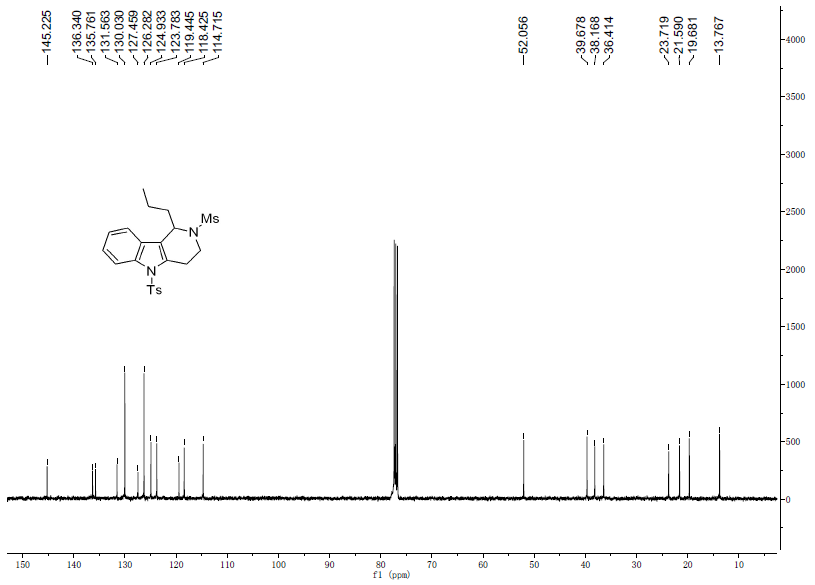

Supplement: Supplementary Information [file srep13516-s1.doc]
