# Supplementary material for: Synthesis of Structurally Diverse 2,3-Fused Indoles via Microwave-Assisted AgSbF6-Catalysed Intramolecular Difunctionalization of o-Alkynylanilines
Source: Sci Rep. 2015 Aug 27;5:13516. doi: 10.1038/srep13516 (PMC4550932; doi:10.1038/srep13516)

checkCIF/PLATON report

You have not supplied any structure factors. As a result the full set of tests cannot be run.

THIS REPORT IS FOR GUIDANCE ONLY. IF USED AS PART OF A REVIEW PROCEDURE FOR PUBLICATION, IT SHOULD NOT REPLACE THE EXPERTISE OF AN EXPERIENCED CRYSTALLOGRAPHIC REFEREE.

No syntax errors found.   CIF dictionary   Interpreting this report

Datablock: shelxl

```
Bond precision:  C-C = 0.0053 A          Wavelength=0.71073

Cell:           a=8.4379(17)      b=11.874(2)      c=13.696(3)
                alpha=99.40(3)    beta=90.49(3)    gamma=98.37(3)
Temperature:    113 K

                Calculated                 Reported
Volume          1338.6(5)                  1338.7(5)
Space group     P -1                       P-1
Hall group      -P 1                       ?
Moiety formula  C30 H28 N2 O4 S3           ?
Sum formula     C30 H28 N2 O4 S3           C30 H28 N2 O4 S3
Mr              576.72                     576.72
Dx,g cm-3       1.431                      1.431
Z               2                          2
Mu (mm-1)       0.318                      0.318
F000            604.0                      604.0
F000'           604.98
h,k,lmax        10,14,16                   10,14,16
Nref            4712                       4690
Tmin,Tmax       0.938,0.963                0.939,0.963
Tmin'           0.938

Correction method= # Reported T Limits: Tmin=0.939 Tmax=0.963
AbsCorr = ?

Data completeness= 0.995          Theta(max)= 25.010

R(reflections)= 0.0453( 2278)     wR2(reflections)= 0.1334( 4690)

S = 0.940                   Npar= 354
```

The following ALERTS were generated. Each ALERT has the format
test-name_ALERT_alert-type_alert-level.
Click on the hyperlinks for more details of the test.

Alert level C

```
PLAT026_ALERT_3_C Ratio Observed / Unique Reflections too Low ....         49 %
PLAT052_ALERT_1_C Info on Absorption Correction Method Not Given .     Please Do !
PLAT230_ALERT_2_C Hirshfeld Test Diff for    S3     --  O4      ..        5.4 su
PLAT340_ALERT_3_C Low Bond Precision on  C-C Bonds ...............     0.0053 Ang.
```

Alert level G

```
PLAT005_ALERT_5_G No _iucr_refine_instructions_details  in the CIF     Please Do !
PLAT066_ALERT_1_G Predicted and Reported Tmin&Tmax Range Identical          ? Check
PLAT093_ALERT_1_G No su's on H-positions, refinement reported as .      mixed Check
PLAT154_ALERT_1_G The su's on the Cell Angles are Equal ..........    0.03000 Degree
PLAT793_ALERT_4_G The Model has Chirality at C13     (Centro SPGR)          R Verify
PLAT899_ALERT_4_G SHELXL97   is Deprecated and Succeeded by SHELXL       2014 Note
```

```
   0 ALERT level A = Most likely a serious problem - resolve or explain
   0 ALERT level B = A potentially serious problem, consider carefully
   4 ALERT level C = Check. Ensure it is not caused by an omission or oversight
   6 ALERT level G = General information/check it is not something unexpected

   4 ALERT type 1 CIF construction/syntax error, inconsistent or missing data
   1 ALERT type 2 Indicator that the structure model may be wrong or deficient
   2 ALERT type 3 Indicator that the structure quality may be low
   2 ALERT type 4 Improvement, methodology, query or suggestion
   1 ALERT type 5 Informative message, check
```

It is advisable to attempt to resolve as many as possible of the alerts in all categories. Often the minor alerts point to easily fixed oversights, errors and omissions in your CIF or refinement strategy, so attention to these fine details can be worthwhile. In order to resolve some of the more serious problems it may be necessary to carry out additional measurements or structure refinements. However, the purpose of your study may justify the reported deviations and the more serious of these should normally be commented upon in the discussion or experimental section of a paper or in the "special_details" fields of the CIF. checkCIF was carefully designed to identify outliers and unusual parameters, but every test has its limitations and alerts that are not important in a particular case may appear. Conversely, the absence of alerts does not guarantee there are no aspects of the results needing attention. It is up to the individual to critically assess their own results and, if necessary, seek expert advice.

Publication of your CIF in IUCr journals

A basic structural check has been run on your CIF. These basic checks will be run on all CIFs submitted for publication in IUCr journals (*Acta Crystallographica*, *Journal of Applied Crystallography*, *Journal of Synchrotron Radiation*); however, if you intend to submit to *Acta Crystallographica Section C* or *E*, you should make sure that full publication checks are run on the final version of your CIF prior to submission.

Publication of your CIF in other journals

Please refer to the *Notes for Authors* of the relevant journal for any special instructions relating to CIF submission.

PLATON version of 21/04/2015; check.def file version of 09/03/2015

Datablock shelxl - ellipsoid plot

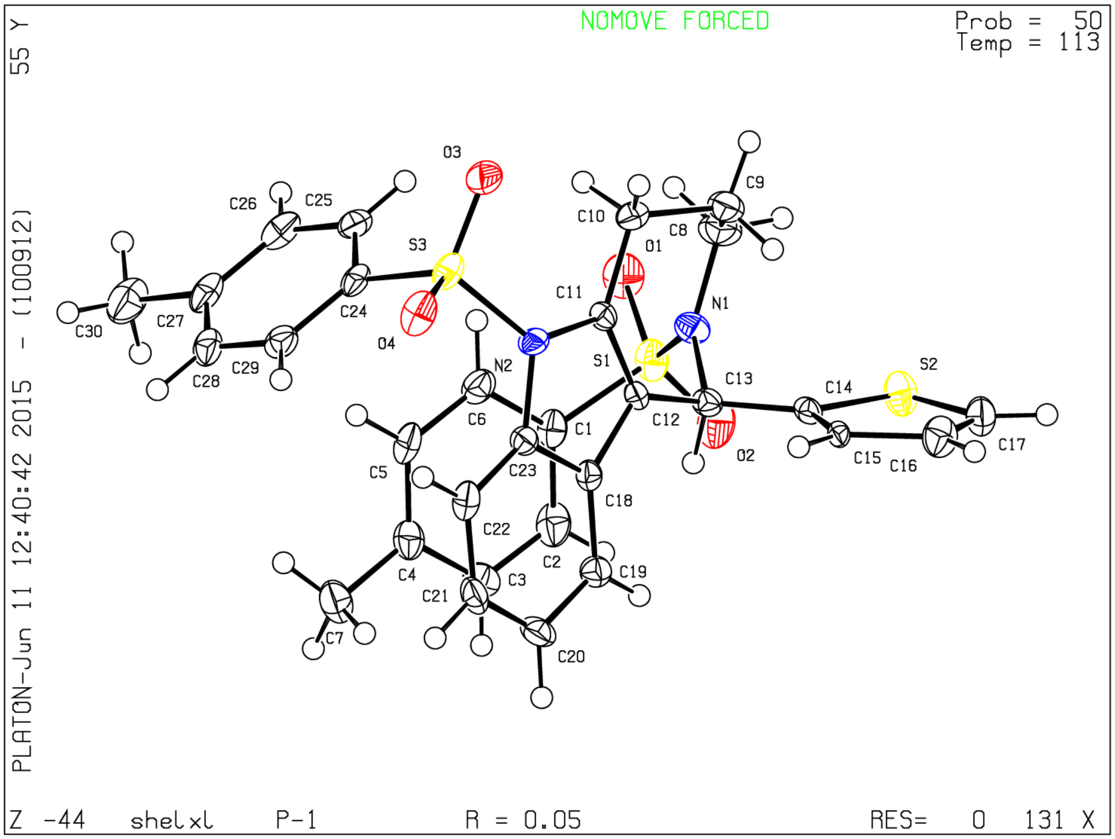

Supplement: Supplementary File 3 [file srep13516-s4.pdf]
